# Comparison of miRNA cargo in human adipose-tissue *vs*. amniotic-membrane derived mesenchymal stromal cells extracellular vesicles for osteoarthritis treatment

**DOI:** 10.20517/evcna.2021.11

**Published:** 2021-08-03

**Authors:** Enrico Ragni, Carlotta Perucca Orfei, Andrea Papait, Laura de Girolamo

**Affiliations:** ^1^IRCCS Istituto Ortopedico Galeazzi, Laboratorio di Biotecnologie Applicate all’ Ortopedia, Milan I-20161, Italy.; ^2^Centro di Ricerca E. Menni, Fondazione Poliambulanza Istituto Ospedaliero, Brescia I-25124, Italy.; ^3^Department of Life Science and Public Health, Università Cattolica del Sacro Cuore, Rome I-00168, Italy.

**Keywords:** Extracellular vesicles, miRNAs, mesenchymal stromal cells, adipose tissue, amniotic membrane, osteoarthritis, joint diseases, regenerative medicine

## Abstract

**Aim:**

Mesenchymal stromal cells (MSCs) emerged as a promising therapeutic option for osteoarthritis (OA) management, in particular those isolated from adipose tissue (hASCs) and amniotic membrane (hAMSCs). The cartilage protective and immunomodulatory features of hASCs and hAMSCs are ascribed to secreted factors, including extracellular vesicles (EVs) and embedded miRNAs. The purpose of this study was to compare EVs and shuttled miRNAs from both MSC types and discuss them in the frame of OA pathological tissues.

**Methods:**

Human hASCs and hAMSCs were analyzed by flow cytometry. EVs were analyzed by flow cytometry, nanoparticle tracking analysis, and electron microscopy. High-throughput qRT-PCR miRNA data available in the literature were compared. Abundant miRNAs and their experimentally validated targets were associated with those reported to drive OA pathology at cartilage, synovia, and macrophage levels. Four tools (Genorm, Normfinder, BestKeeper, and Delta Ct) were used to identify EVs stable reference genes.

**Results:**

EVs did not show phenotypical or dimensional differences between the two sources, with hAMSCs releasing more particles. In total, 307 EV miRNAs were identified, with 306 shared. Several of the most abundant miRNAs target OA-driving factors and are involved in cartilage and synovia protective mechanisms, with hAMSC-EVs’ preponderance for M2 anti-inflammatory macrophage commitment. miR-34a-5p emerged as the most stable reference gene.

**Conclusion:**

Both hASCs and hAMSCs release EVs enriched in joint-protective and anti-inflammatory miRNAs, supporting their use for treatment of joint diseases. Future comparative clinical studies would be needed to test whether hAMSCs’ higher EV secretion and enhanced M2 macrophage polarizing miRNA cargo allow for potentially increased OA therapeutic features.

## INTRODUCTION

Osteoarthritis (OA) is a common progressive multifactorial joint disease affecting 7% of the global population and one of the leading causes of disability in older adults^[1]^. OA pathogenesis involves mechanical, inflammatory, and metabolic factors, eventually leading to an imbalance between the repair and destruction of joint tissues, such as cartilage and synovium^[2]^. Chondrocytes in the damaged cartilage enhance extracellular matrix degradation and released products, together with pro-inflammatory mediators, act on synoviocytes and inflammatory cells of the synovium, stimulating pro-inflammatory responses^[2]^. To date, early-stage treatments, such as pharmacological methods, address OA symptoms by reducing inflammation and pain^[3]^. Consequently, these conservative approaches are not effective in disease amelioration but only postpone the need of joint replacement, opening the search for novel and biological therapeutic strategies.

In this scenario, mesenchymal stromal cell (MSC)-based treatments emerged as a promising new approach for OA^[4]^. MSCs are multipotent cells that can be found in many different stromal tissues, both adult and perinatal. MSCs, in the case of injury or disease, secrete bioactive factors, both free as cytokines/chemokines and embedded in extracellular vesicles (EVs) as miRNAs, with immunomodulatory and trophic functions^[5]^. Human adipose tissue/stromal vascular fraction (SVF) and amniotic membrane (AM) have gained particular attention as tissue sources for MSCs, and, despite their different origins, they share some crucial advantages. Both sources can be easily harvested without ethical controversy since they are obtained from biological waste after liposuction or birth, respectively. In addition, they have a higher cell recovery [1 × 10^5^ human adipose-derived MSCs (hASCs)^[6]^ or 1 × 10^6^ human amniotic membrane-derived MSCs (hAMSCs)^[7]^ per tissue gram]^[8]^ with respect to bone marrow [3 × 10^3^ human bone marrow-derived MSCs^[9]^ per mL]^[10]^. Adipose tissue has been largely studied in more than 20 OA-related clinical trials in the form of expanded hASCs^[11,12]^, unprocessed SVF, or micro/nano-fragmented adipose tissue. On the contrary, AM is still in its infancy, with few reported trials using both allogenic hAMSCs^[13]^ and most frequently amniotic suspension allografts^[14-16]^. Notably, the majority of these clinical studies reported significant improvements in terms of pain and knee function. However, the substantial lack of consistency in terms of treatment protocols and assessment of clinical outcomes prevents an efficient comparison of these data, and therefore it is hard to determine the most effective source of MSCs^[17]^.

Beyond a missing direct clinical comparison of hASCs *vs*. hAMSCs in the clinical OA setting, basic research is also very scarce. In fact, several reviews describe the different MSC types for OA therapy^[18]^, with abundance of *in vitro* and pre-clinical results, but very few data characterizing hASCs *vs*. hAMSCs in the same study are available. Topoluk *et al*.^[19]^showed that hAMSCs have a greater differentiation potential toward bone and cartilage compared with hASCs. In addition, in a sophisticated *in vitro* coculture model of patient-matched human OA cartilage and synovium, hAMSCs resulted more chondroprotective and more effective at reducing the OA pro-inflammatory: anti-inflammatory (M1:M2) synovial macrophage ratio^[20]^. These features might be ascribed to different secretory activity, including EVs and their miRNAs. Consistently, whole secretome and MSC-EVs have been reported to mimic and even surpass MSCs’ protective ability in the OA setting, and this was seen for both hAMSCs^[21]^ and hASCs^[22-25]^ whose EVs were reported *in vitro* to polarize macrophages by upregulating the expression of M2 markers^[26,27]^. This immunomodulatory feature was also recently described for other MSC-EV types, such as embryonic stem cell-, bone marrow-, and Wharton’s jelly-derived ones, during cartilage repair in animal models^[28-30]^. Notably, differences in miRNA cargo were postulated to account for divergent immunomodulatory and trophic properties of EVs from alternative sources^[31]^, although the relevance of these differences have not been discussed for OA.

Thus, the goal of this study was to compare cells, EVs, and embedded miRNA cargo sifting data previously obtained in our laboratory with identical technical approaches and platforms (hASCs^[32]^ and hAMSCs^[21]^). Whole miRNomes and differentially expressed players were analyzed by bioinformatics for miRNA-mRNA interactions using expression data from OA-affected tissues and cells. These results give the molecular basis for future clinical investigations that directly compare hAMSCs and hASCs within the same study for OA treatment.

## METHODS

### hASCs and hAMSCs isolation and culture

Adipose tissue was obtained as waste material from three female donors (54 years ± 8 years) who underwent liposuction for aesthetic purposes and gave their consent to donate waste biological material for research purposes. hASCs were isolated as previously described^[32]^. After selection for plastic adhesion, cells were expanded in DMEM high glucose (Sigma Aldrich, St. Louis, MO, USA) supplemented with FBS (GE Healthcare, Piscataway, NJ, USA) at 37 °C in a humidified atmosphere with 5% CO_2_ and used at Passage 3. Human term placentas were collected as waste material from three healthy women. hAMSCs were isolated as previously described^[21]^. Cells were then expanded in CHANG C medium (Irvine Scientific, Irvine, CA, USA) at 37 °C in a humidified atmosphere with 5% CO_2_ and used at Passage 2.

### hASCs and hAMSCs characterization by flow cytometry

hASCs and hAMSCs were analyzed with a CytoFLEX flow cytometer (Beckman Coulter, Fullerton, CA, USA), collecting at least 30,000 events. Antibodies were used in two panels: (1) anti-CD44-PE-Vio770 (REA690, Miltenyi Biotec, Bergisch Gladbach, Germany), CD73-PE (REA804, Miltenyi), and CD90-FITC (REA897, Miltenyi); and (2) CD31-PerCP-Vio700 (REA730, Miltenyi), CD34-FITC (AC136, Miltenyi), and CD45-PE-Vio770 (REA747, Miltenyi).

### Cell culture supernatant collection

hASCs and hAMSCs at 90% confluence were washed three times with PBS to remove residues of exhausted media, and fresh media without supplements were added at 0.07 mL/cm^2^. After 48 h, cells were detached and counted with an automatic cell counter, NucleoCounter NC-3000 (ChemoMetec, Allerod, Denmark), while media were collected and serially centrifuged (376 g, 1000 g, 2000 g, and twice at 4000 g, 15 min each) at 4 °C to eliminate debris, floating cells, and apoptotic bodies.

### EVs detection by nanoparticle tracking analysis

EVs in the serially centrifuged supernatant (1:2 diluted in PBS for hASCs and 1:10 diluted for hAMSCs) were visualized by the NanoSight LM10-HS system (NanoSight Ltd, Amesbury, UK). Five 30 s recordings were performed, and the data were analyzed by nanoparticle tracking analysis (NTA) software, providing high-resolution particle size distribution profiles and concentration measurements. The number of EVs per cell for both hASCs and hAMSCs was calculated.

### EVs characterization by flow cytometry

NTA data were used to obtain supernatant samples with similar numbers of EVs (approximately 1-2 × 10^6^ EVs) in a final volume of 20 µL, either PBS or PBS + 0.1 μM CFSE, and incubation was performed in the dark at 37 °C for 1 h. CFSE-unlabeled samples were stored at 4 °C, whereas CFSE-labeled samples were stained for 30 min at 4 °C in the dark with 1 μL of the following APC-conjugated Abs: anti-CD9 (312107, BioLegend, San Diego, CA, USA), CD63 (353007, BioLegend), CD81 (349509, BioLegend), anti-CD44 (338805, BioLegend), CD73 (344005, BioLegend), and CD90 (328113, BioLegend). Antibodies were used individually after being centrifuged at 16,000 g for 20 min at 4 °C to remove debris. After incubation, PBS to a final volume of 200 µL was added to both stained (CFSE and CFSE + Abs) and unstained samples, and events collection was performed with a CytoFLEX flow cytometer at 10 μL/min flow rate. To identify CFSE positive EVs, a first gate in the FITC channel was performed using unstained EVs as negative samples. FITC + events were used to create APC-positive and -negative gates to visualize in CFSE + Abs-treated samples the EVs harboring the respective antigens. The flow cytometer was previously calibrated with reference Megamix-Plus SSC beads (Biocytex, Marseille, France) composed of FITC fluorescent spheres (160, 200, 240, and 500 nm).

### EV characterization by transmission electron microscopy

Thirteen milliliters of serially centrifuged supernatant were 1:2 diluted with PBS and centrifuged at 100,000 g for 9 h at 4 °C in a 70.1Ti rotor (Beckman Coulter, Fullerton, CA, USA). After pellet suspension in PBS (100 µL), 5 μL were absorbed for 10 min at RT on formvar carbon-coated grids and excess liquid removed by filter paper. Uranyl acetate aqueous suspension (2%, 10 min) gave the negative staining and excess liquid was removed by filter paper. Eventually, the grid was dried at RT. Images were acquired with a TALOS L120C transmission electron microscopy (TEM) (Thermo Fisher Scientific, Waltham, MA, USA) at 120 kV.

### EV miRNA retrieval and normalization

EV miRNA qRT-PCR data were obtained as previously published^[21,32]^. Briefly, EV pellets were dissolved in Trizol reagent (Sigma Aldrich) and 6 pg of a nonhuman synthetic miRNA spike-in (Arabidopsis thaliana ath-miR-159a) were added to monitor the technical variability during the whole detection procedure and during subsequent reactions for the eventual equalization of panels A and B of the OpenArray® platform (Thermo Fisher Scientific, Waltham, MA, USA). RNA was extracted with miRNeasy and RNeasy CleanUp Kits to isolate RNA enriched in small molecules < 200 nt (Qiagen, Hilden, Germany), and cDNAs were obtained by standard reverse transcription, with preamplification performed with A and B independent kits, followed by RT-PCR analysis with the QuantStudio^TM^ 12 K Flex OpenArray® Platform (QS12KFlex) on A and B miRNA panels, which together cover 754 human miRNA sequences from the Sanger miRBase v21. Only miRNAs present in all three isolates and shared between hASC-EVs and hAMSC-EVs were considered for normalization, which was performed using the global mean strategy^[33]^. For the final analysis, miRNAs with STD > 2 in either hASC-EVs or hAMSC-EVs were excluded to avoid strong donor-dependent variability and define a constant tissue-type specific message. The genetic weight was calculated with the ΔC_RT_ method between normalized miRNAs (using the lowest normalized C_RT_ as milestone for ΔC_RT_ calculations between miRNAs), giving an arbitrary value of 1 to the lowest normalized C_RT_ and 2^-ΔCRT^ value to the following candidates. Thereafter, the sum was calculated and the weight for each miRNA was obtained with the formula: (arbitrary miRNA value/sum of arbitrary values) × 100.

### Assessment of miRNA RG stability

miRNA expression stability was evaluated on the molecules lying in the first quartiles of expression according to four gold-standard statistical approaches: geNorm^[34]^, NormFinder^[35]^, BestKeeper^[36]^, and the comparative Delta Ct method^[37]^. The overall performance of the miRNA RGs was evaluated by a global approach relying on the geometric mean of the rankings given by each analysis obtained with the RefFinder platform^[38]^.

### Hierarchical clustering and principal component analysis

Heat maps showing hierarchical clustering and principal component analysis (PCA) plots were generated on normalized C_RT_ values with ClustVis package (https://biit.cs.ut.ee/clustvis/)^[39]^. The only miRNA expressed in hAMSC-EVs and absent in hASC-EVs was tagged with a C_RT_ value of 30 in hASC-EVs. Data pre-processing for the PCA method included: (1) no transformation; (2) no centering; (3) no scaling applied to rows; and (4) SVD with imputation. Heat map clustering options included: both rows and columns were clustered using correlation distance and average linkage.

### EV miRNA target identification

miRTarBase database (https://mirtarbase.cuhk.edu.cn/~miRTarBase/miRTarBase_2019/php/index.php)^[40]^ was used to retrieve the EV miRNA targets, selecting only those interactions reported to be validated by strong experimental evidence (reporter assay, Western blot, and qPCR).

### Statistical analysis

Student’s two-tailed *t*-test was used to compare cell density values, EV release per cell type, and hASC-EV and hAMSC-EV normalized miRNA C_RT_ values, with *P*-value set at *P* < 0.05 for significance.

## RESULTS

### Phenotype characterization of hASCs, hAMSCs and secreted EVs

**Figure 1:**
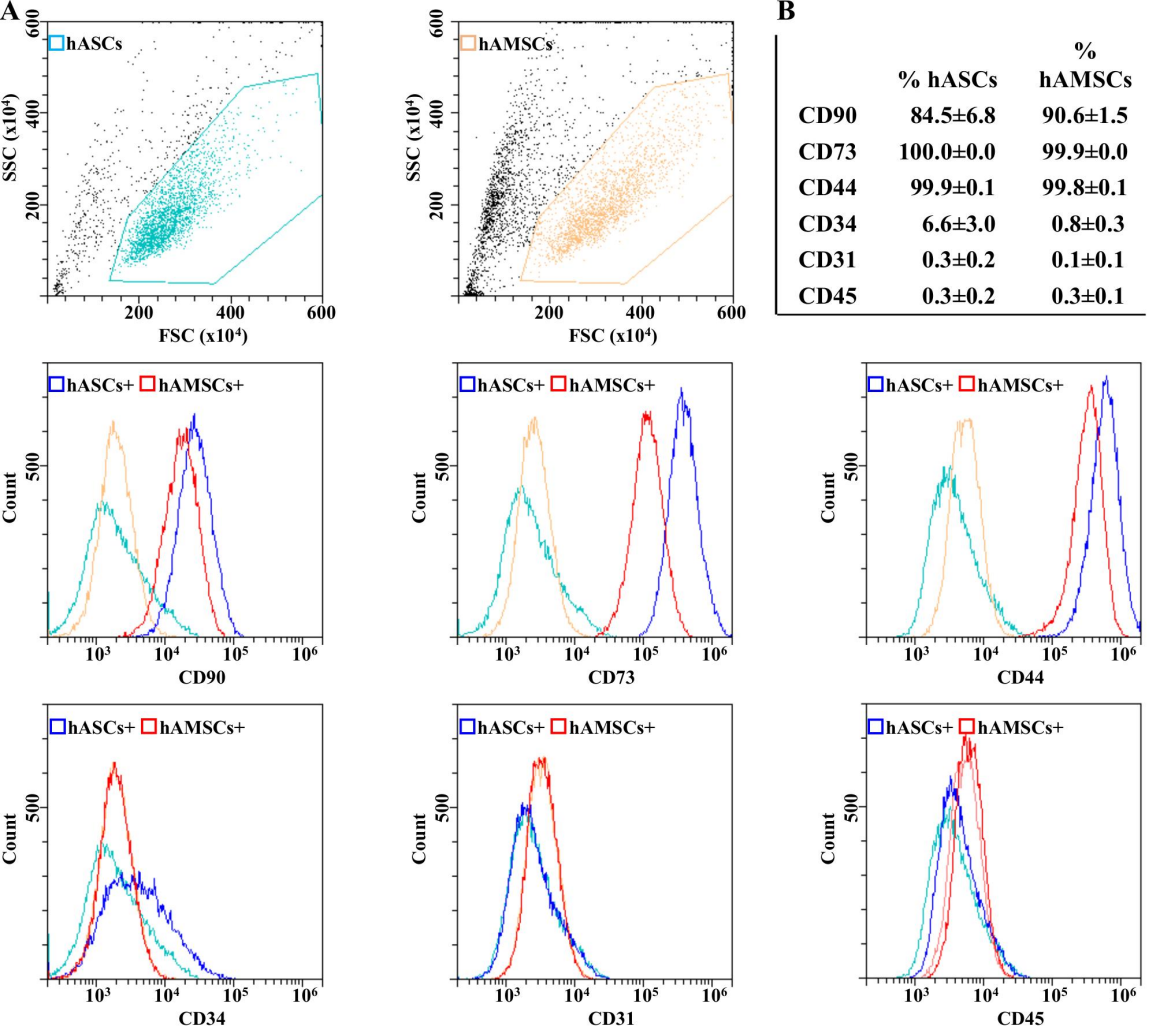
hASCs and hAMSCs immunophenotype. (A) Flow cytometry analysis of hASC and hAMSCs for MSC (CD44/73/90) and hematoendothelial (CD31/34/45) markers. Representative plots are shown. (B) Percentage of positivity for analyzed markers obtained by averaging the three donors. Weak CD34 positivity is a landmark for hASCs identity.

Both hASCs and hAMSCs were simultaneously characterized by flow cytometry for MSC and hematoendothelial markers [Figure 1]. All donors resulted strongly positive for stromal CD44-73-90 and negative for CD31 and CD45. CD34 resulted absent in hAMSCs and weakly positive in hASCs (6.6% ± 3.0%), as previously reported^[41]^. At Passage 3, hAMSCs reached a significantly higher confluence per cm^2^ (26.8 × 10^3^ ± 2.9* vs*. 7.1 × 10^3^ ± 0.2, *P*-value of 0.0003).

**Figure 2:**
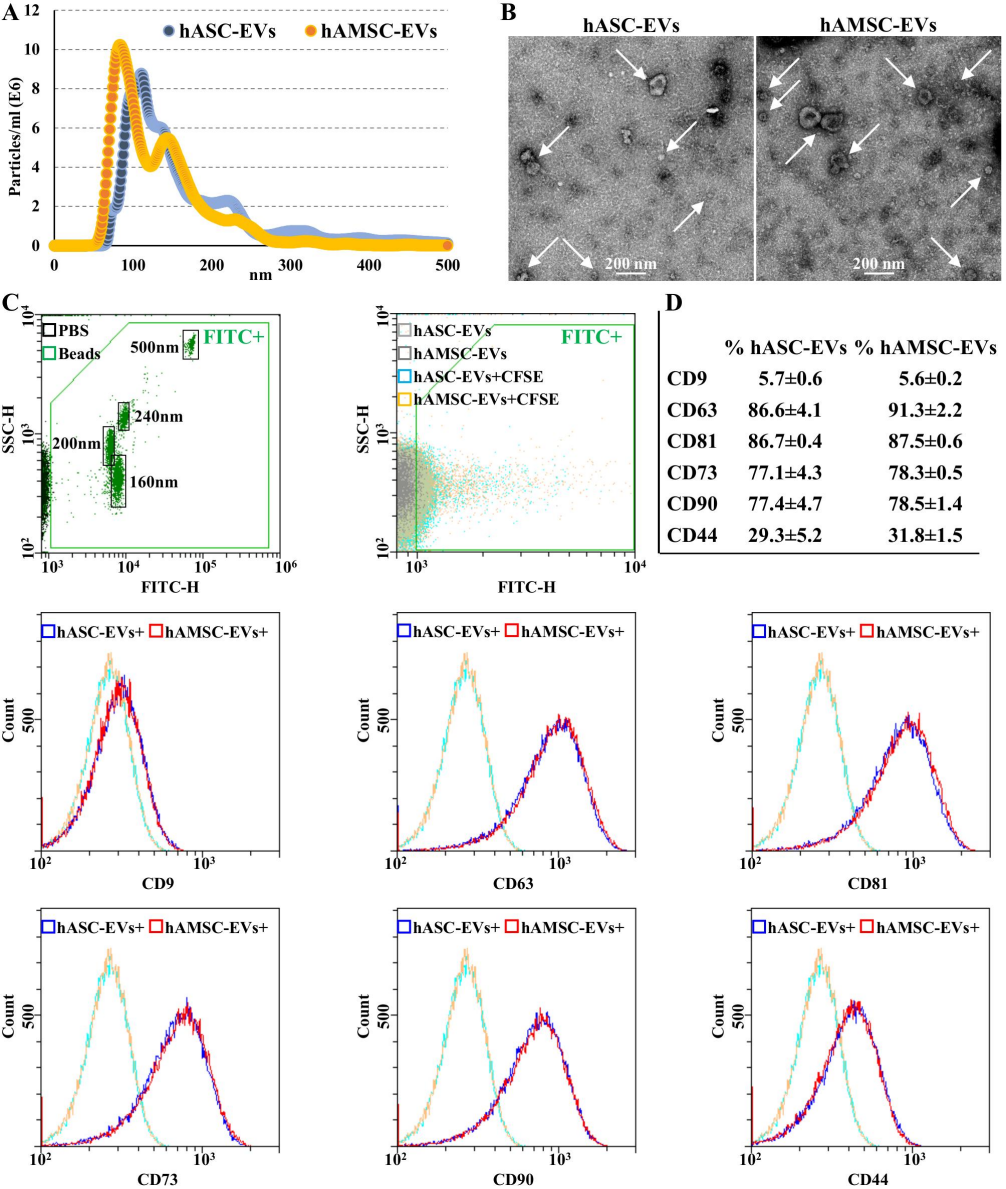
hASC-EVs and hAMSC-EVs characterization. (A) EV size distribution by NanoSight particle tracking analysis for both hASC-EVs (blue dots) and hAMSC-EVs (orange dots). Plots show merged data of the three donors for each MSC type. (B) Transmission electron micrographs of EVs showing particles with characteristic cup-shaped morphology. Representative donors are shown. (C) The resolution of the FITC-fluorescent reference nanobeads (160, 200, 240, and 500 nm) indicates the flow cytometer performance in light scattering at default settings. After flow cytometer calibration, CFSE-stained EVs can be identified and gated in the FITC channel (FITC+ gate) *vs*. unstained particles. After gating, with respect to Ab-unstained samples, both hASC-EVs and hAMSC-EVs showed the presence of EV-defining molecules CD63 and CD81, while CD9 staining gave a very weak signal. Both hASC-EVs and hAMSC-EVs were also positive for MSC markers CD73 and CD90. CD44 labeling allowed a complete peak shift of the population, although without a sharp peak separation. Representative cytograms are presented. (D) Percentage of positivity for analyzed markers obtained averaging the three donors. CFSE: Carboxyfluorescein succinimidyl ester; EVs: extracellular vesicles; FITC: fluorescein isothiocyanate; hASCs: adipose-derived mesenchymal stromal cells; hAMSCs: amniotic membrane-derived mesenchymal stromal cells.

In our experimental conditions, hASCs and hAMSCs released EVs in the range of 1.6 × 10^3^ ± 0.4 and 4.7 × 10^3^ ± 1.9 particles per cell (*P*-value of 0.01461) in 48 h, respectively. Overall, hAMSCs released 11.1-fold more EVs per surface unit (*P*-value of 0.00323) with respect to hASCs. NTA analysis confirmed the nanometer-scale range for both EV types [Figure 2A], the average size being 102 ± 8 nm and 96 ± 25 nm (mode ± SD) for hASC-EVs and hAMSC-EVs, respectively, with enrichment in small particles (75.1% ± 0.5% and 87.7% ± 4.4% below 200 nm). The dimensional range was confirmed by TEM [90 ± 37 and 90 ± 29 nm (mode ± SD) for hASC-EVs and hAMSC-EVs, respectively] [Figure 2B] and flow cytometry, after instrument calibration with fluorescent nanobeads (160, 200, 240, and 500 nm) [Figure 2C]. Both EV types resulted strongly positive for EV markers CD63 and CD81, while CD9 resulted barely detectable [Figure 2D]. In addition, MSC markers CD73 and CD90 were highly expressed, while CD44 appeared less abundant, although the whole peak shift suggests a homogeneous dim population.

### EV-associated miRNAs

**Figure 3:**
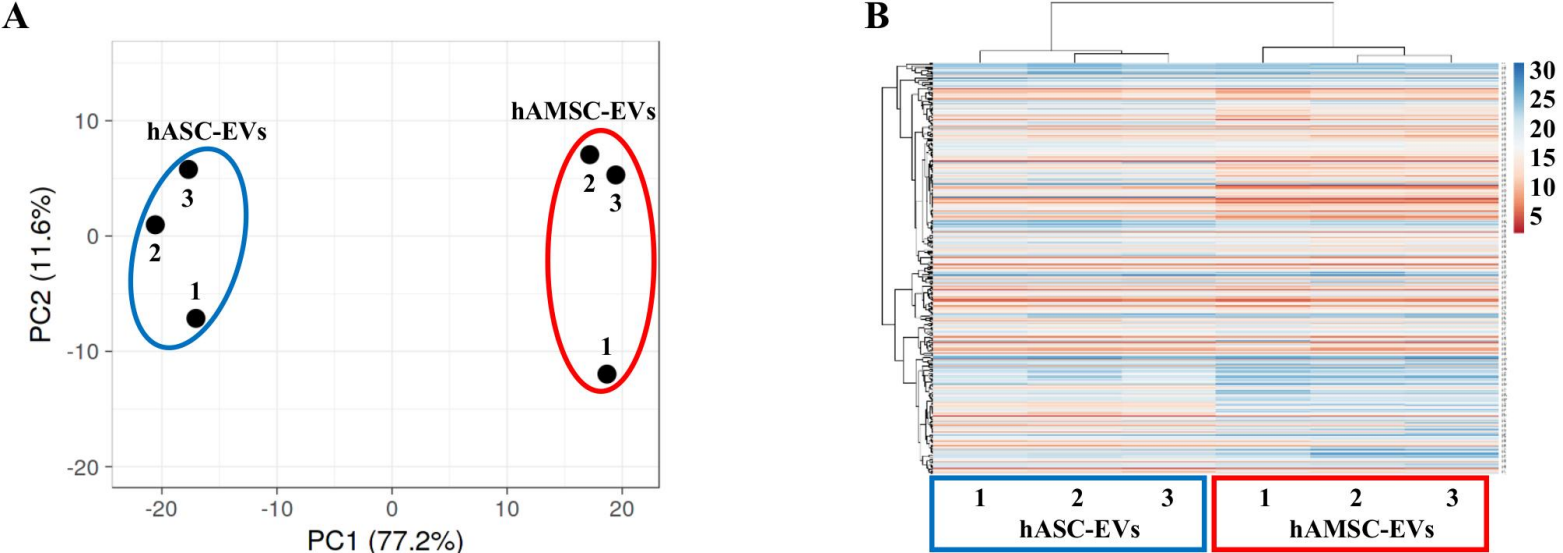
Comparison of hASC-EV- and hAMSC-EV-embedded miRNA abundance. (A) Principal component analysis of the normalized C_RT_ values of miRNAs. X and Y axes show Principal Components 1 and 2, which explain 77.2% and 11.6% of the total variance, respectively. (B) Heat map of hierarchical clustering analysis of the normalized C_RT_ values of detected miRNAs with sample clustering tree at the top. The color scale of normalized C_RT_ values reflects the absolute expression: red shades indicate high expression levels, while blue shades indicate low expression levels.

In total, 306 and 307 miRNAs were detected and highly shared in hASC-EVs and hAMSC-EVs, respectively (Supplementary Table 1), with miR-490-3p present only in hAMSC-EVs. Nevertheless, both PCA and hierarchical clustering were able to sharply cluster and group the EV types based on MSC origin [Figure 3A and B]. Accordingly, correlation analysis emphasized consistent homogeneity for both hASC-EV (*R*^2^ of 0.96 ± 0.01) and hAMSC-EV (0.95 ± 0.02) donors, thus allowing to average EV miRNA C_RT_ values for each MSC type [Supplementary Table 2]. A reduced *R*^2^ value (0.81) for averaged miRNAs emerged, confirming differential incorporation of the same miRNAs into EVs. Consistently, 47 candidates resulted upregulated (ratio > 2 between averaged values with *P*-value < 0.05 between populations) in hASC-EVs, with miR-30d-3p (ratio 998.30), miR-601 (179.44), and miR-95-3p being the ones with the highest fold change Supplementary Table 2]. On the contrary, 71 molecules resulted downregulated (ratio < 0.5 with *P*-value < 0.05), and miR-372-3p (0.04), miR-30d-5p (0.01), and miR-146a-5p (0.0001) were the ones with the highest fold change [Supplementary Table 2].

To attribute a biological significance to detected or differentially expressed EV miRNAs, several parameters were taken into consideration: (1) even for the most abundant miRNA, in MSC-EVs no more than one copy per vesicle is present^[42]^; (2) a minimal ratio of 100 MSC-EVs per target cell is needed to allow transfer of abundant miRNAs^[43]^; and (3) in several cell types, including synoviocytes and chondrocytes, only a few thousand MSC-EVs can be incorporated in a day^[32,44]^. Therefore, only miRNAs laying in the first quartile of expression of both MSC-EV types were considered for further analysis. In total, 77 miRNAs defined each quartile [Table 1 and Supplementary Table 3 for those most abundantly (≥ 1% genetic weight) expressed], covering 97.90% and 98.19% of hASC-EVs and hAMSC-EVs genetic weight, respectively. Out of the selected molecules, 64 were shared, with 13 hASC-EVs and 13 hAMSC-EVs first quartile specific, collectively defining a group of 90 miRNAs. Within these candidates, 13 were significantly upregulated in hASC-EVs and 27 were downregulated, with miR-30d-3p being the most induced (ratio of 998.30) and miR-146a-5p the most reduced (0.0001). Sifting experimentally validated miRNA-mRNA interactions, first quartile hASC-EV miRNAs target 1575 univocal genes, while first quartile hAMSC-EVs 1543 transcripts. A high correlation emerged, with 1463 genes shared, 112 hASC-EV specific, and 80 hAMSC-EV specific. Overall, the 90 miRNAs resulted to target 1655 mRNAs [Supplementary Tables 4 and 5]. In particular, 372 and 364 mRNAs are specifically targeted by 13 hASC-EV and 27 hAMSC-EV upregulated miRNAs, respectively [Supplementary Table 5]. These results suggest that single miRNA modulation of abundance between hASC-EVs and hAMSC-EVs, rather than different targets, frame the source-specific influence on target cells and tissues.

**Table 1:** miRNAs differential expression and genetic weight in hASC-EVs *vs*. hAMSC-EVs first quartile of expression

**miRBase ID**	**hASC-EVs % genetic weight**	**hAMSC-EVs % genetic weight**	**hASC-EVs *vs*. hAMSC-EVs ratio**	***P*** **-value**
miR-30d-3p	**0.11416**	0.00008	***998.30***	**0.00017**
miR-1260a	**0.16830**	0.00326	***35.56***	**0.00411**
let-7c-5p	**0.18163**	0.00523	***23.93***	**0.00017**
miR-195-5p	**0.12349**	0.00494	***17.20***	**0.00040**
miR-138-5p	**0.19066**	0.02287	***5.74***	**0.01210**
miR-27a-3p	**0.97429**	**0.12009**	***5.59***	**0.00752**
miR-29b-3p	**0.08998**	0.01442	***4.30***	**0.04382**
miR-125b-5p	**16.02689**	**2.67123**	***4.13***	**0.00278**
let-7a-5p	**0.18804**	0.03373	3.84	0.11638
miR-663b	**0.13204**	0.02513	3.62	0.09212
miR-30a-5p	**0.65026**	**0.14730**	***3.04***	**0.01300**
miR-224-5p	**0.54302**	**0.12461**	***3.00***	**0.02570**
miR-27b-3p	**0.35497**	**0.09320**	***2.62***	**0.04494**
miR-23a-3p	**0.10360**	0.02821	***2.53***	**0.03458**
miR-99b-5p	**1.57909**	**0.48102**	***2.26***	**0.02318**
miR-29c-3p	**0.43601**	**0.15005**	2.00	0.09116
miR-130a-3p	**0.63248**	**0.25032**	1.74	0.10744
miR-25-3p	**0.19648**	**0.08104**	1.67	0.06448
miR-218-5p	**0.76618**	**0.32756**	1.61	0.05748
miR-199a-3p	**0.89860**	**0.38569**	1.60	0.15468
miR-221-3p	**5.85265**	**2.67864**	1.50	0.15261
miR-143-3p	**0.11078**	0.05134	1.49	0.31951
miR-28-5p	**0.13638**	**0.06363**	1.48	0.45152
miR-26a-5p	**0.56739**	**0.29501**	1.32	0.56892
miR-31-3p	**0.33582**	**0.18152**	1.27	0.41762
miR-193b-3p	**4.27450**	**2.36882**	1.24	0.62762
miR-361-5p	**0.08915**	0.05027	1.22	0.20735
miR-21-5p	**6.27271**	**3.80748**	1.13	0.57381
miR-26b-5p	**0.14119**	**0.08680**	1.12	0.65301
miR-145-5p	**1.62348**	**1.02066**	1.10	0.82372
miR-148a-3p	**0.08532**	0.05467	1.07	0.89270
miR-34a-3p	**0.08280**	0.05310	1.07	0.60948
miR-100-5p	**3.60273**	**2.31365**	1.07	0.82076
miR-92a-3p	**1.59375**	**1.03563**	1.06	0.86197
miR-99a-5p	**3.56958**	**2.37924**	1.03	0.91251
miR-214-3p	**0.92173**	**0.62222**	1.02	0.94421
miR-106b-5p	**0.33427**	**0.22596**	1.02	0.91048
miR-365a-3p	**0.18038**	**0.12264**	1.01	0.95600
miR-16-5p	**0.44209**	**0.30443**	1.00	1.00000
miR-296-5p	**0.16445**	**0.11691**	0.97	0.92733
miR-22-3p	**0.15812**	**0.11945**	0.91	0.52628
miR-197-3p	**0.41632**	**0.31531**	0.91	0.84086
miR-34a-5p	**0.69052**	**0.55408**	0.86	0.39612
miR-152-3p	**0.52453**	**0.42933**	0.84	0.16680
miR-30b-5p	**2.34419**	**1.95455**	0.83	0.38278
miR-29a-3p	**0.98107**	**0.82198**	0.82	0.66389
miR-328-3p	**0.56739**	**0.48977**	0.80	0.65789
miR-10a-5p	**0.24527**	**0.21289**	0.79	0.67981
miR-222-3p	**5.20208**	**4.86634**	0.74	0.29980
miR-30c-5p	**3.05765**	**3.00737**	0.70	0.07172
miR-24-3p	**17.21693**	**17.70194**	0.67	0.01369
miR-130b-3p	**0.07797**	**0.08110**	0.66	0.10380
miR-132-3p	**0.48266**	**0.52456**	0.63	0.25039
miR-127-3p	**0.59698**	**0.66920**	0.61	0.11546
miR-382-5p	**0.59149**	**0.67245**	0.61	0.25318
miR-20a-5p	**1.44302**	**1.66075**	0.60	0.00791
miR-193a-5p	**0.16033**	**0.18636**	0.59	0.11502
miR-331-3p	**0.28109**	**0.32832**	0.59	0.26238
miR-28-3p	**0.10384**	**0.12264**	0.58	0.01535
miR-483-5	0.07480	**0.10421**	0.49	0.32196
miR-409-3p	**0.28109**	**0.41769**	0.46	0.24588
miR-532-5p	**0.08053**	**0.12435**	***0.45***	***0.00300***
miR-93-5p	0.06363	**0.10389**	***0.42***	***0.02717***
miR-654-5p	0.03629	**0.06309**	***0.40***	***0.03322***
miR-31-5p	**1.31260**	**2.31418**	0.39	0.28403
miR-30e-3p	**0.11181**	**0.20683**	***0.37***	***0.00791***
miR-495-3p	0.03546	**0.06875**	***0.36***	***0.03439***
miR-181a-5p	**0.10553**	**0.21397**	0.34	0.20687
miR-149-5p	**0.11104**	**0.22733**	***0.34***	***0.00459***
miR-30a-3p	**0.09666**	**0.22827**	***0.29***	***0.00031***
miR-376a-3p	**0.19739**	**0.46711**	***0.29***	***0.03306***
miR-320a-3p	**0.44209**	**1.09114**	***0.28***	***0.00161***
miR-886-5p	0.02914	**0.07749**	***0.26***	***0.00084***
miR-574-3p	**0.85407**	**2.30085**	***0.26***	***0.00221***
miR-19a-3p	0.02921	**0.08026**	***0.25***	***0.00024***
miR-17-5p	**0.44209**	**1.24934**	***0.24***	***0.00083***
miR-744-5p	0.02632	**0.07704**	***0.24***	***0.00654***
miR-106a-5p	**0.40214**	**1.21658**	***0.23***	***0.00324***
miR-210-3p	**0.15885**	**0.51293**	***0.21***	***0.00012***
miR-191-5p	**1.58274**	**5.37961**	***0.20***	***0.00029***
miR-186-5p	0.03394	**0.11907**	***0.20***	***0.00063***
miR-301a-3p	0.02302	**0.08117**	***0.20***	***0.00020***
miR-484	**0.41440**	**1.48538**	***0.19***	***0.00017***
miR-19b-3p	**1.19949**	**4.57520**	***0.18***	***0.00005***
miR-376c-3p	**0.14218**	**0.59385**	***0.16***	***0.00029***
miR-146b-5p	0.03059	**0.14615**	***0.14***	***0.00078***
miR-134-5p	0.01779	**0.11469**	***0.11***	***0.00135***
miR-342-3p	0.04701	**0.31824**	***0.10***	***0.00012***
miR-335-5p	**0.09402**	**0.67776**	***0.10***	***0.00480***
miR-146a-5p	0.00334	**16.38347**	***0.00***	***0.00026***

Bold indicates miRNAs in hASC-EVs or hAMSC-EVs first quartile of expression, while bold and italics indicates differentially abundant miRNAs (ratio > 2 or < 0.5 with *P*-value < 0.05).

### Target and effect prediction of EV miRNAs on OA tissues

To frame the effect of hASC-EV and hAMSC-EV miRNAs in the OA setting, validated targets of the 90 most abundant molecules were compared with synovia and/or cartilage-dependent regulators of OA progression^[45] ^[Table 2]. This allowed obtaining the EV miRNA genetic weight for each targeted transcript. Regarding cytokines/chemokines, with the exception of anti-inflammatory IL4, all major OA-related inflammatory mediators, such as TNF, IFNG, IL1A/B, IL6 (and IL6-family related IL11), and IL18, are reported targets of EV miRNAs, with miR-125b-5p tipping the balance towards hASC-EVs for TNF and miR-191-5p towards hAMSC-EVs for IL1A. miR-146a-5p defined CXCL12, CCL5, CD40LG, and IL6 as hAMSC-EVs’ preferred targets, while miR-125b-5p framed a superior hASC-EVs’ regulation for EPO. Altogether, MSC-EVs appeared to interact with synovia-related inflammatory molecules. Regarding growth factors, TGFB1 emerged as the most heavily targeted transcript, with miR-146a-5p giving a higher weight to hAMSC-EVs. On the contrary, TGFB2 regulation is strongest for hASC-EVs, due to miR-21-5p. In addition, hASC-EVs preferentially modulate protective IGF1/2, with the latter being a target of miR-125b-5p, which also framed the higher regulation of ANGPT2. Among other growth factors, VEGF, EGF, CTGF, HGF, and FGF2 are similarly modulated by the two EV types. Eventually, both MSC-EVs’ miRNAs interact with several proteases secreted from both cartilage and synovia and involved in cartilage extracellular matrix degradation. The highest miRNA genetic weight emerged for MMP2/14/13/1 (in order of weight), with miR-125b-5p again tipping the balance towards hASC-EVs for MMP2/13. Of note, other proteases such as ADAM12/17, ADAMTS9, ST14, and plasminogen activators are also EV miRNA interactors. Interestingly, inhibitors of metalloproteases such as TIMPs laid among EV miRNA targets, although TIMP1/2 at lower levels with respect to TIMP3. miR-125b-5p framed hASC-EVs’ preference for APC, involved in promoting MMP activity.

**Table 2:** Soluble factors involved in OA pathological state and genetic weight of targeting EV miRNAs

	**hASC-EVs total genetic weight (main regulator)**	**hAMSC-EVs total genetic weight (main regulator)**	**Function**
**Cytokines/chemokines**			
IFNG	19.53% (miR-24-3p)	19.08% (miR-24-3p)	Pro-inflammatory
TNF	17.24% (miR-125b-5p)	4.30% (miR-125b-5p)	Pro-inflammatory
IL4	17.22% (miR-24-3p)	17.70% (miR-24-3p)	Anti-inflammatory
EPO	16.03% (miR-125b-3p)	2.67% (miR-125b-3p)	Upregulate Collagen, downregulate MMP-13
CXCL12	7.26% (miR-221-3p)	21.40% (miR-146a-5p)	Articular cartilage matrix degeneration
IL6	6.46% (miR-222-3p)	22.09% (miR-146a-5p)	Pro-inflammatory
IL1B	6.27% (miR-21-5p)	3.81% (miR-21-5p)	Pro-inflammatory
LIF	5.20% (miR-222-3p)	4.87% (miR-222-3p)	Cartilage erosion
IL11	3.06% (miR-30c-5p)	3.01% (miR-30c-5p)	Pro-inflammatory
IL1A	1.58% (miR-191-5p)	5.38% (miR-191-5p)	Pro-inflammatory
WNT1	1.54% (miR-34a-5p)	1.23% (miR-34a-5p)	Control Wnt signaling and aggravates OA pathology
CSF1	1.23% (miR-130a-3p)	0.76% (miR-152-3p)	Osteoclastogenesis enhancer, bone loss
CCL5	0.92% (miR-214-3p)	17.00% (miR-146a-5p)	Cartilage erosion
IL18	0.63% (miR-130a-3p)	0.25% (miR-130a-3p)	Pro-inflammatory
TNFSF11	0.33% (miR-106b-5p)	0.23% (miR-106b-5p)	Osteoclastogenesis enhancer, bone loss
CD40LG	0.00% (miR-146a-5p)	16.38% (miR-146a-5p)	Control the expression of inflammatory cytokines and MMP
**Growth factors**			
IGF2	19.95% (miR-125b-5p)	5.04% (miR-125b-5p)	Promote cartilage matrix levels
TGFB1	18.79% (miR-24-3p)	36.64% (miR-24-3p)	Cartilage homeostasis, high levels drive chondrocytes hypertrophy and synovial fibrosis
ANGPT2	18.70% (miR-125b-5p)	3.69% (miR-125b-5p)	Abnormal angiogenesis in OA
VEGFA	15.45% (miR-21-5p)	13.53% (miR-21-5p)	Promote OA process
TGFB2	7.97% (miR-21-5p)	4.89% (miR-21-5p)	Cartilage homeostasis, high levels released from joint tissue during OA development
CTGF	5.60% (miR-30c-5p)	4.47% (miR-30c-5p)	Promote osteophyte formation and ECM degradation
EGF	5.20% (miR-222-3p)	4.87% (miR-222-3p)	Promote chondrocyte catabolic activity
IGF1	3.78% (miR-29a-3p)	1.86% (miR-29a-3p)	Promote chondrocyte anabolic activity
BDNF	2.56% (miR-30a-5p)	2.13% (miR-132-3p)	Promote joint pain and inflammation
HGF	2.47% (miR-199a-3p)	1.40% (miR-199a-3p)	Cartilage homeostasis, promote osteophyte formation and osteoblast abnormal mineralization
FGF2	1.53% (miR-152-3p)	1.17% (miR-152-3p)	Promote catabolic and anti-anabolic effects in OA joints
BMP2	0.84% (miR-17-5p)	2.47% (miR-17-5p)	Promote cartilage regeneration
INHBB	0.69% (miR-34a-5p)	0.55% (miR-34a-5p)	TGFB superfamily, upregulatd in OA
KITLG	0.44% (miR-320a-3p)	1.09% (miR-320a-3p)	Promote synovial mast cell hyperplasia and inflammation
BMP6	0.16% (miR-22-3p)	0.12% (miR-22-3p)	Promote chondrocyte proliferation
TGFB3	0.09% (miR-29b-3p)	0.01% (miR-29b-3p)	Cartilage homeostasis, high levels released from joint tissue during OA development
PDGFC	0.09% (miR-29b-3p)	0.01% (miR-29b-3p)	Promote synovia MMP expression and angiogenesis
PDGFB	0.09% (miR-29b-3p)	0.01% (miR-29b-3p)	Promote subchondral bone angiogenesis
**Proteases and other**			
MMP2	31.92% (miR-125b-5p)	14.16% (miR-221-3p)	Metalloproteinase involved in ECM degradation
MMP13	20.69% (miR-125b-5p)	5.79% (miR-125b-3p)	Metalloproteinase involved in ECM degradation
MMP14	19.36% (miR-24-3p)	19.10% (miR-24-3p)	Metalloproteinase involved in ECM degradation
TIMP3	17.76% (miR-21-5p)	12.61% (miR-222-3p)	MMP inhibitor
APC	17.73% (miR-125b-5p)	4.24% (miR-125b-5p)	Promote MMP activity
MMP1	6.82% (miR-222-3p)	5.89% (miR-222-3p)	Metalloproteinase involved in ECM degradation
PLAT	6.27% (miR-21-5p)	3.81% (miR-21-5p)	ECM-degrading enzyme
PLAU	4.27% (miR-193b-3p)	2.37% (miR-193b-3p)	ECM-degrading enzyme
ADAM17	2.71% (miR-145-5p)	1.75% (miR-145-5p)	Metalloproteinase involved in ECM degradation
TIMP2	1.86% (miR-20a-5p)	2.96% (miR-20a-5p)	MMP inhibitor
ADAM12	1.60% (miR-29a-3p)	0.98% (miR-29a-3p)	Metalloproteinase involved in ECM degradation
ADAMTS9	0.98% (miR-29a-3p)	0.82% (miR-29a-3p)	Metalloproteinase involved in ECM degradation
MMP9	0.68% (miR-132-3p)	0.58% (miR-132-3p)	Metalloproteinase involved in ECM degradation
MMP15	0.53% (miR-29c-3p)	0.16% (miR-29c-3p)	Metalloproteinase involved in ECM degradation
ST14	0.35% (miR-27b-3p)	0.09% (miR-27b-3p)	Serine proteinase involved in cartilage destruction
TIMP1	0.11% (miR-181a-5p)	0.21% (miR-181a-5p)	MMP inhibitor
MMP3	0.06% (miR-93-5p)	0.10% (miR-93-5p)	Metalloproteinase involved in ECM degradation

Investigating the general picture given by miRNAs reported to regulate the overall homeostasis of cartilage and synovia at different levels, at first, we focused on miRNAs that directly impact OA cartilage pathogenesis^[46] ^[Table 3]. Eighteen protective and nine degenerative miRNAs were identified. hASC-EVs resulted enriched in miRNAs encompassing both categories (49% *vs*. 33% of EV genetic weight for protective, mainly due to miR-125b-5p, and 10% *vs*. 7% for destructive), with identical overall enrichment in OA-alleviating players, being the protective *vs*. destructive ratio 4.75 for both. Therefore, for cartilage, protective signals far exceeded damaging inputs. Notably, three miRNAs associated with overlapping roles in OA cartilage were present, and hAMSC-EV-specific miR-146a-5p had a strongly divergent expression. Regarding synovia, the definition of miRNA roles during OA progression is still in its infancy^[47]^. We identified two protecting and three damaging miRNAs [Table 3]. Albeit considering few players, no major differences between hASC-EVs and hAMSC-EVs could be detected, with again the preponderance for protecting players. Notably, as for cartilage, hAMSC-EV-specific miR-146a-5p was reported to have overlapping functions in synovia. To obtain further insight on synovia regulation, since many of the previously described cytokines/chemokines are mainly expressed by inflammatory cells, such as macrophages, we compared EV miRNAs with those reported to be involved in the macrophage M1 *vs*. M2 phenotype shift^[48]^, considering M1 inflammatory macrophages as a synovial OA landmark. Seven miRNAs involved in M2 and six involved in M1 phenotype regulation were identified [Table 3]. M1 miRNAs resulted more abundant in hASC-EVs (3.3% *vs*. 1.7%), while M2 miRNAs were more present in hAMSC-EVs (39.9% *vs*. 23.3%). Therefore, although the M2 to M1 ratio always resulted in favor of anti-inflammatory macrophages, hAMSC-EVs had a greater impact on M2 polarization (ratio of 23 *vs*. 7), mainly due to miR-146-5p, responsible for the M1 to M2 switch.

**Table 3:** miRNAs involved in OA pathological state at cartilage, synovium, and macrophage levels

	**hASC-EVs genetic weight**	**hAMSC-EVs genetic weight**	**Function**
**Cartilage protection**			
miR-30a-3p	0.10%	0.23%	Cartilage homeostasis
miR-210-3p	0.16%	0.51%	Anti-apoptotic promotes chondrocyte proliferation and ECM deposition
miR-149-5p	0.11%	0.23%	Anti-inflammatory
miR-193b-3p	4.27%	2.37%	Regulates inflammation by repressing TNF-α expression
miR-320a	0.44%	1.09%	Chondrocyte viability
miR-148a-3p	0.09%	0.05%	Promotes hyaline cartilage production
miR-199a-3p	0.90%	0.39%	Anti-catabolic
miR-30a-5p	0.65%	0.15%	Cartilage homeostasis
miR-26b-5p	0.14%	0.09%	Cartilage homeostasis
miR-222-3p	5.20%	4.87%	Controls cartilage degradation
miR-26a-5p	0.57%	0.30%	Cartilage homeostasis
miR-27b-3p	0.35%	0.09%	Anti-catabolic
miR-24-3p	17.22%	17.70%	Regulates chondrocyte senescence
miR-92a-3p	1.59%	1.04%	Anti-catabolic and increases collagen deposition
miR-130a-3p	0.63%	0.25%	Anti-inflammatory
miR-17-5p	0.44%	1.25%	Induces autophagy
miR-19a-3p	0.03%	0.08%	Promotes chondrocyte viability and migration
mR-125b-5p	16.03%	2.67%	Prevents aggrecan loss
**Cartilage destructive**			
miR-16-5p	0.44%	0.30%	Cartilage degradation
miR-34a-5p	0.69%	0.55%	Apoptosis
miR-30b-5p	2.34%	1.95%	Pro-apoptotic, ECM degradation
miR-181a-5p	0.11%	0.21%	Pro-inflammatory, procatabolic, cell death
miR-21-5p	6.27%	3.81%	Negatively regulates chondrogenesis
miR-138-5p	0.19%	0.02%	Promotes cartilage degradation
miR-23a-3p	0.10%	0.03%	Inhibits ECM synthesis
miR-483-5p	0.07%	0.10%	Stimulates chondrocyte hypertrophy, ECM degradation
miR-34a-3p	0.08%	0.05%	Apoptosis
**Cartilage overlapping**			
miR-145-5p	1.62%	1.02%	Chondrocyte proliferation *vs*. cartilage degradation
miR-221-3p	5.95%	2.68%	Prevents ECM degradation *vs*. pro-inflammatory
miR-146a-5p	0.00%	16.38%	Chondrocyte proliferation, anti-apoptosis *vs. *activator of early OA
**Synovia protection**			
miR-26a-5p	0.57%	0.30%	Anti-inflammatory
miR-29a-3p	0.98%	0.82%	Protects against excessive synovial remodeling
**Synovia destructive**			
miR-34a-3p	0.08%	0.05%	Enhance synovial inflammation
miR-181a-5p	0.11%	0.21%	Enhance synovial inflammation
miR-210-3p	0.16%	0.51%	Pro-fibrotic
**Synovia overlapping**			
miR-146a-5p	0.00%	16.38%	Anti-inflammatory *vs*. promoting oxidative stress
**Pro M1 macrophage**			
miR-145-5p	1.62%	1.02%	M1 promoting
miR-27b-3p	0.35%	0.09%	M1 promoting, M2 suppressing
miR-130a-3p	0.63%	0.25%	M1 promoting, M2 suppressing
miR-19a-3p	0.03%	0.08%	M2 suppressing
miR-26a-5p	0.57%	0.30%	M2 suppressing
miR-195-5p	0.12%	0.00%	M2 suppressing
**Pro M2 macrophage**			
miR-24-3p	17.22%	17.70%	M2 promoting, M1 suppressing
miR-146a-5p	0.00%	16.38%	M2 promoting, M1 suppressing
miR-146b-5p	0.03%	0.15%	M2 promoting, M1 suppressing
miR-181a-5p	0.11%	0.21%	M2 promoting, M1 suppressing
miR-34a-5p	0.69%	0.55%	M2 promoting
miR-222-3p	5.20%	4.87%	M2 promoting
miR-301a-3p	0.02%	0.08%	M2 promoting

### Identification of stable EV miRNA reference genes (RG)

To identify abundantly expressed and stable reference genes for future comparison analysis of novel miRNAs between hASC-EVs and hAMSC-EVs, four stability algorithms (Genorm, Normfinder, BestKeeper, and the comparative Delta Ct method) sifted the 64 miRNAs shared in both first quartiles (see Table 4 for the Top 10 and Supplementary Table 6 for the complete ranking). The most stable miRNAs were: (1) Genorm, miR-24-3p/127-3p (M-value of 0.00) and miR-34a-5p (0.27); (2) Normfinder, miR-34a-5p (SV of 0.25), miR-20a-5p (0.31), and miR-24-3p (0.34); (3) BestKeeper, miR-34a-5p (0.28), miR-30c-5p (0.28), and miR-99a-5p (0.28); and (4) Delta Ct, miR-34a-5p (0.87), miR-20a-5p (0.87), and miR-30c-5p (0.89). Notably, miR-27a-3p and miR-335-5p always resulted in the last two positions of the rankings. Eventually, the geometric mean (Geomean) of each putative RG weight across the four algorithms was calculated to identify a definitive hierarchy, considering the RG with the final lowest value as the most stable. miR-34a-5p clearly ranked best (Geomean of 1.73), while miR-335-5p laid in the last position (64).

**Table 4:** Top 10 hASC-EVs and hAMSC-EVs first quartile shared miRNAs’ stability ranking

	**Genorm M-value**	**Normfinder SV**	**BestKeeper SD**	**Delta CT SD**	**Geomean**
miR-34a-5p	0.27	0.25	0.28	0.87	1.73
miR-24-3p	0.00	0.34	0.44	0.89	3.66
miR-20a-5p	0.36	0.31	0.50	0.87	4.52
miR-127-3p	0.00	0.34	0.44	0.89	4.53
miR-30c-5p	0.57	0.35	0.28	0.89	5.59
miR-99a-5p	0.46	0.37	0.28	0.90	6.09
miR-28-3p	0.41	0.35	0.44	0.90	7.65
miR-331-3p	0.44	0.45	0.33	0.94	7.90
miR-365a-3p	0.64	0.58	0.28	0.98	8.60
miR-106b-5p	0.58	0.59	0.28	1.00	10.03

## DISCUSSION

In this report, EVs and embedded miRNAs from adipose- and amniotic membrane-derived MSCs, characterized with the same technical workflow and platform, were compared. In the frame of a shared overall molecular signature targeting several OA-related factors and processes, with few miRNAs tipping the balance, both hAMSCs and hASCs were able to release EVs with pro-M2 macrophage-polarizing and cartilage-protective cargo.

When selecting an MSC type to be envisioned as a therapeutic agent, several characteristics have to be taken into consideration. First, like the ease of accessibility and absence or low risk of morbidity. In this perspective, the collection of both adipose tissue and amniotic membrane, usually discarded in large amounts as waste material, has an advantage over bone marrow harvesting that requires a dedicated and often uncomfortable procedure to obtain reduced volumes of starting material. Second, amniotic membrane and adipose tissue have a high MSC content, in the range of 10^5^-10^6^ MSC per gram^[8]^, and therefore largely more abundant than bone marrow^[10]^. Third, hAMSCs and hASCs showed superior immune regulation over bone marrow MSCs^[49,50]^. This has led to the creation of more and more hASCs/hASC products^[51]^, and more recently hAMSCs and amniotic membrane-derived tissues^[52]^ have gained attention in musculoskeletal regenerative medicine. Since the therapeutic potential of MSCs is ascribed to their secreted factors and EVs, a thorough characterization of these compartments is mandatory to envision the most effective source, especially for EVs that were recently proposed as standalone and cell-free medicinal agents for several pathologies^[53]^, including OA^[54]^. In this frame, an often-underestimated issue is the EVs’ secretory capacity. This is crucial for both expanded MSCs and MSC-containing products, such as SVF or ASA, as well as for purified EVs. In the first case, a MSC type or source having a higher release of therapeutic and active EVs might have a stronger healing effect on target tissues. In addition, when purified EVs are produced as good manufacturing practice (GMP) products, a higher secretory ability will reduce the culture surface area per unit, the overall culturing time, and, by consequence, the cost of the production process for both the industry and national health system. In this study, we demonstrated that hAMSCs may release significantly more EVs per cell, which, in combination with a higher number of MSCs per gram of fresh tissues, suggests the secretion/collection of a greater number of hAMSC-EVs, both at the point-of-care and after *in vitro* expansion. We acknowledge that the different culture media used for hASCs and hAMSCs might have influenced cell physiology and secretory capacity, both the number of cells and the molecular content. Moreover, any *in vitro* condition is presumably far from the one those cells encounter in their therapeutic site (e.g., synovial fluid for OA treatment), possibly making the herein presented results not completely stackable with MSC behavior in cell-based therapeutic applications. Given these premises, we preferred cultivating cells in their specific and recognized media, as reported in the literature for *in vitro* expansion, considering media-related influence not a pitfall but a distinctive feature defining the fingerprint of currently used hASCs/hAMSCs and their secretomes/EVs. In this view, it will be crucial to understand whether therapeutic attributes of different MSCs and their EVs are due to the environmental conditions (site of administration, media, confluence, substrate, *etc*.), and whether by adjusting these conditions it is possible to optimize EVs for the target therapeutic application, as suggested in the last years for MSCs in general^[55]^ and as demonstrated by our group for hASC-EV miRNAs after inflammatory priming^[56]^. Moreover, further characterization is needed to understand whether media-modulated MSC therapeutic attributes might be stable for some generations, making this option more attractive for cell therapies, or molecular changes are quickly transient as demonstrated for umbilical cord blood MSC intracellular miRNAs^[57]^, suggesting media-based priming more indicated for cell-free therapies relying on secreted factors and EVs.

The second crucial issue defining cell expendability is the specific activity within a defined pathology. Many EV functions were ascribed to their nucleic acid content, especially miRNAs^[58,59]^. This was also demonstrated for MSC-EVs^[60]^. Therefore, given a similar number of EVs, the differential miRNA portfolio or its modulation may greatly impact the therapeutic message, taking also into account the biologically relevant miRNA concentration, biochemical functionality, and potential to elicit a timely response^[43,61]^. Moreover, although the amount of a regulatory molecule might allow a reliable prediction of its effects, since each miRNA may target several mRNA molecules, each present at a variable level of abundance and in different cellular districts possibly hindering its availability for interaction, it is not always possible to directly predict the impact of single or few miRNAs on the target cell or tissue. Thus, all these factors were considered for the EV-miRNA analysis herein proposed, avoiding discussion of a few players with high expression but relying on those laying in the first quartile of expression and covering > 95% of the EV genetic message that, as a whole, resulted shared between hASC-EVs and hAMSC-EVs, since the vast majority of molecules are mutual. Nevertheless, the differential expression of a few players allowed sharply clustering the two tissue sources, regardless of donor variability. This is important since a conserved genetic message that goes beyond donor-dependent fluctuations is mandatory to predict constant efficacy on a specific disease. In the frame of OA, several abundant EV miRNAs targeted single factors^[45] ^[Table 2] and fell within the general players^[46] ^[Table 3] involved in disease pathogenesis. In the first category, many pro-inflammatory and synovia-specific cytokines^[62]^ (TNF, IL6, IL1B, IL1A, IFNG, and IL18) are targeted. In particular, many of these key OA inflammatory mediators are secreted by synovia resident immune cells, including macrophages^[45]^. Intriguingly, together with the influence on macrophage secretion at a single factor level, where hASC-EVs had a greater impact on TNF due to miR-125b-5p and hAMSC-EVs on IL1A due to miR-191-5p and IL6 due to miR-146a-5p, miRNAs also had a profound influence on macrophage polarization. The macrophage anti-inflammatory phenotype was highly supported due to the presence of both pro-M2 and anti-M1 miRNAs, especially in hAMSC-EVs where the M2:M1 miRNA ratio was 23.5:7.1 in hASC-EVs. The discriminating factor was hAMSC-EVs enriched miR-146a-5p that in several studies reduced M1 and promoted M2 macrophage polarization^[63-65]^. Consistently, the only study comparing hASCs and hAMSCs activity on macrophages reported a higher capacity for M2 polarization in favor of hAMSCs^[20]^. Together with inflammatory factors, other OA-related and synovia specific cytokines are preferential hAMSC-EVs miRNA targeted. Among them, CXCL12 can induce chondrocyte death during the OA process^[66]^ and its levels are increased in OA synovial fluid^[67]^ and CCL5, one of the mediators most significantly elevated in OA synovial fluid^[68]^, takes part in cartilage catabolism^[69]^. Conversely, EPO, which upregulates collagen expression while reducing MMP13, is more targeted by hASC-EVs due to miR-125b-5p. hAMSC-EVs’ superior OA protective features also emerged for growth factors [Table 2]. In fact, in a shared scenario of OA driving factors targeting, such as VEGFA, CTGF, EGF, BDNF, and HGF, hAMSC-EVs more actively spotted TGFB1, which, at high and constant levels, as in OA patients, changes from a factor that blocks to a factor that facilitates chondrocyte hypertrophy, together with synovial fibrosis and osteophytes^[70]^. On the contrary, hASC-EVs more likely target IGF1 and especially IGF2, both having anabolic effects on cartilage, thus further reducing their bioavailability that is already suppressed in OA synovial fluid by the formation of high molecular weight complexes with their specific binding proteins^[71]^. Eventually, several molecules involved in cartilage ECM degradation, including MMPs and ADAM/ADAMTS^[72]^, are highly targeted by EV miRNAs with respect to few inhibitors, especially TIMP3. In this case, hASC-EVs resulted preferential modulators, due to miR-125b-5p targeting of MMP2/13 and APC, involved in MMP2/13 activation^[73]^. A clear influence on the reduction of OA phenotype also emerged when comparing EV miRNAs with those involved in either disease or healing pathways, rather than sifting only single factors [Table 3]. In a scenario of general balance between hASC-EV and hAMSC-EV miRNAs, with identical overall protective *vs*. destructive ratio in favor or healing mechanisms, miR-125b-5p discriminated the two EV types, allowing hASC-EVs to have almost 50% of their miRNA genetic message involved in protective roles. The other EV-discriminating miRNA, hAMSC-related miR-146a-5p, was reported to have a dual function, being both an activator in early OA, enhancing cartilage destruction^[46]^, and a repressor in late OA, by promoting chondrocyte proliferation and anti-apoptotic mechanisms through inhibition of NF-κB pathway^[65]^. And miR-146a-5p was also described as having a dual role in the synovium^[47]^, being both anti-inflammatory and an inducer of oxidative stress. Nevertheless, miRNAs’ role in synovia is still underestimated, and available data do not allow a deep evaluation of EVs’ impact on the tissue. An example is miR-125b-5p, the expression of which increases with OA severity and inhibits synovial cell proliferation by promoting apoptosis, being therefore considered a pathological miRNA^[74]^. Nevertheless, miR-125b-5p upregulation might also be an attempt to attenuate synovial hyperplasia and fibrosis in an effort to maintain normal synovial function rather than contributing to pathologic OA disease progression. Therefore, at present, a clear role for miR-125b-5p is unclear, and we could not include this miRNA in the analyzed categories.

We are aware that one of the main limitations of this study, together with the restricted description of miRNA function for several tissues, was the limited number of tested miRNAs. We preferred to focus on well-described molecules, recognizing that several new miRNAs are discovered on an almost daily basis. For this reason, a reliable normalization strategy for future evaluations is mandatory. The most sensitive quantification approach is the miRNA global mean expression, relying on obtaining a large portfolio of the miRNome^[75]^. Accordingly, we used this method for whole dataset comparison. Nevertheless, preparation of a large volume of EVs and an expensive high throughput search would be needed each time different samples are compared, making the process unsustainable for both research applications, studying single or few miRNAs, and clinical trials with GMP batches. This is in fact another major issue that has also been debated recently for EVs from umbilical cord-derived MSCs, in order to facilitate translational research^[76]^. In this context, to the best of our knowledge, studies comparing hASCs and hAMSCs do not suggest miRNA reference genes in general, or for EVs in particular. Therefore, we wanted to obtain reference genes behaving similarly to the global mean approach, and bioinformatics was applied to normalized data in place of raw values. With these premises, miR-34a-5p clearly resulted the best candidate. Interestingly, miR-16-5p, which resulted the most stable according to averaged C_RT_ values (hASC-EVs *vs*. hAMSC-EVs ratio of 1), ranked 26th, suggesting that overall stability might mask fluctuations at the donor level.

In conclusion, both EV types possessed chondro-protective and pro-M2 macrophage features due to several embedded miRNAs. These results provide, at least for the EV miRNA role, the molecular basis for the significant improvement driven by hASC- and hAMSC-based products in terms of inflammation reduction and joint function observed in pivotal clinical studies. Molecular data suggest stronger commitment in anti-inflammatory macrophage modulation for hAMSC-EVs and a less defined picture for the definition of the best EV type in cartilage protection, where harmful growth factors are the preferential hAMSC-EV target, whereas ECM was more protected by hASC-EVs’ inhibitory activity on proteases and the presence of miR-125b-5p. The observed *in vitro* increased chondro-protection and M1:M2 synovial macrophage ratio reduction for hAMSCs with respect to hASCs^[20]^ might be due to the highest EV release of hAMSCs that can overcome the similar chondro-protective ability. Future clinical studies to address the issue regarding EVs dose will be necessary, especially in the frame of GMP clinical products. Regarding MSC-enriched, tissue-based, one-step procedures, the feasibility of tissue harvesting, the ease of bedside treatment, and the allogeneic *vs*. autologous issue might drive adipose or amniotic membrane tissue selection, both relying on active OA healing and counteracting MSC populations.

## DECLARATIONS

### Copyright

© The Author(s) 2021.
