# Supplementary material for: Comparison of miRNA cargo in human adipose-tissue vs. amniotic-membrane derived mesenchymal stromal cells extracellular vesicles for osteoarthritis treatment
Source: Extracell Vesicles Circ Nucl Acids. 2021 Aug 3;2(3):202–21. doi: 10.20517/evcna.2021.11 (PMC11648501; doi:10.20517/evcna.2021.11)
Supplement: Supplementary file 1 [file evcna-2-3-202-SupplementaryMaterials.pdf]

Supplementary Table 1. EV-miRNA Normalized C_{RT} values

miRBase ID	hASC1-EVs	hASC2-EVs	hASC3-EVs	hAMSC1-EVs	hAMSC2-EVs	hAMSC3-EVs
let-7a-5p	11.41	11.97	14.52	14.26	14.63	14.83
let-7c-5p	12.90	12.04	13.11	17.31	17.13	17.35
let-7d-5p	15.65	16.12	18.04	15.56	16.60	16.89
let-7e-3p	24.50	25.97	26.05	27.31	27.34	26.63
let-7g-5p	14.89	14.72	15.80	14.16	15.21	14.63
miR-100-3p	23.58	25.07	26.23	23.67	24.46	25.16
miR-100-5p	7.94	8.26	8.92	7.92	8.95	8.56
miR-101-3p	18.68	19.08	19.66	19.92	20.30	20.15
miR-103a-3p	13.91	15.27	16.41	13.84	15.01	15.03
miR-106a-5p	11.40	12.12	11.09	9.14	9.65	9.41
miR-106b-5p	11.76	11.95	11.70	11.59	12.25	11.65
miR-10a-3p	21.83	22.08	21.05	21.77	22.74	22.40
miR-10a-5p	11.85	11.73	13.17	10.73	12.53	12.49
miR-10b-5p	13.96	14.36	16.05	17.39	20.91	19.06
miR-1180-3p	20.22	18.82	18.64	18.97	19.30	19.17
miR-1197	21.27	24.00	21.71	24.77	24.96	25.65
miR-1226-5p	20.07	19.23	18.22	20.54	20.08	19.67
miR-1227-3p	19.87	19.37	17.82	21.82	20.55	19.87
miR-1244	22.66	25.05	23.27	23.68	23.04	22.21
miR-1254	21.88	23.13	21.19	22.30	22.91	21.82
miR-1255b-5p	19.89	20.76	18.55	21.89	20.27	19.94
miR-125a-3p	21.96	22.83	22.70	21.18	20.85	20.63
miR-125a-5p	14.79	14.81	15.29	12.26	14.44	14.47
miR-125b-1-3p	17.57	17.77	17.15	19.04	18.52	18.92
miR-125b-5p	5.86	6.14	6.66	8.55	7.87	8.38
miR-1260a	13.60	12.08	12.70	19.45	17.22	17.16
miR-1262	23.87	25.67	25.24	25.78	23.50	23.80
miR-126-3p	18.48	20.74	21.42	15.37	18.90	18.25
miR-126-5p	20.63	22.08	22.10	19.88	20.66	20.93
miR-1270	22.93	22.73	22.17	22.76	22.45	22.44
miR-1271-5p	19.39	18.53	17.15	18.31	18.48	18.84
miR-127-3p	11.45	10.35	11.10	10.44	10.00	10.35
miR-127-5p	23.53	24.89	23.53	23.79	22.77	23.81
miR-1285-3p	19.57	20.98	17.96	21.43	21.56	21.33
miR-128a-3p	17.19	17.09	16.92	18.23	16.80	17.64
miR-1290	18.87	17.80	17.79	19.79	18.38	18.81
miR-1291	14.17	14.06	14.27	15.33	16.91	16.58
miR-1303	18.66	19.22	17.91	19.81	19.25	20.09
miR-130a-3p	10.75	10.95	10.95	11.39	11.22	12.44
miR-130b-3p	14.02	13.94	13.75	13.60	12.77	13.56
miR-132-3p	10.80	11.23	11.79	11.41	10.24	10.20
miR-133a-3p	19.82	20.40	19.67	18.34	18.66	17.11
miR-134-5p	16.75	15.72	15.63	12.46	12.85	13.11
miR-135b-5p	22.08	23.79	23.13	17.33	19.47	19.23
miR-136-3p	19.30	18.48	18.31	17.20	16.52	16.91
miR-137-3p	14.75	16.13	16.08	14.22	16.68	16.33
miR-138-5p	11.88	12.85	13.11	15.12	14.38	15.91
miR-139-5p	18.91	18.59	19.47	19.07	17.74	17.69
miR-140-3p	18.02	18.55	18.94	19.01	19.30	19.13
miR-140-5p	13.84	14.82	15.21	13.50	15.09	14.39
miR-142-3p	22.88	22.12	23.92	20.13	20.31	20.54
miR-143-3p	12.97	13.04	14.18	13.56	14.59	13.75
miR-145-3p	20.28	19.82	20.67	22.50	21.83	23.62

miR-145-5p	9.33	9.12	10.12	10.52	8.95	9.49
miR-146a-5p	16.75	20.34	18.25	5.89	5.53	5.53
miR-146b-3p	21.13	21.56	21.55	21.47	22.25	21.63
miR-146b-5p	14.87	15.19	15.70	12.49	12.12	12.76
miR-148a-3p	13.84	13.83	13.65	15.31	13.31	13.01
miR-148b-3p	17.46	17.90	18.11	17.38	17.06	17.40
miR-148b-5p	21.65	22.07	21.18	21.66	23.31	23.54
miR-149-5p	13.65	13.04	13.49	11.51	11.74	12.21
miR-150-5p	18.82	21.15	19.61	20.12	19.46	19.30
miR-151a-3p	14.92	16.12	15.18	14.35	15.32	15.00
miR-151a-5p	18.24	18.99	19.42	18.90	19.83	19.39
miR-152-3p	11.08	10.97	11.41	10.84	11.04	10.84
miR-154-3p	20.70	20.59	20.78	21.23	21.67	23.61
miR-154-5p	18.26	18.01	18.55	19.77	17.67	18.51
miR-155-5p	17.53	18.50	18.54	12.28	15.52	14.40
miR-15a-3p	21.37	23.25	21.37	23.60	22.86	23.40
miR-15a-5p	16.66	17.73	18.47	17.44	19.29	19.04
miR-16-1-3p	22.25	22.70	22.18	23.05	22.57	22.59
miR-16-5p	11.24	11.34	11.62	10.87	12.28	11.06
miR-17-5p	11.30	11.79	11.11	9.17	9.46	9.46
miR-181a-2-3p	19.01	20.23	20.97	17.64	18.49	17.30
miR-181a-3p	19.50	20.85	20.56	17.46	19.28	19.50
miR-181a-5p	12.11	13.28	15.01	10.72	12.66	12.35
miR-181c-5p	17.98	19.57	20.15	17.59	19.24	19.39
miR-184	17.72	17.63	19.10	22.71	21.26	21.61
miR-185-5p	17.55	17.49	17.19	17.14	17.27	16.69
miR-186-5p	14.81	15.31	15.19	12.64	13.12	12.50
miR-18a-3p	23.54	22.86	22.16	22.36	22.30	22.94
miR-18a-5p	18.17	18.07	17.17	16.90	17.29	17.60
miR-190a-5p	17.89	18.15	20.03	21.33	23.38	23.26
miR-190b-5p	22.77	23.43	23.17	25.00	24.97	23.23
miR-191-3p	24.13	24.92	23.69	21.94	21.97	21.07
miR-191-5p	9.56	9.52	9.60	7.43	7.47	6.87
miR-192-5p	16.34	17.09	16.88	15.53	15.52	15.20
miR-193a-3p	24.06	25.15	22.74	20.31	22.06	21.90
miR-193a-5p	12.83	12.90	12.86	12.84	11.59	11.89
miR-193b-3p	8.15	8.10	8.13	9.62	8.03	7.68
miR-193b-5p	16.94	16.86	16.69	19.72	17.52	17.91
miR-194-5p	18.42	20.40	20.76	17.64	20.21	19.69
miR-195-5p	13.15	13.01	13.56	16.96	18.02	17.05
miR-196b-5p	19.14	19.24	21.07	22.38	23.75	25.72
miR-197-3p	11.77	11.10	11.59	12.52	10.48	11.05
miR-198	20.43	19.49	22.05	23.61	23.28	23.28
miR-199a-3p	10.41	9.98	10.74	10.65	11.69	10.83
miR-199a-5p	15.09	15.15	15.83	16.97	17.21	18.16
miR-199b-5p	15.57	14.71	15.13	18.25	18.17	18.20
miR-19a-3p	15.52	15.42	15.02	13.42	13.29	13.26
miR-19b-1-5p	22.18	22.29	21.45	20.25	20.60	20.75
miR-19b-3p	10.12	10.05	9.71	7.53	7.43	7.51
miR-203a-3p	18.85	19.71	19.21	17.94	18.80	18.06
miR-204-5p	14.84	14.04	16.55	19.15	19.36	18.88
miR-20a-3p	22.33	23.06	23.23	21.89	24.31	22.93
miR-20a-5p	9.65	9.97	9.46	8.91	8.97	8.98
miR-20b-5p	18.69	19.89	17.61	16.14	16.66	16.20
miR-210-3p	12.87	12.92	12.84	10.86	10.71	10.36
miR-212-3p	14.88	15.55	15.76	15.15	14.68	14.33

miR-214-3p	10.37	10.09	10.56	11.02	10.30	9.78
miR-214-5p	17.39	17.89	17.52	18.56	18.32	18.44
miR-21-5p	7.18	7.52	8.02	7.58	8.10	7.59
miR-218-2-3p	24.66	22.73	23.06	27.70	26.28	25.45
miR-218-5p	11.01	10.66	10.15	11.17	11.42	11.29
miR-219a-5p	23.43	23.00	22.72	25.40	22.37	23.33
miR-221-3p	7.87	8.03	7.12	8.21	7.98	8.60
miR-221-5p	20.79	21.83	22.10	23.26	26.68	26.44
miR-222-3p	7.65	7.74	8.14	6.91	7.24	8.05
miR-222-5p	16.57	16.38	15.58	17.79	18.28	17.74
miR-223-3p	19.82	20.65	20.86	21.43	20.39	19.61
miR-22-3p	12.75	12.87	13.03	12.59	12.56	13.10
miR-224-5p	11.46	10.76	11.09	13.12	13.08	11.87
miR-22-5p	15.59	15.13	15.37	16.79	17.03	17.25
miR-23a-3p	13.33	13.40	13.75	15.63	14.53	14.33
miR-23a-5p	21.74	22.46	21.36	24.49	23.48	23.54
miR-24-2-5p	16.89	17.31	17.09	18.78	18.99	19.78
miR-24-3p	6.00	5.98	6.37	5.44	5.55	5.63
miR-25-3p	12.84	12.78	12.09	13.49	12.98	13.46
miR-26a-1-3p	27.02	26.31	27.81	26.49	27.70	26.03
miR-26a-2-3p	26.06	27.52	25.00	27.10	26.23	28.58
miR-26a-5p	10.68	10.61	11.83	10.41	12.08	11.84
miR-26b-3p	24.98	25.12	24.28	22.68	23.84	23.63
miR-26b-5p	12.81	12.71	13.62	12.88	13.28	13.48
miR-27a-3p	10.30	9.97	10.51	12.07	12.50	13.65
miR-27a-5p	19.81	19.46	19.18	20.15	18.84	17.84
miR-27b-3p	11.96	11.26	11.93	12.56	12.81	13.95
miR-27b-5p	20.64	19.18	20.64	21.22	21.56	21.95
miR-28-3p	13.52	13.41	13.54	12.39	12.70	13.04
miR-28-5p	12.62	13.04	13.63	12.46	14.10	14.42
miR-296-3p	20.97	20.65	20.37	20.24	19.51	20.28
miR-296-5p	13.20	12.47	12.81	13.39	11.96	12.99
miR-299-5p	20.12	19.63	20.26	19.39	20.56	20.04
miR-29a-3p	9.86	10.48	10.41	8.85	10.73	10.32
miR-29a-5p	14.61	14.22	14.14	17.39	16.97	18.18
miR-29b-3p	12.88	13.98	14.23	14.63	16.21	16.56
miR-29c-3p	10.87	11.62	11.77	11.72	12.89	12.65
miR-29c-5p	23.54	25.72	24.18	24.89	25.70	24.64
miR-301a-3p	15.72	15.64	15.63	13.18	13.08	13.66
miR-301b-3p	19.96	20.14	20.35	17.48	17.80	18.60
miR-30a-3p	13.63	13.43	13.72	11.99	11.57	11.89
miR-30a-5p	10.68	10.85	11.00	12.18	11.99	13.17
miR-30b-5p	8.86	8.91	9.21	9.20	8.32	8.63
miR-30c-5p	8.47	8.51	8.85	8.29	7.75	8.25
miR-30d-3p	13.41	13.18	13.47	23.20	22.12	24.63
miR-30d-5p	21.88	22.31	21.59	14.72	15.55	16.36
miR-30e-3p	13.49	13.10	13.56	12.41	11.54	11.92
miR-31-3p	11.46	12.04	11.89	11.48	12.32	12.64
miR-31-5p	8.71	10.00	10.78	6.67	9.11	9.64
miR-320a-3p	11.27	11.30	11.63	9.98	9.30	9.39
miR-320b	16.50	16.27	16.05	17.21	16.09	16.86
miR-323a-3p	18.78	17.55	17.15	14.46	14.59	14.12
miR-324-3p	16.52	16.83	16.88	13.90	15.63	15.59
miR-324-5p	13.47	14.04	14.48	13.46	14.22	14.52
miR-32-5p	22.82	23.11	22.58	24.06	26.49	23.16
miR-328-3p	11.16	10.79	11.17	12.04	9.89	10.21

miR-329-3p	20.40	19.12	19.61	19.20	17.50	19.10
miR-330-3p	16.88	17.71	17.40	16.55	15.24	15.50
miR-331-3p	11.72	11.70	12.74	10.35	11.70	11.83
miR-335-3p	18.32	18.95	19.21	14.19	16.08	15.07
miR-335-5p	12.78	14.07	14.05	10.45	10.85	9.44
miR-337-3p	21.35	20.58	20.04	21.71	21.57	21.73
miR-337-5p	18.88	17.95	17.90	16.88	17.14	17.55
miR-339-3p	18.29	18.06	18.16	15.37	16.50	16.50
miR-33a-5p	22.53	23.10	23.04	23.06	23.08	22.92
miR-340-3p	21.51	23.08	22.93	22.77	23.06	23.99
miR-340-5p	20.84	20.61	21.44	19.79	21.86	20.44
miR-342-3p	14.83	14.32	14.75	11.55	11.43	11.03
miR-345-5p	16.95	16.97	17.38	13.88	14.20	14.92
miR-34a-3p	14.01	13.66	13.78	13.66	14.20	13.90
miR-34a-5p	10.72	10.41	11.14	10.36	10.56	10.69
miR-34b-3p	15.19	15.04	14.61	16.22	15.61	15.98
miR-34b-5p	18.79	18.76	18.70	21.02	21.49	22.93
miR-34c-5p	14.76	14.42	14.87	15.31	15.86	15.91
miR-361-5p	13.85	13.63	13.65	14.21	13.64	14.15
miR-362-3p	20.45	20.11	19.89	20.37	20.40	20.75
miR-362-5p	18.88	19.98	20.82	19.02	19.41	18.86
miR-365a-3p	12.28	12.92	12.88	13.12	12.71	12.31
miR-369-3p	20.44	18.79	19.77	20.70	19.27	20.07
miR-369-5p	23.46	23.98	23.17	22.37	22.07	22.31
miR-372-3p	21.32	23.05	21.16	17.77	15.21	18.62
miR-373-3p	21.65	20.88	20.45	22.10	19.90	20.03
miR-374a-5p	13.87	14.27	15.31	14.05	15.60	14.68
miR-374b-5p	14.26	15.07	15.67	14.26	16.24	15.72
miR-375-3p	22.60	21.31	22.12	24.36	22.39	25.12
miR-376a-3p	13.41	12.15	12.13	11.45	10.21	10.69
miR-376b-3p	21.12	19.81	20.39	20.37	19.01	19.63
miR-376c-3p	13.42	12.86	12.83	10.63	10.40	10.28
miR-377-5p	22.07	22.23	21.99	22.74	22.16	23.85
miR-378-3p	20.70	20.11	19.10	21.07	20.66	21.55
miR-378a-5p	21.87	23.06	21.37	24.22	23.98	24.47
miR-379-5p	15.88	16.02	17.38	14.41	16.65	16.00
miR-380-3p	23.16	20.92	21.57	22.26	19.94	20.29
miR-380-5p	21.87	20.12	19.88	21.76	21.31	21.63
miR-381-3p	19.62	18.72	18.33	19.84	18.74	19.37
miR-382-5p	11.83	10.22	10.89	10.75	9.80	10.21
miR-409-3p	12.70	11.45	12.01	12.35	10.59	9.89
miR-409-5p	19.57	19.67	20.56	19.47	19.10	19.66
miR-410-3p	15.68	15.15	15.69	14.24	15.05	13.78
miR-411-3p	22.74	22.38	22.57	21.48	22.75	22.39
miR-411-5p	14.30	14.56	15.68	13.30	15.17	14.08
miR-422a	23.11	23.24	23.76	23.04	23.07	22.62
miR-423-5p	15.65	15.21	15.17	16.48	14.41	14.82
miR-424-3p	15.89	15.80	15.72	17.16	15.69	16.00
miR-424-5p	14.64	14.57	16.05	16.06	15.47	16.71
miR-425-3p	21.02	21.07	20.77	20.06	19.53	18.35
miR-432-3p	19.19	17.22	17.80	17.08	16.04	15.16
miR-433-3p	18.82	17.89	18.57	17.42	16.56	16.69
miR-449b-5p	25.26	25.71	24.69	22.70	23.28	24.10
miR-450a-5p	21.02	21.18	21.94	21.64	21.71	22.06
miR-451a	21.01	21.15	21.01	24.86	22.32	22.65
miR-452-5p	16.81	16.49	17.10	16.44	18.44	17.32

miR-454-5p	26.12	26.68	26.35	23.77	24.57	23.75
miR-455-3p	16.05	17.27	18.01	15.90	15.81	16.65
miR-455-5p	17.50	18.51	18.07	16.44	16.25	16.67
miR-483-5	15.05	13.08	13.76	14.29	12.55	12.00
miR-484	11.41	11.47	11.60	9.41	8.84	9.09
miR-485-3p	18.83	17.24	18.03	19.86	17.40	18.30
miR-485-5p	17.53	17.31	18.26	18.05	16.41	16.65
miR-487a-3p	21.53	21.19	20.85	20.46	19.46	20.73
miR-487b-3p	18.82	17.75	18.01	16.63	16.56	16.59
miR-490-3p	ND	ND	ND	19.56	19.37	18.56
miR-491-5p	16.69	17.39	17.94	16.99	17.39	17.57
miR-493-3p	19.81	19.13	19.74	16.66	17.15	16.07
miR-494-3p	16.02	15.60	15.80	14.45	14.27	13.67
miR-495-3p	15.78	14.82	14.52	14.01	13.04	13.59
miR-500a-5p	17.83	18.87	19.16	18.85	20.03	20.00
miR-502-3p	18.74	18.85	18.29	19.34	19.72	19.60
miR-502-5p	18.87	19.53	20.12	19.28	20.22	19.71
miR-503-5p	16.80	16.03	16.31	15.41	15.22	14.85
miR-505-3p	18.44	19.08	18.16	19.81	18.92	19.68
miR-505-5p	20.73	20.89	21.07	21.36	22.43	22.41
miR-532-3p	15.13	15.85	15.94	13.79	14.88	14.35
miR-532-5p	13.59	14.02	13.96	12.53	12.93	12.62
miR-539-5p	18.45	17.88	18.38	15.10	16.13	15.02
miR-542-3p	18.53	18.73	19.45	19.02	20.91	20.85
miR-542-5p	20.51	21.08	20.96	18.37	20.38	19.65
miR-543	18.48	16.96	16.81	18.51	16.87	17.68
miR-544Aa	28.88	26.03	27.27	26.27	26.36	27.90
miR-545-3p	23.87	24.71	22.71	23.41	24.82	23.67
miR-548am-5p	20.32	20.87	19.89	22.32	21.01	21.42
miR-548b-5	20.04	20.51	19.31	21.51	20.69	20.60
miR-548d-3p	22.78	23.08	23.61	25.38	27.24	25.73
miR-548d-5	20.42	21.27	20.23	22.86	21.13	21.42
miR-570-3p	25.28	26.01	24.95	25.76	24.88	26.36
miR-572	19.17	18.80	18.51	21.45	21.10	21.25
miR-574-3p	10.60	10.22	10.53	8.98	8.14	8.32
miR-576-3p	19.21	19.38	19.15	19.76	18.79	19.12
miR-579-3p	20.89	20.12	19.74	21.39	20.83	22.30
miR-589-3p	19.81	19.36	19.18	20.12	19.56	19.21
miR-590-3p	21.77	21.10	21.88	20.53	22.10	20.77
miR-590-5p	16.33	16.03	16.02	14.75	14.94	14.66
miR-597-5p	19.51	19.40	19.00	19.87	19.25	20.25
miR-598-3p	20.91	22.83	22.08	18.53	19.07	18.57
miR-601	16.83	13.34	16.10	23.72	22.54	22.47
miR-605-5p	19.68	20.07	18.85	23.50	21.56	21.82
miR-616-3p	20.84	21.13	20.16	20.11	19.77	19.71
miR-616-5p	21.24	20.95	20.05	21.75	21.90	22.92
miR-622	21.02	23.72	23.89	24.44	23.27	24.95
miR-625-3p	18.89	18.14	18.03	19.32	18.59	17.66
miR-625-5p	23.03	22.39	22.76	20.93	22.50	21.66
miR-628-3p	22.97	22.57	22.40	24.68	25.03	23.50
miR-628-5p	21.55	21.72	22.08	21.14	22.43	21.68
miR-629-3p	19.50	19.15	18.17	18.42	17.14	16.99
miR-629-5p	23.00	23.07	23.12	21.88	21.09	21.85
miR-638	17.80	17.13	17.14	21.58	19.34	19.94
miR-642a-5p	19.28	19.16	18.89	18.18	18.16	17.67
miR-652-3p	18.73	18.96	19.58	16.86	18.55	18.65

miR-654-3p	21.78	21.08	20.45	20.78	19.26	19.49
miR-654-5p	15.80	14.75	14.47	13.88	13.61	13.52
miR-655-3p	20.44	20.97	19.92	19.31	19.04	18.66
miR-656-3p	21.22	20.10	19.98	20.42	20.28	19.49
miR-660-5p	13.90	14.26	14.10	13.82	14.04	13.45
miR-663b	14.34	13.18	11.91	15.84	14.25	14.91
miR-664a-3p	15.30	16.54	17.00	15.93	16.79	16.34
miR-671-3p	19.49	18.73	18.68	18.67	16.38	16.68
miR-708-5p	15.11	17.07	17.17	13.36	15.85	16.57
miR-7-1-3p	15.05	14.69	14.22	14.35	14.32	14.03
miR-7-2-3p	21.57	23.77	21.90	24.53	23.03	22.72
miR-744-3p	20.96	21.54	20.63	20.77	20.00	19.74
miR-744-5p	15.25	15.14	16.02	12.80	13.71	13.64
miR-758-3p	20.30	19.46	19.35	17.70	19.20	18.74
miR-7-5p	21.26	21.59	20.23	21.45	23.53	24.26
miR-766-3p	16.71	18.13	16.11	14.76	13.83	13.41
miR-769-5p	18.67	18.48	18.69	17.69	18.01	18.61
miR-886-3p	14.77	14.03	14.15	14.50	14.00	12.70
miR-886-5p	15.45	15.09	15.43	13.49	13.62	13.02
miR-889-3p	20.26	19.15	18.64	17.32	17.73	16.22
miR-92a-3p	9.84	9.55	9.26	10.36	8.91	9.63
miR-93-3p	18.88	18.92	18.41	17.62	16.97	16.18
miR-93-5p	14.33	14.21	14.05	12.25	13.18	13.42
miR-9-3p	24.38	25.77	24.57	24.82	24.51	23.23
miR-942-5p	18.31	17.05	17.06	17.76	17.52	17.54
miR-95-3p	17.51	15.49	15.91	22.82	23.68	21.66
miR-99a-3p	19.83	19.43	19.10	23.39	24.25	24.64
miR-99a-5p	8.06	8.14	8.96	7.91	8.87	8.52
miR-99b-3p	18.99	18.76	19.00	18.13	17.66	16.41
miR-99b-5p	9.37	9.19	10.13	10.43	10.83	10.95

ND for not detected

Supplementary Table 2. miRNAs differential expression and genetic weight in hASC-EVs *vs* hAMSC-EVs

miRBase ID	hASC-EVs Mean C_{RT}	STD C_{RT}	% Genetic Weight	hAMSC-EVs Mean C_{RT}	STD C_{RT}	% Genetic Weight	hASCs *vs* hAMSCs Ratio	p-value
let-7a-5p	12.63	1.66	0.18804	14.57	0.29	0.03373	3.84	0.11638
let-7c-5p	12.68	0.57	0.18163	17.26	0.12	0.00523	23.93	0.00017
let-7d-5p	16.60	1.27	0.01200	16.35	0.70	0.00984	0.84	0.77752
let-7e-3p	25.51	0.87	0.00003	27.09	0.40	0.00001	3.00	0.04591
let-7g-5p	15.14	0.58	0.03316	14.67	0.53	0.03156	0.72	0.36072
miR-100-3p	24.96	1.33	0.00004	24.43	0.75	0.00004	0.69	0.58030
miR-100-5p	8.37	0.50	3.60273	8.47	0.52	2.31365	1.07	0.82076
miR-101-3p	19.14	0.49	0.00207	20.12	0.19	0.00072	1.97	0.03239
miR-103a-3p	15.20	1.25	0.03181	14.63	0.68	0.03247	0.67	0.52786
miR-106a-5p	11.54	0.53	0.40214	9.40	0.25	1.21658	0.23	0.00324
miR-106b-5p	11.80	0.13	0.33427	11.83	0.36	0.22596	1.02	0.91048
miR-10a-3p	21.65	0.54	0.00036	22.30	0.49	0.00016	1.57	0.19619
miR-10a-5p	12.25	0.80	0.24527	11.92	1.03	0.21289	0.79	0.67981
miR-10b-5p	14.79	1.11	0.04217	19.12	1.76	0.00144	20.13	0.02272
miR-1180-3p	19.23	0.86	0.00195	19.15	0.17	0.00142	0.95	0.88406
miR-1197	22.33	1.47	0.00023	25.12	0.46	0.00002	6.95	0.03467
miR-1226-5p	19.17	0.93	0.00202	20.10	0.43	0.00073	1.90	0.19288
miR-1227-3p	19.02	1.07	0.00225	20.75	0.99	0.00047	3.32	0.10859
miR-1244	23.66	1.24	0.00009	22.98	0.74	0.00010	0.62	0.45791
miR-1254	22.06	0.98	0.00027	22.34	0.55	0.00015	1.21	0.68800
miR-1255b-5p	19.73	1.11	0.00137	20.70	1.04	0.00048	1.95	0.33421
miR-125a-3p	22.50	0.47	0.00020	20.88	0.27	0.00043	0.33	0.00678
miR-125a-5p	14.96	0.28	0.03740	13.72	1.27	0.06084	0.42	0.17289
miR-125b-1-3p	17.50	0.32	0.00646	18.83	0.27	0.00177	2.51	0.00513
miR-125b-5p	6.22	0.41	16.02689	8.27	0.36	2.67123	4.13	0.00278
miR-1260a	12.79	0.76	0.16830	17.95	1.30	0.00326	35.56	0.00411
miR-1262	24.93	0.94	0.00004	24.36	1.24	0.00004	0.68	0.56440
miR-126-3p	20.21	1.54	0.00098	17.51	1.88	0.00442	0.15	0.12550
miR-126-5p	21.60	0.84	0.00037	20.49	0.54	0.00056	0.46	0.12644
miR-1270	22.61	0.39	0.00019	22.55	0.18	0.00013	0.96	0.82767
miR-1271-5p	18.36	1.13	0.00356	18.55	0.27	0.00215	1.14	0.79214
miR-127-3p	10.97	0.56	0.59698	10.26	0.23	0.66920	0.61	0.11546
miR-127-5p	23.98	0.78	0.00007	23.45	0.60	0.00007	0.69	0.40755
miR-1285-3p	19.50	1.51	0.00161	21.44	0.11	0.00029	3.82	0.09140
miR-128a-3p	17.07	0.14	0.00870	17.56	0.72	0.00426	1.41	0.30878
miR-1290	18.15	0.62	0.00410	18.99	0.72	0.00157	1.79	0.20033
miR-1291	14.17	0.11	0.06496	16.28	0.83	0.01036	4.32	0.01220
miR-1303	18.60	0.66	0.00301	19.72	0.43	0.00095	2.17	0.06905
miR-130a-3p	10.88	0.12	0.63248	11.68	0.66	0.25032	1.74	0.10744
miR-130b-3p	13.90	0.14	0.07797	13.31	0.47	0.08110	0.66	0.10380
miR-132-3p	11.27	0.50	0.48266	10.61	0.69	0.52456	0.63	0.25039
miR-133a-3p	19.96	0.39	0.00117	18.04	0.82	0.00306	0.26	0.02092
miR-134-5p	16.03	0.62	0.01779	12.81	0.33	0.11469	0.11	0.00135
miR-135b-5p	23.00	0.86	0.00014	18.68	1.17	0.00196	0.05	0.00682
miR-136-3p	18.70	0.53	0.00281	16.88	0.34	0.00684	0.28	0.00735
miR-137-3p	15.65	0.78	0.02319	15.75	1.33	0.01497	1.07	0.92210
miR-138-5p	12.61	0.65	0.19066	15.13	0.76	0.02287	5.74	0.01210
miR-139-5p	18.99	0.45	0.00229	18.17	0.78	0.00279	0.57	0.18840
miR-140-3p	18.50	0.46	0.00322	19.15	0.15	0.00142	1.56	0.08287
miR-140-5p	14.62	0.71	0.04737	14.33	0.80	0.04000	0.82	0.65720
miR-142-3p	22.98	0.90	0.00014	20.33	0.20	0.00063	0.16	0.00775
miR-143-3p	13.40	0.68	0.11078	13.97	0.55	0.05134	1.49	0.31951

miR-145-3p	20.26	0.43	0.00095	22.65	0.91	0.00012	5.25	0.01445
miR-145-5p	9.52	0.53	1.62348	9.65	0.80	1.02066	1.10	0.82372
miR-146a-5p	18.45	1.80	0.00334	5.65	0.21	16.38347	0.00	0.00026
miR-146b-3p	21.41	0.25	0.00043	21.79	0.41	0.00023	1.29	0.24907
miR-146b-5p	15.25	0.42	0.03059	12.46	0.32	0.14615	0.14	0.00078
miR-148a-3p	13.77	0.11	0.08532	13.88	1.25	0.05467	1.07	0.89270
miR-148b-3p	17.82	0.33	0.00515	17.28	0.19	0.00517	0.69	0.06976
miR-148b-5p	21.63	0.45	0.00037	22.84	1.03	0.00011	2.30	0.13584
miR-149-5p	13.39	0.32	0.11104	11.82	0.35	0.22733	0.34	0.00459
miR-150-5p	19.86	1.18	0.00126	19.62	0.43	0.00102	0.85	0.76249
miR-151a-3p	15.41	0.63	0.02750	14.89	0.49	0.02713	0.70	0.32481
miR-151a-5p	18.88	0.60	0.00247	19.37	0.47	0.00121	1.40	0.32601
miR-152-3p	11.15	0.23	0.52453	10.90	0.11	0.42933	0.84	0.16680
miR-154-3p	20.69	0.10	0.00071	22.17	1.26	0.00017	2.79	0.11268
miR-154-5p	18.27	0.27	0.00377	18.65	1.06	0.00200	1.30	0.58387
miR-155-5p	18.19	0.57	0.00400	14.07	1.65	0.04794	0.06	0.01495
miR-15a-3p	22.00	1.09	0.00029	23.29	0.38	0.00008	2.45	0.12387
miR-15a-5p	17.62	0.91	0.00593	18.59	1.00	0.00208	1.96	0.28176
miR-16-1-3p	22.38	0.28	0.00022	22.74	0.27	0.00012	1.29	0.18519
miR-16-5p	11.40	0.20	0.44209	11.40	0.76	0.30443	1.00	1.00000
miR-17-5p	11.40	0.35	0.44209	9.36	0.17	1.24934	0.24	0.00083
miR-181a-2-3p	20.07	0.99	0.00109	17.81	0.61	0.00358	0.21	0.02820
miR-181a-3p	20.30	0.71	0.00093	18.75	1.12	0.00187	0.34	0.11187
miR-181a-5p	13.47	1.46	0.10553	11.91	1.04	0.21397	0.34	0.20687
miR-181c-5p	19.23	1.12	0.00194	18.74	1.00	0.00188	0.71	0.60038
miR-184	18.15	0.82	0.00411	21.86	0.76	0.00022	13.10	0.00458
miR-185-5p	17.41	0.19	0.00686	17.03	0.31	0.00614	0.77	0.14408
miR-186-5p	15.10	0.26	0.03394	12.75	0.33	0.11907	0.20	0.00063
miR-18a-3p	22.85	0.69	0.00016	22.53	0.35	0.00014	0.80	0.51009
miR-18a-5p	17.80	0.55	0.00522	17.26	0.35	0.00524	0.69	0.22355
miR-190a-5p	18.69	1.17	0.00282	22.66	1.15	0.00012	15.65	0.01378
miR-190b-5p	23.12	0.34	0.00013	24.40	1.01	0.00004	2.42	0.10661
miR-191-3p	24.24	0.63	0.00006	21.66	0.51	0.00025	0.17	0.00515
miR-191-5p	9.56	0.04	1.58274	7.26	0.33	5.37961	0.20	0.00029
miR-192-5p	16.77	0.39	0.01069	15.42	0.19	0.01880	0.39	0.00547
miR-193a-3p	23.98	1.20	0.00007	21.42	0.97	0.00029	0.17	0.04564
miR-193a-5p	12.86	0.04	0.16033	12.11	0.65	0.18636	0.59	0.11502
miR-193b-3p	8.13	0.03	4.27450	8.44	1.03	2.36882	1.24	0.62762
miR-193b-5p	16.83	0.13	0.01025	18.38	1.18	0.00240	2.94	0.08501
miR-194-5p	19.86	1.26	0.00126	19.18	1.36	0.00139	0.62	0.55797
miR-195-5p	13.24	0.29	0.12349	17.34	0.58	0.00494	17.20	0.00040
miR-196b-5p	19.82	1.09	0.00129	23.95	1.68	0.00005	17.52	0.02318
miR-197-3p	11.49	0.35	0.41632	11.35	1.05	0.31531	0.91	0.84086
miR-198	20.66	1.29	0.00072	23.39	0.19	0.00007	6.66	0.02238
miR-199a-3p	10.38	0.38	0.89860	11.06	0.56	0.38569	1.60	0.15468
miR-199a-5p	15.36	0.41	0.02847	17.45	0.63	0.00461	4.26	0.00842
miR-199b-5p	15.14	0.43	0.03316	18.20	0.04	0.00272	8.38	0.00025
miR-19a-3p	15.32	0.26	0.02921	13.32	0.09	0.08026	0.25	0.00024
miR-19b-1-5p	21.97	0.46	0.00029	20.53	0.26	0.00054	0.37	0.00882
miR-19b-3p	9.96	0.22	1.19949	7.49	0.06	4.57520	0.18	0.00005
miR-203a-3p	19.26	0.43	0.00191	18.27	0.47	0.00260	0.50	0.05456
miR-204-5p	15.14	1.28	0.03301	19.13	0.24	0.00143	15.85	0.00611
miR-20a-3p	22.87	0.48	0.00016	23.04	1.22	0.00010	1.12	0.83392
miR-20a-5p	9.69	0.26	1.44302	8.95	0.04	1.66075	0.60	0.00791
miR-20b-5p	18.73	1.14	0.00275	16.33	0.29	0.00996	0.19	0.02423
miR-210-3p	12.88	0.04	0.15885	10.65	0.26	0.51293	0.21	0.00012

miR-212-3p	15.40	0.46	0.02769	14.72	0.41	0.03054	0.62	0.12960
miR-214-3p	10.34	0.24	0.92173	10.37	0.62	0.62222	1.02	0.94421
miR-214-5p	17.60	0.26	0.00601	18.44	0.12	0.00231	1.79	0.00696
miR-21-5p	7.57	0.42	6.27271	7.76	0.30	3.80748	1.13	0.57381
miR-218-2-3p	23.49	1.03	0.00010	26.48	1.14	0.00001	7.95	0.02788
miR-218-5p	10.61	0.43	0.76618	11.29	0.13	0.32756	1.61	0.05748
miR-219a-5p	23.05	0.35	0.00014	23.70	1.55	0.00006	1.57	0.51932
miR-221-3p	7.67	0.49	5.85265	8.26	0.31	2.67864	1.50	0.15261
miR-221-5p	21.57	0.69	0.00038	25.46	1.91	0.00002	14.78	0.02945
miR-222-3p	7.84	0.26	5.20208	7.40	0.59	4.86634	0.74	0.29980
miR-222-5p	16.18	0.53	0.01613	17.94	0.30	0.00327	3.39	0.00724
miR-223-3p	20.44	0.55	0.00084	20.48	0.91	0.00056	1.02	0.96128
miR-22-3p	12.88	0.14	0.15812	12.75	0.30	0.11945	0.91	0.52628
miR-224-5p	11.10	0.35	0.54302	12.69	0.71	0.12461	3.00	0.02570
miR-22-5p	15.36	0.23	0.02834	17.02	0.23	0.00617	3.16	0.00092
miR-23a-3p	13.49	0.23	0.10360	14.83	0.70	0.02821	2.53	0.03458
miR-23a-5p	21.85	0.56	0.00032	23.84	0.56	0.00005	3.95	0.01241
miR-24-2-5p	17.10	0.21	0.00852	19.18	0.53	0.00138	4.24	0.00315
miR-24-3p	6.12	0.22	17.21693	5.54	0.09	17.70194	0.67	0.01369
miR-25-3p	12.57	0.42	0.19648	13.31	0.29	0.08104	1.67	0.06448
miR-26a-1-3p	27.05	0.75	0.00001	26.74	0.87	0.00001	0.81	0.66774
miR-26a-2-3p	26.19	1.27	0.00002	27.30	1.19	0.00000	2.15	0.33110
miR-26a-5p	11.04	0.69	0.56739	11.45	0.90	0.29501	1.32	0.56892
miR-26b-3p	24.80	0.45	0.00004	23.38	0.62	0.00008	0.38	0.03265
miR-26b-5p	13.05	0.50	0.14119	13.21	0.30	0.08680	1.12	0.65301
miR-27a-3p	10.26	0.27	0.97429	12.74	0.82	0.12009	5.59	0.00752
miR-27a-5p	19.48	0.32	0.00163	18.94	1.16	0.00163	0.69	0.47770
miR-27b-3p	11.72	0.40	0.35497	13.11	0.74	0.09320	2.62	0.04494
miR-27b-5p	20.15	0.84	0.00102	21.58	0.37	0.00026	2.69	0.05487
miR-28-3p	13.49	0.07	0.10384	12.71	0.32	0.12264	0.58	0.01535
miR-28-5p	13.10	0.51	0.13638	13.66	1.05	0.06363	1.48	0.45152
miR-296-3p	20.66	0.30	0.00072	20.01	0.43	0.00078	0.64	0.09767
miR-296-5p	12.83	0.37	0.16445	12.78	0.73	0.11691	0.97	0.92733
miR-299-5p	20.00	0.33	0.00114	20.00	0.59	0.00079	1.00	0.98781
miR-29a-3p	10.25	0.34	0.98107	9.97	0.99	0.82198	0.82	0.66389
miR-29a-5p	14.32	0.25	0.05828	17.51	0.62	0.00440	9.13	0.00115
miR-29b-3p	13.70	0.72	0.08998	15.80	1.03	0.01442	4.30	0.04382
miR-29c-3p	11.42	0.48	0.43601	12.42	0.62	0.15005	2.00	0.09116
miR-29c-5p	24.48	1.12	0.00005	25.08	0.56	0.00002	1.52	0.45370
miR-301a-3p	15.66	0.05	0.02302	13.31	0.31	0.08117	0.20	0.00020
miR-301b-3p	20.15	0.20	0.00103	17.96	0.58	0.00322	0.22	0.00339
miR-30a-3p	13.59	0.15	0.09666	11.82	0.22	0.22827	0.29	0.00031
miR-30a-5p	10.84	0.16	0.65026	12.45	0.63	0.14730	3.04	0.01300
miR-30b-5p	8.99	0.19	2.34419	8.72	0.45	1.95455	0.83	0.38278
miR-30c-5p	8.61	0.21	3.05765	8.10	0.30	3.00737	0.70	0.07172
miR-30d-3p	13.35	0.15	0.11416	23.32	1.26	0.00008	998.30	0.00017
miR-30d-5p	21.93	0.36	0.00030	15.54	0.82	0.01724	0.01	0.00025
miR-30e-3p	13.38	0.25	0.11181	11.96	0.44	0.20683	0.37	0.00791
miR-31-3p	11.80	0.30	0.33582	12.15	0.60	0.18152	1.27	0.41762
miR-31-5p	9.83	1.05	1.31260	8.47	1.59	2.31418	0.39	0.28403
miR-320a-3p	11.40	0.20	0.44209	9.56	0.37	1.09114	0.28	0.00161
miR-320b	16.27	0.23	0.01508	16.72	0.58	0.00762	1.36	0.27861
miR-323a-3p	17.83	0.85	0.00514	14.39	0.24	0.03833	0.09	0.00252
miR-324-3p	16.74	0.20	0.01089	15.04	0.99	0.02449	0.31	0.04242
miR-324-5p	14.00	0.51	0.07309	14.07	0.54	0.04792	1.05	0.87725
miR-32-5p	22.84	0.27	0.00016	24.57	1.72	0.00003	3.33	0.15958

miR-328-3p	11.04	0.22	0.56739	10.71	1.16	0.48977	0.80	0.65789
miR-329-3p	19.71	0.65	0.00139	18.60	0.95	0.00207	0.46	0.17045
miR-330-3p	17.33	0.42	0.00725	15.76	0.69	0.01478	0.34	0.02840
miR-331-3p	12.05	0.59	0.28109	11.29	0.82	0.32832	0.59	0.26238
miR-335-3p	18.83	0.46	0.00257	15.11	0.94	0.02321	0.08	0.00360
miR-335-5p	13.63	0.74	0.09402	10.25	0.73	0.67776	0.10	0.00480
miR-337-3p	20.66	0.66	0.00072	21.67	0.09	0.00025	2.02	0.05775
miR-337-5p	18.24	0.55	0.00385	17.19	0.34	0.00550	0.48	0.04810
miR-339-3p	18.17	0.12	0.00405	16.12	0.65	0.01154	0.24	0.00594
miR-33a-5p	22.89	0.31	0.00015	23.02	0.09	0.00010	1.09	0.53817
miR-340-3p	22.51	0.87	0.00020	23.27	0.64	0.00008	1.70	0.28477
miR-340-5p	20.96	0.43	0.00058	20.70	1.06	0.00048	0.83	0.70837
miR-342-3p	14.63	0.27	0.04701	11.34	0.28	0.31824	0.10	0.00012
miR-345-5p	17.10	0.24	0.00850	14.33	0.53	0.03983	0.15	0.00123
miR-34a-3p	13.82	0.18	0.08280	13.92	0.27	0.05310	1.07	0.60948
miR-34a-5p	10.76	0.37	0.69052	10.54	0.17	0.55408	0.86	0.39612
miR-34b-3p	14.95	0.30	0.03783	15.94	0.31	0.01310	1.99	0.01615
miR-34b-5p	18.75	0.05	0.00271	21.81	1.00	0.00022	8.36	0.00608
miR-34c-5p	14.68	0.23	0.04541	15.69	0.34	0.01551	2.02	0.01297
miR-361-5p	13.71	0.12	0.08915	14.00	0.31	0.05027	1.22	0.20735
miR-362-3p	20.15	0.28	0.00103	20.51	0.21	0.00055	1.28	0.15400
miR-362-5p	19.89	0.97	0.00123	19.10	0.28	0.00147	0.58	0.24511
miR-365a-3p	12.69	0.36	0.18038	12.71	0.41	0.12264	1.01	0.95600
miR-369-3p	19.67	0.83	0.00144	20.01	0.72	0.00078	1.27	0.61322
miR-369-5p	23.54	0.41	0.00010	22.25	0.16	0.00016	0.41	0.00709
miR-372-3p	21.84	1.05	0.00032	17.20	1.77	0.00546	0.04	0.01749
miR-373-3p	20.99	0.61	0.00057	20.68	1.23	0.00049	0.80	0.71103
miR-374a-5p	14.48	0.74	0.05216	14.78	0.78	0.02930	1.23	0.66008
miR-374b-5p	15.00	0.71	0.03652	15.40	1.03	0.01896	1.33	0.60228
miR-375-3p	22.01	0.65	0.00028	23.96	1.41	0.00005	3.85	0.09604
miR-376a-3p	12.56	0.73	0.19739	10.78	0.63	0.46711	0.29	0.03306
miR-376b-3p	20.44	0.66	0.00084	19.67	0.68	0.00099	0.59	0.23074
miR-376c-3p	13.04	0.33	0.14218	10.44	0.18	0.59385	0.16	0.00029
miR-377-5p	22.10	0.12	0.00027	22.92	0.86	0.00010	1.76	0.17834
miR-378-3p	19.97	0.81	0.00116	21.09	0.44	0.00037	2.18	0.10293
miR-378a-5p	22.10	0.87	0.00027	24.22	0.24	0.00004	4.36	0.01510
miR-379-5p	16.43	0.83	0.01358	15.69	1.15	0.01562	0.60	0.41738
miR-380-3p	21.89	1.15	0.00031	20.83	1.25	0.00044	0.48	0.34225
miR-380-5p	20.62	1.09	0.00074	21.56	0.23	0.00027	1.92	0.21659
miR-381-3p	18.89	0.66	0.00246	19.32	0.55	0.00126	1.35	0.43617
miR-382-5p	10.98	0.81	0.59149	10.26	0.48	0.67245	0.61	0.25318
miR-409-3p	12.05	0.63	0.28109	10.94	1.27	0.41769	0.46	0.24588
miR-409-5p	19.93	0.55	0.00119	19.41	0.29	0.00118	0.70	0.21503
miR-410-3p	15.51	0.31	0.02566	14.35	0.64	0.03929	0.45	0.04856
miR-411-3p	22.56	0.18	0.00019	22.20	0.65	0.00017	0.78	0.41338
miR-411-5p	14.85	0.73	0.04055	14.18	0.94	0.04424	0.63	0.38900
miR-422a	23.37	0.34	0.00011	22.91	0.25	0.00010	0.73	0.13740
miR-423-5p	15.34	0.27	0.02874	15.24	1.10	0.02133	0.93	0.87597
miR-424-3p	15.80	0.09	0.02089	16.28	0.77	0.01033	1.39	0.34796
miR-424-5p	15.09	0.84	0.03433	16.08	0.62	0.01188	1.99	0.17379
miR-425-3p	20.95	0.16	0.00059	19.31	0.88	0.00126	0.32	0.03337
miR-432-3p	18.07	1.01	0.00434	16.09	0.96	0.01176	0.25	0.07046
miR-433-3p	18.43	0.48	0.00339	16.89	0.46	0.00677	0.34	0.01648
miR-449b-5p	25.22	0.51	0.00003	23.36	0.70	0.00008	0.28	0.02036
miR-450a-5p	21.38	0.49	0.00044	21.80	0.23	0.00022	1.34	0.24635
miR-451a	21.05	0.08	0.00055	23.28	1.38	0.00008	4.67	0.04982

miR-452-5p	16.80	0.31	0.01047	17.40	1.00	0.00477	1.51	0.37863
miR-454-5p	26.38	0.28	0.00001	24.03	0.47	0.00005	0.20	0.00174
miR-455-3p	17.11	0.99	0.00845	16.12	0.46	0.01155	0.50	0.19167
miR-455-5p	18.03	0.51	0.00447	16.45	0.21	0.00916	0.34	0.00768
miR-483-5	13.96	1.00	0.07480	12.95	1.20	0.10421	0.49	0.32196
miR-484	11.49	0.10	0.41440	9.11	0.29	1.48538	0.19	0.00017
miR-485-3p	18.03	0.80	0.00445	18.52	1.24	0.00219	1.40	0.59818
miR-485-5p	17.70	0.50	0.00560	17.04	0.89	0.00612	0.63	0.32080
miR-487a-3p	21.19	0.34	0.00050	20.22	0.67	0.00068	0.51	0.08857
miR-487b-3p	18.19	0.56	0.00399	16.59	0.04	0.00833	0.33	0.00770
miR-490-3p	ND	ND	0.00000	19.16	0.53	0.00140		
miR-491-5p	17.34	0.63	0.00719	17.31	0.30	0.00505	0.98	0.94935
miR-493-3p	19.56	0.37	0.00155	16.63	0.54	0.00812	0.13	0.00153
miR-494-3p	15.81	0.21	0.02084	14.13	0.41	0.04592	0.31	0.00312
miR-495-3p	15.04	0.66	0.03546	13.55	0.48	0.06875	0.36	0.03439
miR-500a-5p	18.62	0.70	0.00297	19.63	0.67	0.00102	2.01	0.14681
miR-502-3p	18.63	0.30	0.00295	19.55	0.19	0.00107	1.90	0.01063
miR-502-5p	19.51	0.63	0.00160	19.74	0.47	0.00094	1.17	0.63649
miR-503-5p	16.38	0.39	0.01401	15.16	0.29	0.02253	0.43	0.01186
miR-505-3p	18.56	0.47	0.00309	19.47	0.48	0.00113	1.88	0.08004
miR-505-5p	20.90	0.17	0.00061	22.07	0.61	0.00019	2.25	0.03288
miR-532-3p	15.64	0.44	0.02340	14.34	0.54	0.03961	0.41	0.03263
miR-532-5p	13.86	0.23	0.08053	12.69	0.21	0.12435	0.45	0.00300
miR-539-5p	18.24	0.31	0.00387	15.42	0.62	0.01881	0.14	0.00215
miR-542-3p	18.90	0.48	0.00244	20.26	1.07	0.00066	2.56	0.11671
miR-542-5p	20.85	0.30	0.00063	19.47	1.02	0.00113	0.38	0.08701
miR-543	17.42	0.92	0.00683	17.68	0.82	0.00391	1.20	0.72650
miR-544Aa	27.40	1.43	0.00001	26.84	0.92	0.00001	0.68	0.60117
miR-545-3p	23.76	1.00	0.00008	23.97	0.75	0.00005	1.15	0.78940
miR-548am-5p	20.36	0.49	0.00089	21.58	0.67	0.00026	2.33	0.06339
miR-548b-5	19.95	0.60	0.00118	20.93	0.50	0.00041	1.97	0.09701
miR-548d-3p	23.16	0.42	0.00013	26.12	0.99	0.00001	7.79	0.00888
miR-548d-5	20.64	0.55	0.00073	21.80	0.92	0.00022	2.24	0.13424
miR-570-3p	25.41	0.54	0.00003	25.67	0.74	0.00002	1.19	0.66096
miR-572	18.83	0.33	0.00257	21.27	0.17	0.00033	5.44	0.00035
miR-574-3p	10.45	0.20	0.85407	8.48	0.44	2.30085	0.26	0.00221
miR-576-3p	19.25	0.12	0.00192	19.22	0.50	0.00134	0.98	0.94227
miR-579-3p	20.25	0.59	0.00096	21.51	0.74	0.00028	2.39	0.08277
miR-589-3p	19.45	0.33	0.00167	19.63	0.46	0.00101	1.13	0.60609
miR-590-3p	21.58	0.42	0.00038	21.13	0.85	0.00036	0.73	0.45786
miR-590-5p	16.13	0.18	0.01670	14.79	0.14	0.02913	0.39	0.00051
miR-597-5p	19.30	0.27	0.00185	19.79	0.50	0.00091	1.40	0.21641
miR-598-3p	21.94	0.97	0.00030	18.72	0.30	0.00190	0.11	0.00533
miR-601	15.42	1.84	0.02719	22.91	0.70	0.00010	179.44	0.00275
miR-605-5p	19.53	0.62	0.00157	22.29	1.06	0.00016	6.77	0.01762
miR-616-3p	20.71	0.50	0.00070	19.87	0.22	0.00086	0.56	0.05462
miR-616-5p	20.75	0.62	0.00068	22.19	0.64	0.00017	2.72	0.04825
miR-622	22.87	1.61	0.00016	24.22	0.86	0.00004	2.55	0.26991
miR-625-3p	18.35	0.47	0.00357	18.52	0.84	0.00218	1.13	0.77340
miR-625-5p	22.73	0.32	0.00017	21.70	0.78	0.00024	0.49	0.10282
miR-628-3p	22.65	0.29	0.00018	24.41	0.80	0.00004	3.39	0.02332
miR-628-5p	21.78	0.27	0.00033	21.75	0.65	0.00023	0.98	0.93830
miR-629-3p	18.94	0.69	0.00238	17.52	0.78	0.00439	0.37	0.07764
miR-629-5p	23.06	0.06	0.00014	21.61	0.45	0.00026	0.36	0.00515
miR-638	17.36	0.38	0.00712	20.29	1.16	0.00064	7.62	0.01426
miR-642a-5p	19.11	0.20	0.00211	18.00	0.29	0.00314	0.46	0.00555

miR-652-3p	19.09	0.44	0.00214	18.02	1.00	0.00309	0.48	0.16648
miR-654-3p	21.10	0.67	0.00053	19.84	0.82	0.00087	0.42	0.10825
miR-654-5p	15.01	0.70	0.03629	13.67	0.19	0.06309	0.40	0.03322
miR-655-3p	20.44	0.53	0.00084	19.00	0.33	0.00157	0.37	0.01556
miR-656-3p	20.43	0.68	0.00085	20.06	0.50	0.00075	0.77	0.49049
miR-660-5p	14.09	0.18	0.06867	13.77	0.30	0.05890	0.80	0.19028
miR-663b	13.14	1.22	0.13204	15.00	0.80	0.02513	3.62	0.09212
miR-664a-3p	16.28	0.88	0.01501	16.35	0.43	0.00981	1.05	0.90088
miR-671-3p	18.97	0.45	0.00233	17.24	1.25	0.00531	0.30	0.08776
miR-708-5p	16.45	1.16	0.01334	15.26	1.68	0.02099	0.44	0.37008
miR-7-1-3p	14.65	0.42	0.04648	14.23	0.17	0.04275	0.75	0.18540
miR-7-2-3p	22.41	1.19	0.00021	23.43	0.97	0.00007	2.02	0.31614
miR-744-3p	21.04	0.46	0.00055	20.17	0.53	0.00070	0.54	0.09762
miR-744-5p	15.47	0.48	0.02632	13.38	0.50	0.07704	0.24	0.00654
miR-758-3p	19.70	0.52	0.00140	18.54	0.77	0.00215	0.45	0.09658
miR-7-5p	21.03	0.71	0.00056	23.08	1.46	0.00009	4.16	0.09374
miR-766-3p	16.98	1.04	0.00924	14.00	0.69	0.05018	0.13	0.01441
miR-769-5p	18.61	0.12	0.00298	18.10	0.47	0.00292	0.70	0.14107
miR-886-3p	14.32	0.40	0.05855	13.73	0.93	0.06043	0.67	0.37293
miR-886-5p	15.32	0.20	0.02914	13.37	0.32	0.07749	0.26	0.00084
miR-889-3p	19.35	0.83	0.00179	17.09	0.78	0.00590	0.21	0.02625
miR-92a-3p	9.55	0.29	1.59375	9.63	0.73	1.03563	1.06	0.86197
miR-93-3p	18.74	0.28	0.00273	16.92	0.72	0.00662	0.28	0.01530
miR-93-5p	14.20	0.14	0.06363	12.95	0.62	0.10389	0.42	0.02717
miR-9-3p	24.90	0.75	0.00004	24.19	0.84	0.00004	0.61	0.33282
miR-942-5p	17.47	0.72	0.00657	17.60	0.13	0.00413	1.09	0.77506
miR-95-3p	16.30	1.07	0.01477	22.72	1.02	0.00012	85.35	0.00166
miR-99a-3p	19.45	0.37	0.00166	24.09	0.64	0.00005	24.94	0.00040
miR-99a-5p	8.39	0.50	3.56958	8.43	0.49	2.37924	1.03	0.91251
miR-99b-3p	18.92	0.14	0.00241	17.40	0.89	0.00476	0.35	0.04333
miR-99b-5p	9.56	0.50	1.57909	10.74	0.27	0.48102	2.26	0.02318

ND for not detected

Supplementary Table 3. miRNAs differential expression in hASC-EVs *vs* hAMSC-EVs first quartile of expression among those with genetic weight ≥ 1% in one of the two EV types

miRBase ID	hASC-EVs % Genetic Weight	hAMSC-EVs % Genetic Weight	hASC-EVs *vs* hAMSC-EVs Ratio	*p*-value
miR-125b-5p	**16.02689**	**2.67123**	***4.13***	***0.00278***
miR-99b-5p	**1.57909**	**0.48102**	***2.26***	***0.02318***
miR-221-3p	**5.85265**	**2.67864**	1.50	0.15261
miR-193b-3p	**4.27450**	**2.36882**	1.24	0.62762
miR-21-5p	**6.27271**	**3.80748**	1.13	0.57381
miR-145-5p	**1.62348**	**1.02066**	1.10	0.82372
miR-100-5p	**3.60273**	**2.31365**	1.07	0.82076
miR-92a-3p	**1.59375**	**1.03563**	1.06	0.86197
miR-99a-5p	**3.56958**	**2.37924**	1.03	0.91251
miR-30b-5p	**2.34419**	**1.95455**	0.83	0.38278
miR-222-3p	**5.20208**	**4.86634**	0.74	0.29980
miR-30c-5p	**3.05765**	**3.00737**	0.70	0.07172
miR-24-3p	**17.21693**	**17.70194**	0.67	0.01369
miR-20a-5p	**1.44302**	**1.66075**	0.60	0.00791
miR-31-5p	**1.31260**	**2.31418**	0.39	0.28403
miR-320a-3p	**0.44209**	**1.09114**	***0.28***	***0.00161***
miR-574-3p	**0.85407**	**2.30085**	***0.26***	***0.00221***
miR-17-5p	**0.44209**	**1.24934**	***0.24***	***0.00083***
miR-106a-5p	**0.40214**	**1.21658**	***0.23***	***0.00324***
miR-191-5p	**1.58274**	**5.37961**	***0.20***	***0.00029***
miR-484	**0.41440**	**1.48538**	***0.19***	***0.00017***
miR-19b-3p	**1.19949**	**4.57520**	***0.18***	***0.00005***
miR-146a-5p	0.00334	**16.38347**	***0.00***	***0.00026***

In bold, miRNAs in ASC-EVs or AMSC-EVs first quartile of expression***; in bold and italics, differentially abundant miRNAs (ratio > 2 or < 0.5 with p-value < 0.05)***

Supplementary Table 4

	miR-24-3p	miR-125b-5p	miR-222-3p	miR-21-5p	miR-221-3p
hASC-EVs %	17.22	16.03	5.20	6.27	5.85
hAMSC-EVs%	17.70	2.67	4.87	3.81	2.68
	ABCB9	ABTB1	ABCB1	ABCB1	ADAM1A
	ACVR1B	AHRR	ABCG2	AKT2	ADAMTS6
	AGPAT2	AKT1	ADAM1A	ANKRD46	ANXA1
	ARHGAP19	ALOX5	ARID1A	ANP32A	APAF1
	ATG4A	ANGPT2	ARTN	APAF1	ARF4
	AURKB	APC	ATM	BASP1	ARIH2
	BCAR1	APLN	BBC3	BCL10	ARNT
	BCL2L11	ARID3A	BMF	BCL2	ASZ1
	BRCA1	ARID3B	CAPRIN1	BCL6	BBC3
	CARD10	BAK1	CARM1	BMI1	BCL2L11
	CCNA2	BBC3	CCL3	BMPR2	BECN1
	CCND1	BCL2	CDC27	BTG2	BMF
	CDK1	BCL2L2	CDK2	CADM1	BNIP3
	CDK4	BCL3	CDKN1B	CASC2	BNIP3L
	CDKN1B	BMF	CDKN1C	CASP8	BRAP
	CDKN2A	BMPR1B	CERS2	CBX4	CDKN1B
	CHEK1	BTG2	CFTR	CCL20	CDKN1C
	COPS5	CBFB	CHUK	CCR1	CERS2
	CORO1A	CCNJ	CORO1A	CDC25A	CORO1A
	CYP11B2	CD44	CXCL2	CDK2AP1	CREBZF
	DEDD	CDH5	CYB5A	CEBPB	CTCF
	DHFR	CDKN2A	DICER1	CLU	CXCL12
	DHFRP1	CDKN2D	DIRAS3	COL4A1	DDIT4
	DND1	CEBPA	DKK2	COX2	DICER1
	DYRK2	CGN	E2F1	DAXX	DIRAS3
	E2F2	CSNK2A1	ECT2	DDAH1	DKK2
	EIF2S3	CYP24A1	EGF	DERL1	DVL2
	FAF1	DGAT1	EPB41L3	DNM1L	ESR1
	FBXW7	DKK3	ESR1	DOCK4	ETS1
	FEN1	DRAM2	ETS1	DOCK5	FMR1
	FGF11	DUSP6	FBXO8	DOCK7	FOS
	FGFR3	E2F2	FBXW7	DUSP10	FOXO3
	FSCN1	E2F3	FOS	E2F1	GJA1
	FURIN	EGFR	FOXO1	EGFR	GRB10
	GATA3	EIF4EBP1	FOXO3	EGLN1	HECTD2
	H2AFX	EIF5A2	GAS5	EIF4A2	HMGXB4
	HMOX1	ENPEP	GJA1	ERBB2	HOXB5
	HNF4A	EPO	GNAI2	FASLG	ICAM1
	IFNG	EPOR	GNAI3	FBXO11	KIT
	IFNR	ERBB2	GRB10	FMOD	MBD2
	IL4	ERBB3	HAX1	FOXO1	MDM2

INSIG1	ETS1	HSP90B1	FZD6	MEOX2
JPH2	Fas	ICAM1	GAS5	MGMT
LDHA	FES	IGF1R	GDF5	MMP2
LDHB	FGFR2	IL6	HMGB1	MYBL1
MAFB	FZD6	KIT	HNRNPK	NAIP
MAP3K9	GAB2	LIF	HPGD	PAK1
MAPK14	GLI1	LMO2	ICAM1	PIK3R1
MAPK7	GRIN2A	MAFB	ICOSLG	PTEN
MEN1	GSS	MEF2C	IGF1R	RAB1A
MLEC	HK2	MGMT	IL12A	RAD51
MMP14	HMGA1	MMP1	IL1B	RB1
MT1M	HMGA2	MYL9	IRAK1	RECK
MXI1	HOTTIP	NFIA	ISCU	RUNX1
MYC	ICAM2	NFIX	JAG1	SELE
NCAN	IGF1R	NLRP3	JMY	SIRT1
NCSTN	IGF2	PARP1	LRP6	SOCS1
NDST1	IKZF2	PAX6	LRRFIP1	SOCS3
NOS3	IKZF3	PLXNC1	MAP2K3	SSX2IP
NOTCH1	IKZF4	POLR3G	MARCKS	STAT5A
PAK4	IL6R	PPP2R2A	MEF2C	STMN1
PCNA	IRF4	PRDM1	MSH2	TBK1
PDGFRB	JAK2	PTBP2	MSH6	TCEAL1
POLD1	KLC2	PTEN	MSLN	TICAM1
PRDX6	KLF13	RAB12	MTAP	TIMP3
PRKCH	LACTB	RECK	MYD88	TMED7
PSAP	LIFR	RHOB	NAV3	TNFSF10
PTPN9	LIN28A	RUNX2	NCAPG	TP53
PTPRF	LIN28B	SCARB1	NCOA3	TRPS1
REG4	LIPA	SELE	NFIA	USP18
S100A8	MAN1B1	SEMA3A	NFIB	WEE1
SH3PXD2A	MAP3K11	SLC2A4	NTF3	ZEB2
SLC6A4	MAPK14	SMAD5	OXTR	
SP1	MCL1	SOD2	PCBP1	
SSSCA1	MEGF9	SP1	PCGF2	
ST7L	MMP13	SP3	PDCD4	
STX16	MMP2	SSSCA1	PIAS3	
TACC3	MMP26	SSX2IP	PIK3R1	
TGFB1	MUC1	STAT1	PLAT	
TMED7	MXD1	STAT3	PLOD3	
TMEM92	NCOR2	STAT5A	PPARA	
TNK2	NES	STMN1	PPIF	
TP53	NEU1	TAL1	PSMD9	
TRIB3	NKIRAS2	TCEAL1	PTEN	
TRIM11	NTRK3	TIMP3	PTPN14	
WNT4	PCTP	TMED7	PTX3	
XIAP	PHF8	TNFSF10	RASA1	
ZNF217	PIAS3	TOX	RASGRP1	
	PIGF	TP53	RECK	

PIK3CB	TRPS1	REST
PIK3CD	VGLL4	RHO
PODXL	ZEB1	RHOB
PPP1CA		RPS7
PRDM1		RTN4
PRKRA		SATB1
PTH1R		SERPINB5
RAF1		SERPINI1
RPS6KA1		SETD2
SCNN1A		SIRT2
SEMA4C		SMAD7
SET		SMARCA4
SFRP5		SMN1
SGPL1		SOCS1
SIRT7		SOCS6
SMAD4		SOD3
SMO		SOX2
SPHK1		SOX5
STARD13		SP1
STAT3		SPRY2
SUV39H1		STAT3
TBC1D1		STUB1
TET2		TAP1
TNF		TCF21
TNFAIP3		TGFB2
TP53		TGFBI
TP53INP1		TGFBR2
VDR		TGFBR3
VPS4B		TGIF1
VPS51		TIAM1
		TICAM2
		TIMP3
		TLR3
		TM9SF3
		TNFAIP3
		TNFRSF10B
		TOPORS
		TP53BP2
		TP63
		TPM1
		TRAF7
		UBE2N
		VEGFA
		VHL
		WWP1
		YOD1

miR-193b-3p	miR-30c-5p	miR-191-5p	miR-99a-5p	miR-100-5p	miR-19b-3p	miR-30b-5p
4.27	3.06	1.58	3.57	3.60	1.20	2.34
2.37	3.01	5.38	2.38	2.31	4.58	1.95
AKR1C2	BCL9	BASP1	AGO2	ACKR3	ARID4B	ATG12
CCND1	BECN1	CCND2	AKT1	AKT1	ATXN1	BCL2
ESR1	CAMK2D	CDK6	CAPNS1	ATM	BACE1	BCL6
ETS1	CASP3	CDK9	CTDSPL	BMPR2	BCL2L11	BCL9
KIT	CCND2	CEBPB	FGFR3	CTDSPL	BCL3	BECN1
KRAS	CDC42	CTDSP2	FKBP5	CYR61	BMPR2	CAT
MAX	CTGF	EGR1	HOXA1	FGFR3	CUL5	CCNE2
MCL1	DDIT4	IL1A	IGF1R	FKBP5	CYP19A1	CTHRC1
MYB	DLL4	LRRC8A	MTMR3	FLT1	DNMT1	DLL4
NF1	DNMT1	MDM4	MTOR	HOXA1	ESR1	DNMT1
PLAU	EIF2S1	NDST1	NOX4	HS3ST2	GCM1	EIF2S1
PRAP1	FASN	NOTCH2	RAVER2	IGF1R	HIPK1	EIF5A2
RAD51	FOXO3	RPS6KA3	SERPINE1	IGF2	HIPK3	ERG
SHMT2	HDAC4	SATB1	SMARCA5	MMP13	KAT2B	HOXA1
SMAD3	HSPA4	SLC16A2	TRIB2	MTOR	MTUS1	MBNL1
YWHAZ	IDH1	SOX4		NCOR2	MXD1	MBNL2
	IER2	TMC7		PLK1	MYCN	MBNL3
	IL11	YBX3		RAP1B	MYLIP	NOTCH1
	JAK1			RNF144B	NCOA3	PDGFRB
	MCL1			SMARCA5	PITX1	RASAL2
	MTA1			THAP2	PKNOX1	RUNX2
	MTTP			ZBTB7A	PPP2R5E	SERPINE1
	NCOR2			ZNF215	PRKAA1	SIX1
	NOTCH1			ZNRF2	PTEN	SMAD1
	PAK1				SMAD4	SNAI1
	RARB				SOCS1	SOCS1
	RASAL2				TGFBR2	TP53
	RFX6				TLR2	
	RUNX2				TNFAIP3	
	SERPINE1				TP53	
	SMAD1					
	SNAI1					
	SNAI2					
	SOCS3					
	TGIF2					
	TP53					
	TWF1					
	UBE2I					
	VIM					

miR-31-5p	miR-20a-5p	miR-574-3p	miR-145-5p	miR-92a-3p	miR-29a-3p	miR-99b-5p
1.31	1.44	0.85	1.62	1.59	0.98	1.58
2.31	1.66	2.30	1.02	1.04	0.82	0.48
ABCB9	ABL2	CLTC	ABCC1	ARID4B	ABL1	ARID3A
ARID1A	ANKH	CUL2	ABHD17C	BCL2L11	ADAM12	IGF1R
ARPC5	APP	EGFR	ABRACL	BMPR2	ADAMTS9	MTOR
BAP1	ARHGAP12	EP300	ACTB	CCL8	AHR	RAVER2
C1QTNF9	ATG16L1	RAC1	ADAM17	CD69	AKT2	
CASR	BAMBI	RXRA	ADD3	CDH1	AKT3	
CDK1	BCL2	SMAD4	AKR1B10	CPEB2	ALDH5A1	
CREG1	BCL2L11	TGFB1	ALDH3A1	DNMT1	ATG9A	
CXCL12	BMPR2		ALPPL2	DUSP10	BACE1	
DACT3	BNIP2		ANGPT2	ESR2	BCL2	
DKK1	CCND1		AP1G1	FBXW7	BCL7A	
DMD	CCND2		APH1A	HDAC2	CACNA1C	
DOCK1	CDKN1A		ARF6	HIPK1	CALCR	
E2F2	CRIM1		ARL6IP5	HIPK3	CCND1	
EMSY	DAPK3		BNIP3	IKZF1	CCND2	
ETS1	DNMT1		BRAF	ITGA5	CCNT2	
FOXO3	DUSP2		C11orf65	KAT2B	CD276	
FOXP3	E2F1		CAMK1D	KLF2	CD93	
FZD3	E2F3		CBFB	KLF4	CDC42	
GNA13	EGLN3		CCDC43	LASP1	CDC7	
HIF1AN	EGR2		CD28	MAP2K4	CDK2	
HOXC13	EPAS1		CD40	MAPK8	CDK4	
ICAM1	ETV1		CD44	MAPRE1	CDK6	
IL25	FBXO31		CDH2	MYCBP2	CEACAM6	
ITGA5	GJA1		CDK4	MYLIP	CLDN1	
JAZF1	HIF1A		CDK6	NR1H4	COL10A1	
KLF13	IRF2		CDKN1A	OSBPL2	COL1A2	
LATS2	ITGB8		CEP19	OSBPL8	COL3A1	
MAP4K4	KIF26B		CFTR	PCGF5	COL4A1	
MCM2	KIT		CLINT1	PHLPP1	COL4A2	
MET	LIMK1		COL5A1	PTEN	COL5A2	
MLH1	MAP2K3		CPEB4	RAD21	CPEB3	
MMP16	MAP3K12		CRNDE	RFFL	CPEB4	
MPRIP	MAP3K5		CTGF	RGS5	CYP2C19	
NFAT5	MAPK9		CTNND1	SIRT1	DICER1	
NUMB	MCL1		DDC	SOCS5	DKK1	
PPP2R2A	MEF2D		DDX17	STAT3	DNMT1	
PRKCE	MYC		DDX6	TGFBR2	DNMT3A	
RAB27A	NFKBIB		DFFA	TP63	DNMT3B	
RASA1	NRAS		DTD1		ELN	
RDX	PHLPP2		E2F3		FBN1	

RET	PKD1	EGFR	FGA
RHOA	PKNOX1	EIF4E	FGB
RHOBTB1	PPARG	EPAS1	FGG
SATB2	PPP2R2A	ERG	FOXO3
SELE	PRKG1	ESR1	FSTL1
SGPP2	PTEN	ETS1	GLUL
SLC1A2	PTPRO	F11R	GPR85
SMAD4	PURA	FAM3C	GSK3B
SOX4	RB1	FAM45A	HBP1
SP7	RB1CC1	FLI1	HMGCR
SPRED1	RBL1	FSCN1	IFNAR1
SPRED2	RBL2	FXN	IGF1
SPRY1	REST	FZD7	IMPDH1
SPRY3	RGS5	GMFB	ITGA11
SPRY4	RUNX1	GOLM1	ITGA6
SRC	RUNX3	HDAC11	ITGB1
STK40	SIRPA	HDAC2	ITIH5
STMN1	SMAD4	HLTF	KDM5B
TBXA2R	SMAD7	HMGA2	KEAP1
TIAM1	STAT3	HOXA9	KLF4
WASF3	TCEAL1	IFNB1	KREMEN2
XRCC5	TGFBR1	IGF1R	LAMC2
YY1	TGFBR2	ILK	LOX
	TIMP2	IRS1	LPL
	TP53INP1	IRS2	MCL1
	TSG101	ITGB8	MMP2
	UBE2C	JADE1	MUC1
	VEGFA	JAG1	MYC
	WEE1	KLF4	MYCN
	ZFYVE9	KLF5	NASP
		KREMEN1	NAV3
		LYPLA2	NFIA
		MAP2K6	NMI
		MCM2	PDGFRB
		MDM2	PER1
		MEST	PIK3R1
		MIXL1	PPM1D
		MMP1	PPP1R13B
		MMP12	PTEN
		MMP14	PXDN
		MSH3	QKI
		MTDH	RAN
		MTMR14	RASGRP1
		MUC1	RNASEL
		MYC	ROBO1
		MYO5A	S100B
		MYO6	SAPCD2
		MYOCD	SERPINB9

MYRF
NAIP
NANOG
NDRG2
NDUFA4
NEDD9
NFATC1
NIPSNAP1
NRAS
NUDT1
PAK4
PARP8
PIGF
PODXL
POU5F1
PPM1D
PPP3CA
PTP4A2
PXN
ROBO2
ROCK1
RPA1
RPS6KB1
RREB1
RTKN
SENP1
SERINC5
SERPINE1
SET
SMAD2
SMAD3
SOCS7
SOX2
SOX9
SP1
SP7
SPTBN1
SPTLC1
SRGAP1
STAT1
SWAP70
TGFB2
TGFBR2
TIRAP
TMEM9B
TMOD3
TNFSF13
TPM3
SERPINH1
SETDB1
SFRP2
SLC22A7
SPARC
SRGAP2
TDG
TET1
TET2
TET3
TFEB
TNFAIP3
TRAF4
TRIM68
VDAC1
VEGFA
ZFP36

TPRG1
TSPAN6
TUG1
VEGFA
VPS51
YES1

miR-484	miR-214-3p	miR-17-5p	miR-106a-5p	miR-320a-3p	miR-127-3p	miR-382-5p
0.41	0.92	0.44	0.40	0.44	0.60	0.59
1.49	0.62	1.25	1.22	1.09	0.67	0.67
FIS1	ALPK2	ABCA1	APC	ABCG2	BAG5	DRD1
SMAD2	ARL2	ADAR	APP	AQP1	BCL6	MXD1
ZEB1	ASF1B	APP	ARID4B	AQP4	KMT5A	NFIA
	ATF4	BCL2	ATG7	AR	MAPK4	PTEN
	BAX	BCL2L11	ATM	ARF1	MGMT	YBX1
	BCL2L11	BMP2	BCL10	ARPP19	MMP13	
	BCL2L2	BMPR2	BMP2	BANP	PRDM1	
	BIRC5	BRCA2	CASP7	BMI1	RGMA	
	CADM1	CCL1	CCND1	CDK6	SEPT7	
	CCL5	CCND1	CDKN1A	CRKL	SERPINB9	
	CD274	CCND2	CDX2	CTNNB1	SFRP1	
	CDK6	CDKN1A	CXCL8	ESRRG	SKI	
	CPD	CLOCK	CYP19A1	FAS	XBP1	
	CTNNB1	CLU	E2F1	FH	XRCC3	
	EZH2	CYP7B1	ERCC1	FOXM1	ZWINT	
	FGFR1	DAPK3	FAS	GNAI1		
	GALNT7	DNAJC27	FASTK	HMGB1		
	GSR	DNMT1	HIF1A	HOXA10		
	HDGF	E2F1	HIPK3	HSPB6		
	ING4	E2F3	HMGA2	IGF1R		
	JAG1	EGR2	IL10	ITGB3		
	LTF	EPAS1	LIMK1	KITLG		
	LZTS1	ETV1	MAPK9	MAPK1		
	MAP2K3	FBXO31	MFN2	MCL1		
	MAPK1	GPR137B	MGST2	MTDH		
	MAPK8	HBP1	MYB	MYC		
	MEF2C	HIF1A	MYLIP	NFATC3		
	NRAS	HSPB2	PTEN	NOD2		
	PAPPA	ICAM1	RARB	NPR1		
	PIM1	IGFBP3	RB1	NRP1		
	PLXNB1	ITGB8	RBL2	PBX3		
	POR	JAK1	RND3	PDCD4		
	POU4F2	KAT2B	RUNX1	PICSAR		
	PSMD10	LDLR	RUNX3	POLR3D		
	PTEN	LIMK1	SIRPA	PTEN		
	QKI	MAP3K12	SLC2A3	RAB11A		
	RAB15	MAPK9	STAT3	RAB14		
	SEMA4D	MDM2	TGFBR2	RAC1		
	SRGAP1	MEF2D	TIMP2	RUNX2		
	SRGAP2	MFN2	ULK1	SUZ12		
	SUFU	MMP2	VEGFA	TAC1		

TP53	MYC	TFRC
TWIST1	NABP1	TRPC5
UBE2I	NCOA3	USP14
XBP1	NPAS3	VDAC1
	NPAT	VEGFA
	PDLIM7	VIM
	PHLPP1	YWHAZ
	PKD2	
	PKNOX1	
	PPP2R2A	
	PTEN	
	PTPRO	
	RAD21	
	RB1	
	RBL1	
	RBL2	
	RND3	
	RUNX1	
	SELE	
	SIRPA	
	SMAD4	
	SMURF1	
	SOCS6	
	STAT3	
	TBC1D2	
	TCEAL1	
	TCF3	
	TGFBR2	
	TIMP3	
	TLR7	
	TNF	
	TNFSF12	
	TP53COR1	
	TP53INP1	
	TRIM8	
	UBE2C	
	VEGFA	
	VLDLR	
	WEE1	
	YES1	
	ZBTB4	
	ZFYVE9	
	ZNFX1	

miR-34a-5p	miR-199a-3p	miR-328-3p	miR-132-3p	miR-218-5p	miR-152-3p	miR-26a-5p
0.69	0.90	0.57	0.48	0.77	0.52	0.57
0.55	0.39	0.49	0.52	0.33	0.43	0.30
ACSL1	AKT1	ABCG2	AGO2	ACTN1	ADAM17	ABCA1
ACSL4	APOE	BACE1	ARHGAP32	BCL9	ALCAM	ACVR1
AGTR1	CAV2	CD44	BDNF	BIRC5	ATG14	ADAM17
AIP	CD44	H2AFX	CCNA2	BIRC6	CCKBR	AMACR
AKT1	CDK7	KCNH2	CCNB1	BMI1	CCND1	ARL4C
ANK3	DNAJA4	MMP16	CDKN1A	CDH2	CD151	ATM
AR	FLT1	PLCE1	CRK	CDK6	CD274	BAG4
ARHGDIB	FOXA2	PTPRJ	EGFR	CDKN1B	CSF1	CCND2
ATG4A	FUT4	SFRP1	FOXO1	DKK2	DKK1	CCNE1
ATG4B	HGF		GDF5	E2F2	DNMT1	CCNE2
ATG4C	IGF1		HBEGF	EBP	FGF2	CDC6
ATG4D	ITGA3		IRAK4	EFNA1	FGFR3	CDK6
ATG5	KDR		JPT1	EGFR	HLA-G	CDK8
ATG7	MAPK1		KLHL11	GJA1	IGF1R	CHD1
ATP5S	MAPK14		MAPK1	GLI2	IRS1	CHEK1
AXIN2	MAPK8		MMP9	HMGB1	ITGA5	CKS2
AXL	MAPK9		MUC13	HMOX1	KLF4	CPEB2
BAX	MET		PIK3R3	HOXB3	KRAS	CPEB3
BCL2	MTOR		RAF1	IKBKB	MAFB	CPEB4
BECN1	PAK4		RASA1	KIT	NRP1	CTGF
BIRC5	PTGS2		RB1	LAMB3	PIK3R3	DNMT3B
BMP7	SMARCA2		SIRT1	LASP1	PTEN	DUSP4
CACNB3	STK11		SLC2A1	LEF1	TACC3	DUSP5
CCL22	TFAM		SMAD2	LGR4	TGFA	E2F2
CCND1	VEGFA		SOX4	MBNL2	WNT1	ESR1
CCND3	YAP1		SOX5	MITF	XIST	EZH2
CCNE2	ZHX1		SOX6	MMP2		FGF9
CD24			SPRED1	MRPS27		FUT8
CD44			SPRY1	NUP93		GDAP1
CDC25A			TJAP1	OTUD7B		GSK3B
CDK4			TLN2	PDGFRA		HGF
CDK6			YY1AP1	POU2F2		HMGA1
CDKN2A				RET		HMGA2
CDKN2C				RICTOR		HOXA5
CEBPB				ROBO1		IFNB1
Crtc1				RPS6KA3		IGF1
CSF1R				RPS6KB1		IL6
CYBB				RUNX2		ITGA5
DGUOK				SFRP2		JAG1
DLL1				SH3GL1		LARP1
E2F1				SMO		LIN28B

E2F3	SOST	LOXL2
EPHA5	SP1	MALT1
ERBB2	STAM2	MAP3K2
FKBP1B	TFF1	MCL1
FLOT2	TOB1	MTDH
FOS	VOPP1	MYC
FOSL1	VPS51	NAMPT
FOXP1	WASF3	NOS2
FUT8		NRAS
GALNT7		NRP1
GAS1		PHB
GDF5		PIK3C2A
GFRA3		PIK3CG
GRM7		PLAG1
HDAC1		PLOD2
HMGB1		PRDX3
HNF4A		PRKCD
HNF4G		PTEN
HOTAIR		PTGS2
IFNB1		PTPN13
IGF2BP3		RB1
IL6R		RCBTB1
IMPA1		SERBP1
IMPDH2		SMAD1
INHBB		SMAD4
JAG1		ST3GAL6
KCNH1		ST8SIA4
KCNH2		STRADB
KDM4A		TDG
KIT		TET2
KLB		TRPC6
KLF12		WEE1
KLF4		ZCCHC11
L1CAM		
LDHA		
LEF1		
MAGEA12		
MAGEA2		
MAGEA3		
MAGEA6		
MAP2K1		
MAP3K9		
MDM4		
MET		
MTA2		
MYB		
MYC		
MYCN		

NAMPT
NANOG
NLRC5
NOTCH1
NOTCH2
NR4A2
NUMB
PAM
PCBP2
PDGFRA
PDGFRB
PEA15
PIK3CG
POU5F1
PPARA
PPP1CC
PPP1R10
PRKD1
RAD51
RBP2
RCAN1
RICTOR
SIRT1
SIRT6
SIRT7
SMAD4
SNAI1
SOX2
SPI1
SRC
STMN1
STX1A
SYT1
TCF7
TGIF2
TP53
TREM2
ULBP2
VAMP2
VEGFA
WNT1
YY1
ZAP70

miR-130a-3p	miR-16-5p	miR-197-3p	miR-409-3p	miR-27a-3p	miR-30a-5p	miR-331-3p
0.63	0.44	0.42	0.28	0.97	0.65	0.28
0.25	0.30	0.32	0.42	0.12	0.15	0.33
Acvr1	ACVR2A	ACVR2A	AKT1	ABCA1	ABL1	DOHH
APP	ADORA2A	ADORA2A	ANG	APC	ATF1	E2F1
ATG2B	AKT3	AKT3	CTNND1	BTG2	AVEN	ERBB2
ATXN1	APP	APP	ELF2	CCND1	BCL11A	FHIT
CSF1	ARHGDIA	ARHGDIA	FGA	CDC27	BCL9	HOTAIR
DICER1	ARL2	ARL2	FGB	DKK2	BDNF	ING5
DLL4	AXIN2	AXIN2	FGG	DPYD	BECN1	NRP2
ESR1	BACE1	BACE1	FRAT1	EGFR	CBX3	PHLPP1
GJA1	BCL2	BCL2	GAB1	FBXW7	CCNE2	
HOXA10	BDNF	BDNF	IFNG	FOXO1	CD99	
HOXA5	BIRC5	BIRC5	MET	FSTL1	CDH1	
IFITM1	BMI1	BMI1	MGMT	GATA2	DNMT1	
IL18	BRCA1	BRCA1	NLK	GATA3	DTL	
KLF4	CADM1	CADM1	PHF10	GRB2	ERG	
MAFB	CAPRIN1	CAPRIN1	RDX	GSK3B	ESR2	
MAP3K12	CCND1	CCND1	RECK	HIF1A	EYA2	
MECP2	CCND2	CCND2	RSU1	HIPK2	FOXD1	
MEOX2	CCND3	CCND3	STAG2	HMGCR	FOXL2	
MYC	CCNE1	CCNE1	UGT2B17	HOXA10	HCC	
PDGFRA	CCNT2	CCNT2	ZEB1	IFNG	HNF4G	
PPARA	CDK6	CDK6		IFNR	HSPA5	
PPARG	CDS2	CDS2		IGF1	IL21R	
PPARGC1A	CHEK1	CHEK1		KRAS	ITGB3	
PTEN	CHUK	CHUK		LAMP2	KLF9	
RAB5A	CLDN2	CLDN2		LDLR	LOX	
RUNX3	FGF2	FGF2		MAP2K4	MBNL1	
SLAIN1	FGFR1	FGFR1		MAPK14	MBNL2	
SMAD4	GLS2	GLS2		MET	MBNL3	
TAC1	HDGF	HDGF		MRC1	MTDH	
TGFB1	HGF	HGF		MYT1	NCAM1	
TGFBR2	HMGA1	HMGA1		NFE2L2	NEDD4L	
TNF	HMGA2	HMGA2		PAX3	NEUROD1	
XIAP	IFNG	IFNG		PDS5B	NOTCH1	
	IGF1R	IGF1R		PHB	NT5E	
	IL12B	IL12B		PHLPP2	PIK3CD	
	KDR	KDR		PIK3CG	PIK3R2	
	KRAS	KRAS		PINK1	PRDM1	
	MAP7	MAP7		PLAG1	PRRT2	
	METTL13	METTL13		PPARG	RAB38	
	MTOR	MTOR		PRKAA2	RPA1	
	MYB	MYB		PSAP	RUNX2	

NCOR2	NCOR2	RARA	SEPT7
NCSTN	NCSTN	RXRA	SKP2
OPRM1	OPRM1	SEMA6A	SMAD1
PIM1	PIM1	SEMA7A	SNAI1
PPM1D	PPM1D	SFRP1	SOX4
PRDM4	PRDM4	SIGLEC1	TAB3
PTGS2	PTGS2	SLC6A8	TET1
PURA	PURA	SLC7A11	TNRC6A
RAF1	RAF1	SMAD2	TP53
RECK	RECK	SMAD4	TRAF3IP2
RICTOR	RICTOR	SMAD5	TUBB4B
RPS6KB1	RPS6KB1	SP1	UBE3C
SLC6A4	SLC6A4	SP3	VIM
SOCS3	SOCS3	SP4	
SOX5	SOX5	SPRY2	
SOX6	SOX6	TFPI	
TP53	TP53	THRB	
TPPP3	TPPP3	TP53	
UCA1	UCA1	TRIM27	
UNG	UNG	WDR77	
VEGFA	VEGFA	WEE1	
WEE1	WEE1	WNK1	
WNT3A	WNT3A	YAP1	
WNT4	WNT4	YWHAZ	
YAP1	YAP1	ZBTB10	
ZYX	ZYX		

miR-376a-3p	miR-376c-3p	miR-210-3p	miR-106b-5p	miR-224-5p	miR-29c-3p	miR-335-5p
0.20	0.14	0.16	0.33	0.54	0.44	0.09
0.47	0.59	0.51	0.23	0.12	0.15	0.68
ACVR1C	ACVR1C	AIFM3	APC	AP2M1	ADAM12	ARPC5L
AGO2	BCL2	ALDH5A1	APP	API5	AKT2	BCL2L2
ATG4C	BMI1	ATG7	ATG16L1	APLN	AKT3	BIRC5
CASP8	GRB2	BDNF	BCL2L11	BCL2	BACE1	BRCA1
CDK2	IGF1R	BNIP3	CASP7	CASP3	BCL2	CRKL
IGF1R	NR5A2	BTK	CASP8	CASP7	CCND2	CXCR4
KLF15	RUNX2	CASP8AP2	CCND1	CDC42	CD274	DAAM2
MEPE	TGFA	COL4A2	CCND2	CDH1	CDC42	DKK1
PIK3R1	TGFBR1	CPEB2	CDKN1A	CXCR4	CDK6	EPN2
SLC16A1	UGT2B15	DDAH1	CYBB	DIO1	CNOT6	FMN2
SRSF11	UGT2B17	DIMT1	DAB2	DPYSL2	COL10A1	FMNL3
TTK		E2F3	E2F1	EDNRA	COL15A1	FOXA2
		EFNA3	E2F3	EYA4	COL1A1	GRM4
		EHD2	E2F5	FOSB	COL1A2	ID4
		FGFRL1	EOMES	GSK3B	COL21A1	IGF1R
		FOXN3	FAM129A	HOXD10	COL3A1	LRG1
		FOXP3	FYN	KLK10	COL4A1	MAPK1
		GPD1L	HIF1A	KRAS	COL4A2	MERTK
		HIF1A	ITCH	MBD2	COL5A2	MYC
		HIF3A	JAK1	MTOR	COL7A1	PARP1
		HOXA1	KAT2B	NCOA6	CREB5	PLAUR
		HOXA9	MAPK9	NIT1	CTNND1	POU5F1
		HSD17B1	MFN2	PAK2	DNMT3A	PTPRN2
		IGFBP3	MMP2	PEBP1	DNMT3B	RASA1
		INPP5A	PKD2	PHLPP1	FBN1	RB1
		ISCU	PRRX1	PHLPP2	FGA	ROCK1
		KCMF1	PTEN	PTX3	FGB	RUNX2
		LDHA	PTENP1	RAC1	FGG	SIAH2
		LDHB	PURA	RASSF8	FRAT2	SOX17
		MCM3	RB1	SERPINF2	FZD4	SOX4
		MNT	RBL1	SMAD4	FZD5	SP1
		MRE11	RBL2	TCEAL1	GAPDH	TCEAL9
		NCAM1	RHOC	TPD52	HMGCR	TFF2
		NDUFA4	RUNX3	TRIB1	IGFBP1	TNC
		NPTX1	SETD2		ITGA6	TRIM29
		P4HB	SLC2A4		ITGB1	TUG1
		PIM1	SMAD7		KLF4	UBE2F
		PLK1	STAT3		LAMC1	ZEB2
		PTBP3	TCEAL1		LAMC2	
		PTPN1	TNFRSF10A		LOX	
		PTPN2	TNFSF11		LRP6	

RAD52	TRIM8	MCL1
SDHD	TWIST1	MMP15
SH3BGRL	VEGFA	MMP2
STMN1	WEE1	MMP24
TFRC	ZBTB4	MYCN
THSD7A		NASP
TP53I11		PDGFRB
TWIST1		PER1
VMP1		PHLDB2
XIST		PPP1R13B
XPA		PTEN
		RCC2
		RFX7
		SERPINH1
		SIRT1
		SP1
		SPARC
		SRSF10
		TARBP1
		TDG
		TET2
		TFAP2C
		TGIF2
		TIAM1
		VEGFA
		WNT4

miR-31-3p	miR-146a-5p	miR-10a-5p	miR-27b-3p	miR-193a-5p	miR-149-5p	miR-30e-3p
0.34	0.00	0.25	0.35	0.16	0.11	0.11
0.18	16.38	0.21	0.09	0.19	0.23	0.21
E2F2	BCLAF1	ACTG1	ABCA1	ERBB2	BBC3	NFKBIA
NECTIN4	BRCA1	BCL2L11	ADORA2B	IGF2BP1	FASLG	
RHOA	BRCA2	BCL6	CCNA2	ING5	FGF21	
SDHA	CARD10	BDNF	CCNG1	MTOR	FGFR1	
	CASP7	BTRC	CCNT1	NLN	FOXM1	
	CCL5	CHL1	CDH11	PIK3R3	GIT1	
	CCNA2	EPHA4	CDH5	SRR	GPC1	
	CCND1	GP1BA	CREB1	TFAP2A	IL6	
	CCND2	HOXA1	CRISP2	TP73	MTHFR	
	CD40LG	MAP3K7	CX3CL1	WT1	MYD88	
	CD80	MAPK8IP1	CYP1B1		PPM1F	
	CDKN1A	MMP14	CYP3A4		PTGER2	
	CDKN3	NCOR2	DPYD		SP1	
	CFH	NOD2	EDNRA		ZBTB2	
	CNOT6L	PIK3CG	EGFR			
	COPS8	PTEN	EYA4			
	COX2	SERPINE1	FOXJ3			
	CPM	SRSF1	FOXO1			
	CXCL12	TRA2B	FZD7			
	CXCL8	USF2	HIP1R			
	CXCR4		HMGB3			
	DUSP1		KHSRP			
	EGFR		LDLR			
	ELAVL1		MET			
	ERBB4		MFF			
	FADD		MMP13			
	FAF1		NOTCH1			
	FANCM		NR2F2			
	FAS		NR5A2			
	HOXD10		OSBPL6			
	ICAM1		PAX3			
	IL6		PAX7			
	IRAK1		PHB			
	IRAK2		PINK1			
	IS2		PLK2			
	KIF22		PPARG			
	L1CAM		PSAP			
	LAMC2		RET			
	LFNG		ROR1			
	LRP2		RUNX1			
	MIF		SEMA6A			

MTA2	SHC1
NFAT5	SMAD2
NFKB1	ST14
NOS1	TGFBR1
NOTCH1	THBS1
NOTCH2	THBS2
NUMB	TRAPPC2B
PA2G4	UCA1
PLAUR	VDR
PRKCE	VEGFC
PTGES2	WEE1
PTGS2	
RAC1	
RARB	
RHO	
RHOA	
RNF11	
ROCK1	
SIKE1	
SLPI	
SMAD2	
SMAD4	
SMN1	
SOS1	
SOX2	
STAT1	
TGFB1	
TLR2	
TLR4	
TRAF6	
UHRF1	
WASF2	

miR-181a-5p	miR-365a-3p	miR-30a-3p	miR-296-5p	miR-22-3p	miR-25-3p	miR-342-3p
0.11	0.18	0.10	0.16	0.16	0.20	0.05
0.21	0.12	0.23	0.12	0.12	0.08	0.32
ABCG2	ACVR1	BECN1	BBC3	ACLY	ATP2A2	BIRC6
AHR	BAX	CDK6	CASP8	ACVR1C	BCL2L11	BMP7
ATG5	BCL2	CYR61	CDX1	AKT1	CCL26	CTBP2
ATM	CCND1	MECP2	DLL4	ARPC5	CDH1	DNMT1
BAX	CDC25A	NFATC3	ELAVL1	BDNF	CDKN1C	E2F1
BCL2	IL6	NOTCH1	FGFR1	BMP6	CPEB1	GEMIN4
BCL2L11	KRAS	RUNX2	HGS	BMP7	CYP2B6	ID4
CD4	MAX	SLC7A6	HMGA1	BMPR1B	DHFR	IKBKG
CDKN1A	PAX6	THBS1	IKBKE	BSG	DSC2	MTDH
CDKN1B	SHC1	TMEM2	KDR	BTG1	ERBB2	SREBF1
CDX2		VEZT	NGFR	CCNA2	EZH2	SREBF2
CEBPA		XBP1	NOTCH1	CD151	FBXW7	TAB2
COL16A1			PIN1	CDKN1A	HAND2	TAB3
CTDSPL			PLK1	CSF1R	KAT2B	
CTNNB1			S100B	CXCR2	KLF4	
DDIT4			SCRIB	CYR61	LATS2	
DDX3X			VEGFA	ERBB2	MAP2K4	
DUSP5			WNK4	ERBB3	MDM2	
DUSP6				ESR1	PRMT5	
E2F5				HDAC4	PTEN	
EGR1				HDAC6	RECK	
FOS				HIF1A	REV3L	
GATA6				HMGB1	RGS3	
GPD1L				HTR2C	SEMA4C	
GPR78				IRF5	SMAD7	
HIPK2				LGALS1	TCEAL1	
HRAS				LGALS9	TNFSF10	
IFNG				MALAT1	TP53	
INPP4B				MAOA	WDR4	
KAT2B				MECOM		
KLF6				MMP14		
KRAS				MTA1		
MAP2K1				MTDH		
MAPK1				MTHFR		
MCL1				MYCBP		
MEG3				NCOA1		
MTMR3				NET1		
NLK				NTRK2		
NOTCH1				PLK1		
NRAS				PPARA		
PBX3				PPM1K		

PGR
PHLPP2
PLAG1
PPP3CA
PRAP1
PRKCD
PRKN
PROX1
PTEN
PTPN11
PTPN22
RALA
RAP1B
RASSF1
RASSF6
RGS16
RGS5
RNF2
RUNX1
SAMHD1
SIRT1
STAT3
TCF4
TERT
TGFBR1
TGFBRAP1
TIMP1
TUSC3
TWIST1
VEGFA
WIF1
XIAP
ZNF763

PTEN
PTMS
RAB5B
RCOR1
RGS2
SIRT1
SLC2A1
SNAI1
SP1
TACC1
TCEAL1
TCF7
TET2
TFRC
TIAM1
UBR5
WNT1

miR-28-3p	miR-26b-5p	miR-532-5p	miR-28-5p	miR-483-5	miR-93-5p	let-7a-5p
0.10	0.14	0.08	0.14	0.07	0.06	0.19
0.12	0.09	0.12	0.06	0.10	0.10	0.03
STAT5B	ABCA1	CXCL2	BAG1	ALCAM	ABCA1	AGO1
TP53	ARL4C	FASN	CDKN1A	CKB	ANG	AGO4
	CCNE1	NKD1	E2F6	FAM160B2	ATG16L1	APP
	CDK6	RUNX3	IGF1	MAPK3	CDKN1A	ARG2
	CHORDC1	SYK	IL34	NOTCH3	CERS2	AURKB
	COL1A2	TERT	MAD2L1	RHOA	CXCL8	CASP3
	COX2	TRAPPC2B	MAPK1	SRF	DAB2	CASP8
	CTGF		MPL		E2F1	CASP9
	EPHA2		N4BP1		FOXA1	CCND2
	EZH2		OTUB1		FOXO3	CCR7
	FH		RAP1B		ITGB8	CDC34
	GATA4		STAT5B		KAT2B	CDK6
	HAS2		TEX261		LATS2	CDKN1A
	HGF		TP53		MAPK9	DICER1
	IGF1				MMP3	E2F1
	IGF1R				PDCD4	E2F2
	JAG1				PHLPP2	EGFR
	KPNA2				PTEN	EWSR1
	LARP1				PTENP1	EZH2
	MIEN1				PURA	FOXA1
	NAMPT				RAB11FIP1	HAS2
	NR2C2				RHOC	HMGA1
	PDE4A				RPS6KA4	HMGA2
	PLOD2				SLC2A4	HRAS
	PTEN				SMAD7	IGF2
	PTGS2				STK11	IGF2BP1
	RB1				TGFBR2	IL6
	SMAD1				TP53INP1	ITGB3
	ST8SIA4				TUSC2	KRAS
	TAB1				VEGFA	LIN28A
	TLR4				ZBTB4	LIN28B
	TRAF5				ZNRF3	MAP4K4
	ULK2					MPL
	USP9X					MYC
						NF2
						NKIRAS2
						NR1I2
						NRAS
						PAK1
						PARP1
						PKM

PRDM1
RAB40C
RAVER2
RRM2
STAT3
TGFBR3
TMED7
TNFAIP3
TNFRSF10B
TRIM71
UHRF1
UHRF2
VDR
WNT1

miR-130b-3p	miR-143-3p	miR-148a-3p	miR-361-5p	miR-146b-5p	miR-34a-3p	miR-138-5p
0.08	0.11	0.09	0.09	0.03	0.08	0.19
0.08	0.05	0.05	0.05	0.15	0.05	0.02
CCDC6	AKT1	ACVR1	CXCR6	CDKN1A	ATP5S	ADGRA2
CCNA2	AKT2	ALCAM	SND1	EGFR	AXIN2	AKT1
CMPK1	BAG3	BAX	STAT6	ERBB4	BCL2	ARHGEF3
CSF1	BCL2	BCL2	TWIST1	HNRNPD	CCND1	BAG1
CYLD	BRAF	BCL2L11	VEGFA	IL6	CD274	BCL11A
CYP2C9	CD44	CCKBR		IRAK1	CDK6	BLCAP
DICER1	COL1A1	CDC25B		KIT	CTNNB1	CASP3
DLL1	COL3A1	CDKN1B		MALAT1	FRAT1	CCND1
ERBB2	CTGF	DNMT1		MMP16	LDHA	CCND3
FMR1	CYP2C9	DNMT3B		NFKB1	MET	CD274
IGF1	DDX6	ERRFI1		PAX8	MYC	CDH1
IRF1	DNMT3A	HLA-G		PDGFRA	NCOR1	CYTOR
ITGB1	DTNB	IKBKB		RARB	NCOR2	EED
LDLR	FAM83F	INO80		SLC5A5	PDE4B	EID1
MMP2	FHIT	IRS1		TLR4	SIRT1	EIF4EBP1
MST1	FNDC3B	ITGA5		TRAF6	SMAD4	EZH2
NKD2	FSCN1	ITGB8		UHRF1	TGIF2	FERMT2
NR3C1	GABARAPL1	MAFB		ZNRF3	XIAP	FOSL1
PDGFRA	HK2	MAP3K4				FOXC1
PPARA	HNF4A	MAP3K9				GNAI2
PPARG	HRAS	MET				H2AFX
PPARGC1A	IGF1R	MMP7				HIF1A
PTEN	IL13RA1	NR1I2				IGF1R
RUNX3	ITGB1	NRP1				KDM5C
SAV1	ITGB4	PBXIP1				LCN2
SCD	JAG1	PDIA3				MAP3K11
SMAD4	KLF5	QKI				MXD1
SNAI3	KRAS	ROCK1				NFKB1
STAT3	LIMK1	RPS6KA5				PTK2
TP53INP1	MACC1	RUNX3				RARA
UCP1	MAPK7	S1PR1				RELN
UVRAG	MDM2	SERPINE1				RHOC
ZBTB4	MMP13	SMAD2				RMND5A
ZEB1	MMP14	STAT3				ROCK2
	MMP2	TGFB2				S100A1
	MMP9	TGIF2				SENP1
	MYO6	TMED7				SIRT1
	NFATC1	USP4				SLC45A3
	NFKB2	VAV2				SNAI2
	NR2C2	WNT1				SOX4
	OSBPL8	WNT10B				SOX9

PTGS2	SUZ12
RREB1	TERT
SDC1	TWIST2
SERPINE1	VIM
TLR2	YAP1
TNF	ZEB2

miR-186-5p	miR-663b	miR-23a-3p	miR-495-3p	miR-19a-3p	miR-654-5p	miR-886-5p
0.03	0.13	0.10	0.04	0.03	0.04	0.03
0.12	0.03	0.03	0.07	0.08	0.06	0.08
ABCB1	EEF1A2	APAF1	ABCB1	ABCA1	EPSTI1	
AKAP12	IGF2	ATAT1	AKT1	ADRB1		
CSNK2A1		CDH1	ATP7A	AKT1		
FGF2		CHUK	BMI1	ALOX5		
FOXO1		CXCL12	CCL2	ATXN1		
GJA1		CXCL8	FOXC1	BCL2L11		
HIF1A		FANCG	HMGA2	BMPR2		
MAP3K2		FAS	HSPA5	CCND1		
NCSTN		FOXA1	MAT1A	CD22		
P2RX7		FOXO3	MEIS1	CUL5		
PAK5		FZD5	MTA3	DNMT1		
PPM1B		G6PC	PBX3	DPYSL2		
PTTG1		GJA1	PTP4A3	ERBB4		
PVT1		GLS	RUNX3	ESR1		
RELA		HES1	SMR3B	FOXP1		
SETD2		HIP1R	SOX9	HOXA5		
TWIST1		HMGB2	TBC1D9	IL10		
VEGFA		HMGN2		IMPDH1		
XIAP		HNF1B		KAT2B		
		HOXB4		KIT		
		HSP90AA1		MAP3K5		
		IL6R		MECP2		
		IRF1		MEF2D		
		KLF3		MSMO1		
		LAMP1		MTUS1		
		LDHA		MXD1		
		LDHB		MYCN		
		LPAR1		NPEPL1		
		LRP5		NR4A2		
		MEF2C		PHLPP1		
		MT2A		PIK3CA		
		MYH1		PKNOX1		
		MYH2		PMEPA1		
		MYH4		PRMT5		
		NEK6		PSAP		
		POU4F2		PTEN		
		PPARGC1A		RAB13		
		PPP2R5E		RAB14		
		PTEN		RHOB		
		PTPN11		SIVA1		
		RGS5		SMAD4		

SMAD3	SOCS1
SMAD5	SOCS3
SPRY2	SUZ12
ST7L	TF
STAT3	TGFBR2
TERF2	TLR2
TMEM64	TLR7
TOP1	TNF
TSC1	TNFAIP3
XIAP	TNFRSF12A
	TP53INP1
	TUSC2
	VPS4B
	ZBTB4

miR-134-5p	miR-744-5p	miR-301a-3p	miR-29b-3p	let-7c-5p	miR-195-5p	miR-1260a
0.02	0.03	0.02	0.09	0.18	0.12	0.17
0.11	0.08	0.08	0.01	0.01	0.00	0.00
ABCC1	ARHGAP5	BCL2L11	ADAM12	AGO1	ARL2	
ANGPTL4	EEF1A2	CDC14A	AKT2	BCL2L1	ATG14	
ERBB2	GSK3B	MAP3K5	AKT3	CASP3	Bace1	
FOXM1	MYC	MEOX2	ANGPTL4	CDC25A	BCL2	
GOLPH3	NKD1	NKRF	AQP4	CEBPB	BCL2L2	
ITGB1	SFRP1	PTEN	BACE1	COPS6	BIRC5	
KRAS	TLE3	RUNX3	BCL2	COPS8	BTRC	
MAGI2		SERPINE1	BMP1	DICER1	CAB39	
NANOG		SMAD4	CCND2	GPS1	CBX4	
OPRM1		SNIP1	CDC42	HMGA2	CCL4	
PAK2		TIMP2	CDK6	HSPA4	CCND1	
PUM2		UVRAG	COL10A1	IGF1R	CCND3	
RAB27A			COL1A1	IL10	CCNE1	
STAT5B			COL3A1	IL6	CDC25A	
VEGFA			COL4A1	ITGB3	CDC42	
VIM			COL4A2	MAP4K3	CDK4	
ZDHHC9			COL5A2	MPL	CDK6	
			COL5A3	MTOR	CDK8	
			CTNNBIP1	MYC	CHEK1	
			DNAJB11	NRAS	CHUK	
			DNMT1	NUMB	DICER1	
			DNMT3A	PBX2	E2F3	
			DNMT3B	RICTOR	ELN	
			DUSP2	STAT3	FASN	
			ELAVL1	TGFBR1	FGF2	
			ESR1	TNFRSF10B	HMGA1	
			FBN1	TRIB2	INSR	
			FGA	TRIM71	KDR	
			FGB		KIAA0100	
			FGG		MBD1	
			FOS		MYB	
			GATA3		NKD1	
			GRN		RAF1	
			GSK3B		RET	
			HDAC4		RPS6KB1	
			HMGA2		RUNX2	
			HMGCR		SLC2A3	
			IFNG		SMAD7	
			IL32		TAB3	
			IMPDH1		TBCCD1	
			ITGA6		VEGFA	

ITGB1
KDM2A
KIF1B
LAMC1
LAMC2
LASP1
LOX
LOXL2
LOXL4
LRP6
MCL1
MEN1
MMP15
MMP2
MMP24
MMP9
MYC
MYCN
NASP
NCOA3
NID1
NKIRAS2
PATZ1
PDGFA
PDGFB
PDGFC
PDGFRA
PDGFRB
PER1
PIK3CG
PIK3R1
PPIC
PPP1R13B
PTEN
RAX
S100B
SERPINH1
SFPQ
SNAI3
SP1
SPARC
STAT3
TBX21
TCL1A
TDG
TET1
TET2
TGFB1
WEE1
WNT7A
YAP1

TGFB2
TGFB3
TNFAIP3
VEGFA

miR-30d-3p

0.11
0.00

ADAM12

Supplementary Table 5

First quartile hASC-EVs miRNAs targets	First quartile hAMSC-EVs miRNAs targets
ABCA1	ABCA1
ABCB1	ABCB1
ABCB9	ABCB9
ABCC1	ABCC1
ABCG2	ABCG2
ABHD17C	ABHD17C
ABL1	ABL1
ABL2	ABL2
ABRACL	ABRACL
ABTB1	ABTB1
ACKR3	ACKR3
ACLY	ACLY
ACSL1	ACSL1
ACSL4	ACSL4
ACTB	ACTB
ACTG1	ACTG1
ACTN1	ACTN1
ACVR1	Acvr1
ACVR1B	ACVR1B
ACVR1C	ACVR1C
ACVR2A	ACVR2A
ADAM12	ADAM12
ADAM17	ADAM17
ADAM1A	ADAM1A
ADAMTS6	ADAMTS6
ADAMTS9	ADAMTS9
ADAR	ADAR
ADD3	ADD3
ADGRA2	ADORA2A
ADORA2A	ADORA2B
ADORA2B	ADRB1
AGO1	AGO2
AGO2	AGPAT2
AGO4	AGTR1
AGPAT2	AHR
AGTR1	AHRR
AHR	AIFM3
AHRR	AIP
AIFM3	AKAP12
AIP	AKR1B10
AKR1B10	AKR1C2
AKR1C2	AKT1
AKT1	AKT2
AKT2	AKT3

AKT3	ALCAM
ALCAM	ALDH3A1
ALDH3A1	ALDH5A1
ALDH5A1	ALOX5
ALOX5	ALPK2
ALPK2	ALPPL2
ALPPL2	AMACR
AMACR	ANG
ANG	ANGPT2
ANGPT2	ANGPTL4
ANGPTL4	ANK3
ANK3	ANKH
ANKH	ANKRD46
ANKRD46	ANP32A
ANP32A	ANXA1
ANXA1	AP1G1
AP1G1	AP2M1
AP2M1	APAF1
APAF1	APC
APC	APH1A
APH1A	API5
API5	APLN
APLN	APOE
APOE	APP
APP	AQP1
AQP1	AQP4
AQP4	AR
AR	ARF1
ARF1	ARF4
ARF4	ARF6
ARF6	ARHGAP12
ARG2	ARHGAP19
ARHGAP12	ARHGAP32
ARHGAP19	ARHGAP5
ARHGAP32	ARHGDIA
ARHGDIA	ARHGDIB
ARHGDIB	ARID1A
ARHGEF3	ARID3A
ARID1A	ARID3B
ARID3A	ARID4B
ARID3B	ARIH2
ARID4B	ARL2
ARIH2	ARL4C
ARL2	ARL6IP5
ARL4C	ARNT
ARL6IP5	ARPC5
ARNT	ARPC5L
ARPC5	ARPP19

ARPC5L	ARTN
ARPP19	ASF1B
ARTN	ASZ1
ASF1B	ATF1
ASZ1	ATF4
ATAT1	ATG12
ATF1	ATG14
ATF4	ATG16L1
ATG12	ATG2B
ATG14	ATG4A
ATG16L1	ATG4B
ATG2B	ATG4C
ATG4A	ATG4D
ATG4B	ATG5
ATG4C	ATG7
ATG4D	ATG9A
ATG5	ATM
ATG7	ATP2A2
ATG9A	ATP5S
ATM	ATP7A
ATP2A2	ATXN1
ATP5S	AURKB
ATXN1	AVEN
AURKB	AXIN2
AVEN	AXL
AXIN2	BACE1
AXL	BAG1
BACE1	BAG4
BAG1	BAG5
BAG3	BAK1
BAG4	BAMBI
BAG5	BANP
BAK1	BAP1
BAMBI	BASP1
BANP	BAX
BAP1	BBC3
BASP1	BCAR1
BAX	BCL10
BBC3	BCL11A
BCAR1	BCL2
BCL10	BCL2L11
BCL11A	BCL2L2
BCL2	BCL3
BCL2L1	BCL6
BCL2L11	BCL7A
BCL2L2	BCL9
BCL3	BCLAF1
BCL6	BDNF

BCL7A
BCL9
BDNF
BECN1
BIRC5
BIRC6
BLCAP
BMF
BMI1
BMP1
BMP2
BMP6
BMP7
BMPR1B
BMPR2
BNIP2
BNIP3
BNIP3L
BRAF
BRAP
BRCA1
BRCA2
BSG
BTG1
BTG2
BTK
BTRC
C11orf65
C1QTNF9
CAB39
CACNA1C
CACNB3
CADM1
CALCR
CAMK1D
CAMK2D
CAPNS1
CAPRIN1
CARD10
CARM1
CASC2
CASP3
CASP7
CASP8
CASP8AP2
CASP9
CASR
CAT

BECN1
BIRC5
BIRC6
BMF
BMI1
BMP2
BMP6
BMP7
BMPR1B
BMPR2
BNIP2
BNIP3
BNIP3L
BRAF
BRAP
BRCA1
BRCA2
BSG
BTG1
BTG2
BTK
BTRC
C11orf65
C1QTNF9
CACNA1C
CACNB3
CADM1
CALCR
CAMK1D
CAMK2D
CAPNS1
CAPRIN1
CARD10
CARM1
CASC2
CASP3
CASP7
CASP8
CASP8AP2
CASR
CAT
CAV2
CBFB
CBX3
CBX4
CCDC43
CCDC6
CCKBR

CAV2	CCL1
CBFB	CCL2
CBX3	CCL20
CBX4	CCL22
CCDC43	CCL26
CCDC6	CCL3
CCKBR	CCL5
CCL1	CCL8
CCL20	CCNA2
CCL22	CCNB1
CCL26	CCND1
CCL3	CCND2
CCL4	CCND3
CCL5	CCNE1
CCL8	CCNE2
CCNA2	CCNG1
CCNB1	CCNJ
CCND1	CCNT1
CCND2	CCNT2
CCND3	CCR1
CCNE1	CD151
CCNE2	CD22
CCNG1	CD24
CCNJ	CD274
CCNT1	CD276
CCNT2	CD28
CCR1	CD4
CCR7	CD40
CD151	CD40LG
CD24	CD44
CD274	CD69
CD276	CD80
CD28	CD93
CD4	CD99
CD40	CDC14A
CD44	CDC25A
CD69	CDC27
CD93	CDC42
CD99	CDC6
CDC25A	CDC7
CDC25B	CDH1
CDC27	CDH11
CDC34	CDH2
CDC42	CDH5
CDC6	CDK1
CDC7	CDK2
CDH1	CDK2AP1
CDH11	CDK4

CDH2
CDH5
CDK1
CDK2
CDK2AP1
CDK4
CDK6
CDK7
CDK8
CDK9
CDKN1A
CDKN1B
CDKN1C
CDKN2A
CDKN2C
CDKN2D
CDS2
CDX1
CDX2
CEACAM6
CEBPA
CEBPB
CEP19
CERS2
CFTR
CGN
CHD1
CHEK1
CHL1
CHORDC1
CHUK
CKS2
CLDN1
CLDN2
CLINT1
CLOCK
CLTC
CLU
CMPK1
CNOT6
COL10A1
COL15A1
COL16A1
COL1A1
COL1A2
COL21A1
COL3A1
COL4A1

CDK6
CDK7
CDK8
CDK9
CDKN1A
CDKN1B
CDKN1C
CDKN2A
CDKN2C
CDKN2D
CDKN3
CDS2
CDX1
CDX2
CEACAM6
CEBPA
CEBPB
CEP19
CERS2
CFH
CFTR
CGN
CHD1
CHEK1
CHL1
CHORDC1
CHUK
CKB
CKS2
CLDN1
CLDN2
CLINT1
CLOCK
CLTC
CLU
CMPK1
CNOT6
CNOT6L
COL10A1
COL15A1
COL16A1
COL1A1
COL1A2
COL21A1
COL3A1
COL4A1
COL4A2
COL5A1

COL4A2
COL5A1
COL5A2
COL5A3
COL7A1
COPS5
COPS6
COPS8
CORO1A
COX2
CPD
CPEB1
CPEB2
CPEB3
CPEB4
CREB1
CREB5
CREBZF
CREG1
CRIM1
CRISP2
CRK
CRKL
CRNDE
Crtc1
CSF1
CSF1R
CSNK2A1
CTCF
CTDSP2
CTDSPL
CTGF
CTHRC1
CTNNB1
CTNNBIP1
CTNND1
CUL2
CUL5
CX3CL1
CXCL12
CXCL2
CXCL8
CXCR2
CXCR4
CXCR6
CYB5A
CYBB
CYLD

COL5A2
COL7A1
COPS5
COPS8
CORO1A
COX2
CPD
CPEB1
CPEB2
CPEB3
CPEB4
CPM
CREB1
CREB5
CREBZF
CREG1
CRIM1
CRISP2
CRK
CRKL
CRNDE
Crtc1
CSF1
CSF1R
CSNK2A1
CTBP2
CTCF
CTDSP2
CTDSPL
CTGF
CTHRC1
CTNNB1
CTNND1
CUL2
CUL5
CX3CL1
CXCL12
CXCL2
CXCL8
CXCR2
CXCR4
CYB5A
CYBB
CYLD
CYP11B2
CYP19A1
CYP1B1
CYP24A1

CYP11B2
CYP19A1
CYP1B1
CYP24A1
CYP2B6
CYP2C19
CYP2C9
CYP3A4
CYP7B1
CYR61
CYTOR
DAAM2
DAB2
DACT3
DAPK3
DAXX
DDAH1
DDC
DDIT4
DDX17
DDX3X
DDX6
DEDD
DERL1
DFFA
DGAT1
DGUOK
DHFR
DHFRP1
DICER1
DIMT1
DIO1
DIRAS3
DKK1
DKK2
DKK3
DLL1
DLL4
DMD
DNAJA4
DNAJB11
DNAJC27
DND1
DNM1L
DNMT1
DNMT3A
DNMT3B
DOCK1

CYP2B6
CYP2C19
CYP2C9
CYP3A4
CYP7B1
CYR61
DAAM2
DAB2
DACT3
DAPK3
DAXX
DDAH1
DDC
DDIT4
DDX17
DDX3X
DDX6
DEDD
DERL1
DFFA
DGAT1
DGUOK
DHFR
DHFRP1
DICER1
DIMT1
DIO1
DIRAS3
DKK1
DKK2
DKK3
DLL1
DLL4
DMD
DNAJA4
DNAJC27
DND1
DNM1L
DNMT1
DNMT3A
DNMT3B
DOCK1
DOCK4
DOCK5
DOCK7
DOHH
DPYD
DPYSL2

DOCK4
DOCK5
DOCK7
DOHH
DPYD
DPYSL2
DRAM2
DRD1
DSC2
DTD1
DTL
DTNB
DUSP10
DUSP2
DUSP4
DUSP5
DUSP6
DVL2
DYRK2
E2F1
E2F2
E2F3
E2F5
E2F6
EBP
ECT2
EDNRA
EED
EEF1A2
EFNA1
EFNA3
EGF
EGFR
EGLN1
EGLN3
EGR1
EGR2
EHD2
EID1
EIF2S1
EIF2S3
EIF4A2
EIF4E
EIF4EBP1
EIF5A2
ELAVL1
ELF2
ELN

DRAM2
DRD1
DSC2
DTD1
DTL
DUSP1
DUSP10
DUSP2
DUSP4
DUSP5
DUSP6
DVL2
DYRK2
E2F1
E2F2
E2F3
E2F5
E2F6
EBP
ECT2
EDNRA
EEF1A2
EFNA1
EFNA3
EGF
EGFR
EGLN1
EGLN3
EGR1
EGR2
EHD2
EIF2S1
EIF2S3
EIF4A2
EIF4E
EIF4EBP1
EIF5A2
ELAVL1
ELF2
ELN
EMSY
ENPEP
EOMES
EP300
EPAS1
EPB41L3
EPHA2
EPHA4

EMSY
ENPEP
EOMES
EP300
EPAS1
EPB41L3
EPHA2
EPHA4
EPHA5
EPN2
EPO
EPOR
ERBB2
ERBB3
ERCC1
ERG
ERRFI1
ESR1
ESR2
ESRRG
ETS1
ETV1
EWSR1
EYA2
EYA4
EZH2
F11R
FAF1
FAM129A
FAM3C
FAM45A
FAM83F
FANCG
FAS
FASLG
FASN
FASTK
FBN1
FBXO11
FBXO31
FBXO8
FBXW7
FEN1
FERMT2
FES
FGA
FGB
FGF11

EPHA5
EPN2
EPO
EPOR
EPSTI1
ERBB2
ERBB3
ERBB4
ERCC1
ERG
ESR1
ESR2
ESRRG
ETS1
ETV1
EYA2
EYA4
EZH2
F11R
FADD
FAF1
FAM129A
FAM160B2
FAM3C
FAM45A
FANCM
FAS
FASLG
FASN
FASTK
FBN1
FBXO11
FBXO31
FBXO8
FBXW7
FEN1
FES
FGA
FGB
FGF11
FGF2
FGF21
FGF9
FGFR1
FGFR2
FGFR3
FGFRL1
FGG

FGF2	FH
FGF21	FHIT
FGF9	FIS1
FGFR1	FKBP1B
FGFR2	FKBP5
FGFR3	FLI1
FGFRL1	FLOT2
FGG	FLT1
FH	FMN2
FHIT	FMNL3
FIS1	FMOD
FKBP1B	FMR1
FKBP5	FOS
FLI1	FOSB
FLOT2	FOSL1
FLT1	FOXA1
FMN2	FOXA2
FMNL3	FOXC1
FMOD	FOXD1
FMR1	FOXJ3
FNDC3B	FOXL2
FOS	FOXM1
FOSB	FOXN3
FOSL1	FOXO1
FOXA1	FOXO3
FOXA2	FOXP1
FOXC1	FOXP3
FOXD1	FRAT1
FOXJ3	FRAT2
FOXL2	FSCN1
FOXM1	FSTL1
FOXN3	FURIN
FOXO1	FUT4
FOXO3	FUT8
FOXP1	FXN
FOXP3	FYN
FRAT1	FZD3
FRAT2	FZD4
FSCN1	FZD5
FSTL1	FZD6
FURIN	FZD7
FUT4	GAB1
FUT8	GAB2
FXN	GALNT7
FYN	GAPDH
FZD3	GAS1
FZD4	GAS5
FZD5	GATA2

FZD6
FZD7
G6PC
GAB1
GAB2
GABARAPL1
GALNT7
GAPDH
GAS1
GAS5
GATA2
GATA3
GATA4
GATA6
GCM1
GDAP1
GDF5
GFRA3
GIT1
GJA1
GLI1
GLI2
GLS
GLS2
GLUL
GMFB
GNA13
GNAI1
GNAI2
GNAI3
GOLM1
GP1BA
GPC1
GPD1L
GPR137B
GPR78
GPR85
GPS1
GRB10
GRB2
GRIN2A
GRM4
GRM7
GRN
GSK3B
GSR
GSS
H2AFX

GATA3
GATA4
GATA6
GCM1
GDAP1
GDF5
GEMIN4
GFRA3
GIT1
GJA1
GLI1
GLI2
GLS2
GLUL
GMFB
GNA13
GNAI1
GNAI2
GNAI3
GOLM1
GOLPH3
GP1BA
GPC1
GPD1L
GPR137B
GPR78
GPR85
GRB10
GRB2
GRIN2A
GRM4
GRM7
GSK3B
GSR
GSS
H2AFX
HAND2
HAS2
HAX1
HBEGF
HBP1
HCC
HDAC1
HDAC11
HDAC2
HDAC4
HDAC6
HDGF

HAND2	HECTD2
HAS2	HGF
HAX1	HGS
HBEGF	HIF1A
HBP1	HIF1AN
HCC	HIF3A
HDAC1	HIP1R
HDAC11	HIPK1
HDAC2	HIPK2
HDAC4	HIPK3
HDAC6	HK2
HDGF	HLA-G
HECTD2	HLTF
HES1	HMGA1
HGF	HMGA2
HGS	HMGB1
HIF1A	HMGB3
HIF1AN	HMGCR
HIF3A	HMGXB4
HIP1R	HMOX1
HIPK1	HNF4A
HIPK2	HNF4G
HIPK3	HNRNPD
HK2	HNRNPK
HLA-G	HOTAIR
HLTF	HOTTIP
HMGA1	HOXA1
HMGA2	HOXA10
HMGB1	HOXA5
HMGB2	HOXA9
HMGB3	HOXB3
HMGCR	HOXB5
HMGN2	HOXC13
HMGXB4	HOXD10
HMOX1	HPGD
HNF1B	HRAS
HNF4A	HS3ST2
HNF4G	HSD17B1
HNRNPK	HSP90B1
HOTAIR	HSPA4
HOTTIP	HSPA5
HOXA1	HSPB2
HOXA10	HSPB6
HOXA5	HTR2C
HOXA9	ICAM1
HOXB3	ICAM2
HOXB4	ICOSLG
HOXB5	ID4

HOXC13
HOXD10
HPGD
HRAS
HS3ST2
HSD17B1
HSP90AA1
HSP90B1
HSPA4
HSPA5
HSPB2
HSPB6
HTR2C
ICAM1
ICAM2
ICOSLG
ID4
IDH1
IER2
IFITM1
IFNAR1
IFNB1
IFNG
IFNR
IGF1
IGF1R
IGF2
IGF2BP1
IGF2BP3
IGFBP1
IGFBP3
IKBKB
IKBKE
IKZF1
IKZF2
IKZF3
IKZF4
IL10
IL11
IL12A
IL12B
IL13RA1
IL18
IL1A
IL1B
IL21R
IL25
IL32
IDH1
IER2
IFITM1
IFNAR1
IFNB1
IFNG
IFNR
IGF1
IGF1R
IGF2
IGF2BP1
IGF2BP3
IGFBP1
IGFBP3
IKBKB
IKBKE
IKBKG
IKZF1
IKZF2
IKZF3
IKZF4
IL10
IL11
IL12A
IL12B
IL18
IL1A
IL1B
IL21R
IL25
IL34
IL4
IL6
IL6R
ILK
IMPA1
IMPDH1
IMPDH2
ING4
ING5
INHBB
INPP4B
INPP5A
INSIG1
IRAK1
IRAK2
IRAK4
IRF1

IL34	IRF2
IL4	IRF4
IL6	IRF5
IL6R	IRS1
ILK	IRS2
IMPA1	IS2
IMPDH1	ISCU
IMPDH2	ITCH
ING4	ITGA11
ING5	ITGA3
INHBB	ITGA5
INO80	ITGA6
INPP4B	ITGB1
INPP5A	ITGB3
INSIG1	ITGB8
INSR	ITIH5
IRAK1	JADE1
IRAK4	JAG1
IRF1	JAK1
IRF2	JAK2
IRF4	JAZF1
IRF5	JMY
IRS1	JPH2
IRS2	JPT1
ISCU	KAT2B
ITCH	KCMF1
ITGA11	KCNH1
ITGA3	KCNH2
ITGA5	KDM4A
ITGA6	KDM5B
ITGB1	KDR
ITGB3	KEAP1
ITGB4	KHSRP
ITGB8	KIF22
ITIH5	KIF26B
JADE1	KIT
JAG1	KITLG
JAK1	KLB
JAK2	KLC2
JAZF1	KLF12
JMY	KLF13
JPH2	KLF15
JPT1	KLF2
KAT2B	KLF4
KCMF1	KLF5
KCNH1	KLF6
KCNH2	KLF9
KDM2A	KLHL11

KDM4A	KLK10
KDM5B	KMT5A
KDM5C	KPNA2
KDR	KRAS
KEAP1	KREMEN1
KHSRP	KREMEN2
KIAA0100	L1CAM
KIF1B	LACTB
KIF26B	LAMB3
KIT	LAMC1
KITLG	LAMC2
KLB	LAMP2
KLC2	LARP1
KLF12	LASP1
KLF13	LATS2
KLF15	LDHA
KLF2	LDHB
KLF3	LDLR
KLF4	LEF1
KLF5	LFNG
KLF6	LGALS1
KLF9	LGALS9
KLHL11	LGR4
KLK10	LIF
KMT5A	LIFR
KPNA2	LIMK1
KRAS	LIN28A
KREMEN1	LIN28B
KREMEN2	LIPA
L1CAM	LMO2
LACTB	LOX
LAMB3	LOXL2
LAMC1	LPL
LAMC2	LRG1
LAMP1	LRP2
LAMP2	LRP6
LARP1	LRRC8A
LASP1	LRRFIP1
LATS2	LTF
LCN2	LYPLA2
LDHA	LZTS1
LDHB	MAD2L1
LDLR	MAFB
LEF1	MAGEA12
LGALS1	MAGEA2
LGALS9	MAGEA3
LGR4	MAGEA6
LIF	MAGI2

LIFR
LIMK1
LIN28A
LIN28B
LIPA
LMO2
LOX
LOXL2
LOXL4
LPAR1
LPL
LRG1
LRP5
LRP6
LRRC8A
LRRFIP1
LTF
LYPLA2
LZTS1
MACC1
MAD2L1
MAFB
MAGEA12
MAGEA2
MAGEA3
MAGEA6
MALAT1
MALT1
MAN1B1
MAOA
MAP2K1
MAP2K3
MAP2K4
MAP2K6
MAP3K11
MAP3K12
MAP3K2
MAP3K4
MAP3K5
MAP3K7
MAP3K9
MAP4K3
MAP4K4
MAP7
MAPK1
MAPK14
MAPK4
MAPK7
MALAT1
MALT1
MAN1B1
MAOA
MAP2K1
MAP2K3
MAP2K4
MAP2K6
MAP3K11
MAP3K12
MAP3K2
MAP3K5
MAP3K7
MAP3K9
MAP4K4
MAP7
MAPK1
MAPK14
MAPK3
MAPK4
MAPK7
MAPK8
MAPK8IP1
MAPK9
MAPRE1
MARCKS
MAT1A
MAX
MBD2
MBNL1
MBNL2
MBNL3
MCL1
MCM2
MCM3
MDM2
MDM4
MECOM
MECP2
MEF2C
MEF2D
MEG3
MEGF9
MEIS1
MEN1
MEOX2
MEPE
MERTK

MAPK8
MAPK8IP1
MAPK9
MAPRE1
MARCKS
MAX
MBD1
MBD2
MBNL1
MBNL2
MBNL3
MCL1
MCM2
MCM3
MDM2
MDM4
MECOM
MECP2
MEF2C
MEF2D
MEG3
MEGF9
MEN1
MEOX2
MEPE
MERTK
MEST
MET
METTL13
MFF
MFN2
MGMT
MGST2
MIEN1
MITF
MIXL1
MLEC
MLH1
MMP1
MMP12
MMP13
MMP14
MMP15
MMP16
MMP2
MMP24
MMP26
MMP7
MEST
MET
METTL13
MFF
MFN2
MGMT
MGST2
MIEN1
MIF
MITF
MIXL1
MLEC
MLH1
MMP1
MMP12
MMP13
MMP14
MMP15
MMP16
MMP2
MMP24
MMP26
MMP3
MMP9
MNT
MPL
MPRIP
MRC1
MRE11
MRPS27
MSH2
MSH3
MSH6
MSLN
MSMO1
MST1
MT1M
MTA1
MTA2
MTA3
MTAP
MTDH
MTHFR
MTMR14
MTMR3
MTOR
MTTP
MTUS1

MMP9	MUC1
MNT	MUC13
MPL	MXD1
MPRIP	MXI1
MRC1	MYB
MRE11	MYBL1
MRPS27	MYC
MSH2	MYCBP
MSH3	MYCBP2
MSH6	MYCN
MSLN	MYD88
MST1	MYL9
MT1M	MYLIP
MT2A	MYO5A
MTA1	MYO6
MTA2	MYOCD
MTAP	MYRF
MTDH	MYT1
MTHFR	N4BP1
MTMR14	NABP1
MTMR3	NAIP
MTOR	NAMPT
MTTP	NANOG
MTUS1	NASP
MUC1	NAV3
MUC13	NCAM1
MXD1	NCAN
MXI1	NCAPG
MYB	NCOA1
MYBL1	NCOA3
MYC	NCOA6
MYCBP	NCOR2
MYCBP2	NCSTN
MYCN	NDRG2
MYD88	NDST1
MYH1	NDUFA4
MYH2	NECTIN4
MYH4	NEDD4L
MYL9	NEDD9
MYLIP	NES
MYO5A	NET1
MYO6	NEU1
MYOCD	NEUROD1
MYRF	NF1
MYT1	NFAT5
N4BP1	NFATC1
NABP1	NFATC3
NAIP	NFE2L2

NAMPT	NFIA
NANOG	NFIB
NASP	NFIX
NAV3	NFKB1
NCAM1	NFKBIA
NCAN	NFKBIB
NCAPG	NGFR
NCOA1	NIPSNAP1
NCOA3	NIT1
NCOA6	NKD1
NCOR1	NKD2
NCOR2	NKIRAS2
NCSTN	NKRF
NDRG2	NLK
NDST1	NLN
NDUFA4	NLRC5
NECTIN4	NLRP3
NEDD4L	NMI
NEDD9	NOD2
NEK6	NOS1
NES	NOS2
NET1	NOS3
NEU1	NOTCH1
NEUROD1	NOTCH2
NF1	NOTCH3
NF2	NOX4
NFAT5	NPAS3
NFATC1	NPAT
NFATC3	NPEPL1
NFE2L2	NPR1
NFIA	NPTX1
NFIB	NR1H4
NFIX	NR2C2
NFKB1	NR2F2
NFKB2	NR3C1
NFKBIA	NR4A2
NFKBIB	NR5A2
NGFR	NRAS
NID1	NRP1
NIPSNAP1	NRP2
NIT1	NT5E
NKD1	NTF3
NKD2	NTRK2
NKIRAS2	NTRK3
NLK	NUDT1
NLN	NUMB
NLRC5	NUP93
NLRP3	OPRM1

NMI	OSBPL2
NOD2	OSBPL6
NOS2	OSBPL8
NOS3	OTUB1
NOTCH1	OTUD7B
NOTCH2	OXTR
NOX4	P2RX7
NPAS3	P4HB
NPAT	PA2G4
NPR1	PAK1
NPTX1	PAK2
NR1H4	PAK4
NR1I2	PAK5
NR2C2	PAM
NR2F2	PAPPA
NR3C1	PARP1
NR4A2	PARP8
NR5A2	PAX3
NRAS	PAX6
NRP1	PAX7
NRP2	PAX8
NT5E	PBX3
NTF3	PCBP1
NTRK2	PCBP2
NTRK3	PCGF2
NUDT1	PCGF5
NUMB	PCNA
NUP93	PCTP
OPRM1	PDCD4
OSBPL2	PDE4A
OSBPL6	PDGFRA
OSBPL8	PDGFRB
OTUB1	PDLIM7
OTUD7B	PDS5B
OXTR	PEA15
P4HB	PEBP1
PAK1	PER1
PAK2	PGR
PAK4	PHB
PAM	PHF10
PAPPA	PHF8
PARP1	PHLDB2
PARP8	PHLPP1
PATZ1	PHLPP2
PAX3	PIAS3
PAX6	PICSAR
PAX7	PIGF
PBX2	PIK3C2A

PBX3	PIK3CA
PBXIP1	PIK3CB
PCBP1	PIK3CD
PCBP2	PIK3CG
PCGF2	PIK3R1
PCGF5	PIK3R2
PCNA	PIK3R3
PCTP	PIM1
PDCD4	PIN1
PDE4A	PINK1
PDE4B	PITX1
PDGFA	PKD1
PDGFB	PKD2
PDGFC	PKNOX1
PDGFRA	PLAG1
PDGFRB	PLAT
PDIA3	PLAU
PDLIM7	PLAUR
PDS5B	PLCE1
PEA15	PLK1
PEBP1	PLK2
PER1	PLOD2
PGR	PLOD3
PHB	PLXNB1
PHF10	PLXNC1
PHF8	PMEPA1
PHLDB2	PODXL
PHLPP1	POLD1
PHLPP2	POLR3D
PIAS3	POLR3G
PICSAR	POR
PIGF	POU2F2
PIK3C2A	POU4F2
PIK3CB	POU5F1
PIK3CD	PPARA
PIK3CG	PPARG
PIK3R1	PPARGC1A
PIK3R2	PPIF
PIK3R3	PPM1B
PIM1	PPM1D
PIN1	PPM1F
PINK1	PPM1K
PITX1	PPP1CA
PKD1	PPP1CC
PKD2	PPP1R10
PKM	PPP1R13B
PKNOX1	PPP2R2A
PLAG1	PPP2R5E

PLAT	PPP3CA
PLAU	PRAP1
PLAUR	PRDM1
PLCE1	PRDM4
PLK1	PRDX3
PLK2	PRDX6
PLOD2	PRKAA1
PLOD3	PRKAA2
PLXNB1	PRKCD
PLXNC1	PRKCE
PODXL	PRKCH
POLD1	PRKD1
POLR3D	PRKG1
POLR3G	PRKN
POR	PRKRA
POU2F2	PRMT5
POU4F2	PROX1
POU5F1	PRRT2
PPARA	PRRX1
PPARG	PSAP
PPARGC1A	PSMD10
PPIC	PSMD9
PPIF	PTBP2
PPM1D	PTBP3
PPM1F	PTEN
PPM1K	PTENP1
PPP1CA	PTGER2
PPP1CC	PTGES2
PPP1R10	PTGS2
PPP1R13B	PTH1R
PPP2R2A	PTMS
PPP2R5E	PTP4A2
PPP3CA	PTP4A3
PRAP1	PTPN1
PRDM1	PTPN11
PRDM4	PTPN13
PRDX3	PTPN14
PRDX6	PTPN2
PRKAA1	PTPN22
PRKAA2	PTPN9
PRKCD	PTPRF
PRKCE	PTPRJ
PRKCH	PTPRN2
PRKD1	PTPRO
PRKG1	PTTG1
PRKN	PTX3
PRKRA	PUM2
PRMT5	PURA

PROX1
PRRT2
PRRX1
PSAP
PSMD10
PSMD9
PTBP2
PTBP3
PTEN
PTENP1
PTGER2
PTGS2
PTH1R
PTK2
PTMS
PTP4A2
PTPN1
PTPN11
PTPN13
PTPN14
PTPN2
PTPN22
PTPN9
PTPRF
PTPRJ
PTPRN2
PTPRO
PTX3
PURA
PXDN
PXN
QKI
RAB11A
RAB12
RAB14
RAB15
RAB1A
RAB27A
RAB38
RAB40C
RAB5A
RAB5B
RAC1
RAD21
RAD51
RAD52
RAF1
RALA
PVT1
PXDN
PXN
QKI
RAB11A
RAB11FIP1
RAB12
RAB13
RAB14
RAB15
RAB1A
RAB27A
RAB38
RAB5A
RAB5B
RAC1
RAD21
RAD51
RAD52
RAF1
RALA
RAN
RAP1B
RARA
RARB
RASA1
RASAL2
RASGRP1
RASSF1
RASSF6
RASSF8
RAVER2
RB1
RB1CC1
RBL1
RBL2
RBP2
RCAN1
RCBTB1
RCC2
RCOR1
RDX
RECK
REG4
RELA
REST
RET
REV3L

RAN
RAP1B
RARA
RARB
RASA1
RASAL2
RASGRP1
RASSF1
RASSF6
RASSF8
RAVER2
RAX
RB1
RB1CC1
RBL1
RBL2
RBP2
RCAN1
RCBTB1
RCC2
RCOR1
RDX
RECK
REG4
RELN
REST
RET
REV3L
RFFL
RFX6
RFX7
RGMA
RGS16
RGS2
RGS3
RGS5
RHO
RHOA
RHOB
RHOBTB1
RHOC
RICTOR
RMND5A
RNASEL
RND3
RNF144B
RNF2
ROBO1

RFFL
RFX6
RFX7
RGMA
RGS16
RGS2
RGS3
RGS5
RHO
RHOA
RHOB
RHOBTB1
RHOC
RICTOR
RNASEL
RND3
RNF11
RNF144B
RNF2
ROBO1
ROBO2
ROCK1
ROR1
RPA1
RPS6KA1
RPS6KA3
RPS6KA4
RPS6KB1
RPS7
RREB1
RSU1
RTKN
RTN4
RUNX1
RUNX2
RUNX3
RXRA
S100A8
S100B
SAMHD1
SAPCD2
SATB1
SATB2
SAV1
SCARB1
SCD
SCNN1A
SCRIB

ROBO2
ROCK1
ROCK2
ROR1
RPA1
RPS6KA1
RPS6KA3
RPS6KA5
RPS6KB1
RPS7
RREB1
RRM2
RSU1
RTKN
RTN4
RUNX1
RUNX2
RUNX3
RXRA
S100A1
S100A8
S100B
S1PR1
SAMHD1
SAPCD2
SATB1
SATB2
SAV1
SCARB1
SCD
SCNN1A
SCRIB
SDC1
SDHA
SDHD
SELE
SEMA3A
SEMA4C
SEMA4D
SEMA6A
SEMA7A
SENP1
SEPT7
SERBP1
SERINC5
SERPINB5
SERPINB9
SERPINE1

SDHA
SDHD
SELE
SEMA3A
SEMA4C
SEMA4D
SEMA6A
SEMA7A
SENP1
SEPT7
SERBP1
SERINC5
SERPINB5
SERPINB9
SERPINE1
SERPINF2
SERPINH1
SERPINI1
SET
SETD2
SETDB1
SFRP1
SFRP2
SFRP5
SGPL1
SGPP2
SH3BGRL
SH3GL1
SH3PXD2A
SHC1
SHMT2
SIAH2
SIGLEC1
SIKE1
SIRPA
SIRT1
SIRT2
SIRT6
SIRT7
SIVA1
SIX1
SKI
SKP2
SLAIN1
SLC16A1
SLC16A2
SLC1A2
SLC22A7

SERPINF2
SERPINH1
SERPINI1
SET
SETD2
SETDB1
SFPQ
SFRP1
SFRP2
SFRP5
SGPL1
SGPP2
SH3BGRL
SH3GL1
SH3PXD2A
SHC1
SHMT2
SIAH2
SIGLEC1
SIRPA
SIRT1
SIRT2
SIRT6
SIRT7
SIX1
SKI
SKP2
SLAIN1
SLC16A1
SLC16A2
SLC1A2
SLC22A7
SLC2A1
SLC2A3
SLC2A4
SLC45A3
SLC6A4
SLC6A8
SLC7A11
SLC7A6
SMAD1
SMAD2
SMAD3
SMAD4
SMAD5
SMAD7
SMARCA2
SMARCA4
SLC2A1
SLC2A3
SLC2A4
SLC5A5
SLC6A4
SLC6A8
SLC7A11
SLC7A6
SLPI
SMAD1
SMAD2
SMAD3
SMAD4
SMAD5
SMAD7
SMARCA2
SMARCA4
SMARCA5
SMN1
SMO
SMR3B
SMURF1
SNAI1
SNAI2
SNAI3
SNIP1
SOCS1
SOCS3
SOCS5
SOCS6
SOCS7
SOD2
SOD3
SOS1
SOST
SOX17
SOX2
SOX4
SOX5
SOX6
SOX9
SP1
SP3
SP4
SP7
SPARC
SPHK1
SPI1

SMARCA5
SMN1
SMO
SMURF1
SNAI1
SNAI2
SNAI3
SND1
SOCS1
SOCS3
SOCS5
SOCS6
SOCS7
SOD2
SOD3
SOST
SOX17
SOX2
SOX4
SOX5
SOX6
SOX9
SP1
SP3
SP4
SP7
SPARC
SPHK1
SPI1
SPRED1
SPRED2
SPRY1
SPRY2
SPRY3
SPRY4
SPTBN1
SPTLC1
SRC
SRGAP1
SRGAP2
SRR
SRSF1
SRSF10
SRSF11
SSSCA1
SSX2IP
ST14
ST3GAL6
SPRED1
SPRED2
SPRY1
SPRY2
SPRY3
SPRY4
SPTBN1
SPTLC1
SRC
SREBF1
SREBF2
SRF
SRGAP1
SRGAP2
SRR
SRSF1
SRSF10
SRSF11
SSSCA1
SSX2IP
ST14
ST3GAL6
ST7L
ST8SIA4
STAG2
STAM2
STARD13
STAT1
STAT3
STAT5A
STAT5B
STK11
STK40
STMN1
STRADB
STUB1
STX16
STX1A
SUFU
SUV39H1
SUZ12
SWAP70
SYK
SYT1
TAB1
TAB2
TAB3
TAC1

ST7L
ST8SIA4
STAG2
STAM2
STARD13
STAT1
STAT3
STAT5A
STAT5B
STAT6
STK11
STK40
STMN1
STRADB
STUB1
STX16
STX1A
SUFU
SUV39H1
SUZ12
SWAP70
SYK
SYT1
TAB1
TAB3
TAC1
TACC1
TACC3
TAL1
TAP1
TARBP1
TBC1D1
TBC1D2
TBCCD1
TBK1
TBX21
TBXA2R
TCEAL1
TCEAL9
TCF21
TCF3
TCF4
TCF7
TCL1A
TDG
TERF2
TERT
TET1

TACC1
TACC3
TAL1
TAP1
TARBP1
TBC1D1
TBC1D2
TBC1D9
TBK1
TBXA2R
TCEAL1
TCEAL9
TCF21
TCF3
TCF4
TCF7
TDG
TERT
TET1
TET2
TET3
TEX261
TF
TFAM
TFAP2A
TFAP2C
TFEB
TFF1
TFF2
TFPI
TFRC
TGFA
TGFB1
TGFB2
TGFBI
TGFBR1
TGFBR2
TGFBR3
TGFBRAP1
TGIF1
TGIF2
THAP2
THBS1
THBS2
THRB
THSD7A
TIAM1
TICAM1

TET2
TET3
TEX261
TFAM
TFAP2A
TFAP2C
TFEB
TFF1
TFF2
TFPI
TFRC
TGFA
TGFB1
TGFB2
TGFB3
TGFBI
TGFBR1
TGFBR2
TGFBR3
TGFBRAP1
TGIF1
TGIF2
THAP2
THBS1
THBS2
THRB
THSD7A
TIAM1
TICAM1
TICAM2
TIMP1
TIMP2
TIMP3
TIRAP
TJAP1
TLN2
TLR2
TLR3
TLR4
TLR7
TM9SF3
TMC7
TMED7
TMEM2
TMEM64
TMEM92
TMEM9B
TMOD3
TICAM2
TIMP1
TIMP2
TIMP3
TIRAP
TJAP1
TLE3
TLN2
TLR2
TLR3
TLR4
TLR7
TM9SF3
TMC7
TMED7
TMEM2
TMEM92
TMEM9B
TMOD3
TNC
TNF
TNFAIP3
TNFRSF10A
TNFRSF10B
TNFRSF12A
TNFSF10
TNFSF11
TNFSF12
TNFSF13
TNK2
TNRC6A
TOB1
TOPORS
TOX
TP53
TP53BP2
TP53COR1
TP53I11
TP53INP1
TP63
TP73
TPD52
TPM1
TPM3
TPPP3
TPRG1
TRA2B
TRAF3IP2

TNC	TRAF4
TNF	TRAF5
TNFAIP3	TRAF6
TNFRSF10A	TRAF7
TNFRSF10B	TRAPPC2B
TNFSF10	TREM2
TNFSF11	TRIB1
TNFSF12	TRIB2
TNFSF13	TRIB3
TNK2	TRIM11
TNRC6A	TRIM27
TOB1	TRIM29
TOP1	TRIM68
TOPORS	TRIM8
TOX	TRPC5
TP53	TRPC6
TP53BP2	TRPS1
TP53COR1	TSG101
TP53I11	TSPAN6
TP53INP1	TTK
TP63	TUBB4B
TP73	TUG1
TPD52	TUSC2
TPM1	TUSC3
TPM3	TWF1
TPPP3	TWIST1
TPRG1	UBE2C
TRA2B	UBE2F
TRAF3IP2	UBE2I
TRAF4	UBE2N
TRAF5	UBE3C
TRAF7	UBR5
TRAPPC2B	UCA1
TREM2	UCP1
TRIB1	UGT2B15
TRIB2	UGT2B17
TRIB3	UHRF1
TRIM11	ULBP2
TRIM27	ULK1
TRIM29	ULK2
TRIM68	UNG
TRIM71	USF2
TRIM8	USP14
TRPC5	USP18
TRPC6	USP9X
TRPS1	UVRAG
TSC1	VAMP2
TSG101	VDAC1

TSPAN6
TTK
TUBB4B
TUG1
TUSC3
TWF1
TWIST1
TWIST2
UBE2C
UBE2F
UBE2I
UBE2N
UBE3C
UBR5
UCA1
UCP1
UGT2B15
UGT2B17
UHRF1
UHRF2
ULBP2
ULK1
ULK2
UNG
USF2
USP14
USP18
USP4
USP9X
UVRAG
VAMP2
VAV2
VDAC1
VDR
VEGFA
VEGFC
VEZT
VGLL4
VHL
VIM
VLDLR
VMP1
VOPP1
VPS4B
VPS51
WASF3
WDR4
WDR77

VDR
VEGFA
VEGFC
VEZT
VGLL4
VHL
VIM
VLDLR
VMP1
VOPP1
VPS4B
VPS51
WASF2
WASF3
WDR4
WDR77
WEE1
WIF1
WNK1
WNK4
WNT1
WNT3A
WNT4
WT1
WWP1
XBP1
XIAP
XIST
XPA
XRCC3
XRCC5
YAP1
YBX1
YBX3
YES1
YOD1
YWHAZ
YY1
YY1AP1
ZAP70
ZBTB10
ZBTB2
ZBTB4
ZBTB7A
ZCCHC11
ZDHHC9
ZEB1
ZEB2

WEE1
WIF1
WNK1
WNK4
WNT1
WNT10B
WNT3A
WNT4
WNT7A
WT1
WWP1
XBP1
XIAP
XIST
XPA
XRCC3
XRCC5
YAP1
YBX1
YBX3
YES1
YOD1
YWHAZ
YY1
YY1AP1
ZAP70
ZBTB10
ZBTB2
ZBTB4
ZBTB7A
ZCCHC11
ZEB1
ZEB2
ZFP36
ZFYVE9
ZHX1
ZNF215
ZNF217
ZNF763
ZNFX1
ZNRF2
ZWINT
ZYX

ZFP36
ZFYVE9
ZHX1
ZNF215
ZNF217
ZNF763
ZNFX1
ZNRF2
ZNRF3
ZWINT
ZYX

Shared first quartile hASC-EVs and hAMSC-EVs miRNAs targets

ABCA1
ABCB1
ABCB9
ABCC1
ABCG2
ABHD17C
ABL1
ABL2
ABRACL
ABTB1
ACKR3
ACLY
ACSL1
ACSL4
ACTB
ACTG1
ACTN1
ACVR1
ACVR1B
ACVR1C
ACVR2A
ADAM12
ADAM17
ADAM1A
ADAMTS6
ADAMTS9
ADAR
ADD3
ADORA2A
ADORA2B
AGO2
AGPAT2
AGTR1
AHR
AHRR
AIFM3
AIP
AKR1B10
AKR1C2
AKT1
AKT2
AKT3
ALCAM
ALDH3A1

ALDH5A1
ALOX5
ALPK2
ALPPL2
AMACR
ANG
ANGPT2
ANGPTL4
ANK3
ANKH
ANKRD46
ANP32A
ANXA1
AP1G1
AP2M1
APAF1
APC
APH1A
API5
APLN
APOE
APP
AQP1
AQP4
AR
ARF1
ARF4
ARF6
ARHGAP12
ARHGAP19
ARHGAP32
ARHGDIA
ARHGDIB
ARID1A
ARID3A
ARID3B
ARID4B
ARIH2
ARL2
ARL4C
ARL6IP5
ARNT
ARPC5
ARPC5L
ARPP19
ARTN
ASF1B
ASZ1

ATF1
ATF4
ATG12
ATG14
ATG16L1
ATG2B
ATG4A
ATG4B
ATG4C
ATG4D
ATG5
ATG7
ATG9A
ATM
ATP2A2
ATP5S
ATXN1
AURKB
AVEN
AXIN2
AXL
BACE1
BAG1
BAG4
BAG5
BAK1
BAMBI
BANP
BAP1
BASP1
BAX
BBC3
BCAR1
BCL10
BCL11A
BCL2
BCL2L11
BCL2L2
BCL3
BCL6
BCL7A
BCL9
BDNF
BECN1
BIRC5
BIRC6
BMF
BMI1

BMP2
BMP6
BMP7
BMPR1B
BMPR2
BNIP2
BNIP3
BNIP3L
BRAF
BRAP
BRCA1
BRCA2
BSG
BTG1
BTG2
BTK
BTRC
C11orf65
C1QTNF9
CACNA1C
CACNB3
CADM1
CALCR
CAMK1D
CAMK2D
CAPNS1
CAPRIN1
CARD10
CARM1
CASC2
CASP3
CASP7
CASP8
CASP8AP2
CASR
CAT
CAV2
CBFB
CBX3
CBX4
CCDC43
CCDC6
CCKBR
CCL1
CCL20
CCL22
CCL26
CCL3

CCL5
CCL8
CCNA2
CCNB1
CCND1
CCND2
CCND3
CCNE1
CCNE2
CCNG1
CCNJ
CCNT1
CCNT2
CCR1
CD151
CD24
CD274
CD276
CD28
CD4
CD40
CD44
CD69
CD93
CD99
CDC25A
CDC27
CDC42
CDC6
CDC7
CDH1
CDH11
CDH2
CDH5
CDK1
CDK2
CDK2AP1
CDK4
CDK6
CDK7
CDK8
CDK9
CDKN1A
CDKN1B
CDKN1C
CDKN2A
CDKN2C
CDKN2D

CDS2
CDX1
CDX2
CEACAM6
CEBPA
CEBPB
CEP19
CERS2
CFTR
CGN
CHD1
CHEK1
CHL1
CHORDC1
CHUK
CKS2
CLDN1
CLDN2
CLINT1
CLOCK
CLTC
CLU
CMPK1
CNOT6
COL10A1
COL15A1
COL16A1
COL1A1
COL1A2
COL21A1
COL3A1
COL4A1
COL4A2
COL5A1
COL5A2
COL7A1
COPS5
COPS8
CORO1A
COX2
CPD
CPEB1
CPEB2
CPEB3
CPEB4
CREB1
CREB5
CREBZF

CREG1
CRIM1
CRISP2
CRK
CRKL
CRNDE
Crtc1
CSF1
CSF1R
CSNK2A1
CTCF
CTDSP2
CTDSPL
CTGF
CTHRC1
CTNNB1
CTNND1
CUL2
CUL5
CX3CL1
CXCL12
CXCL2
CXCL8
CXCR2
CXCR4
CYB5A
CYBB
CYLD
CYP11B2
CYP19A1
CYP1B1
CYP24A1
CYP2B6
CYP2C19
CYP2C9
CYP3A4
CYP7B1
CYR61
DAAM2
DAB2
DACT3
DAPK3
DAXX
DDAH1
DDC
DDIT4
DDX17
DDX3X

DDX6
DEDD
DERL1
DFFA
DGAT1
DGUOK
DHFR
DHFRP1
DICER1
DIMT1
DIO1
DIRAS3
DKK1
DKK2
DKK3
DLL1
DLL4
DMD
DNAJA4
DNAJC27
DND1
DNM1L
DNMT1
DNMT3A
DNMT3B
DOCK1
DOCK4
DOCK5
DOCK7
DOHH
DPYD
DPYSL2
DRAM2
DRD1
DSC2
DTD1
DTL
DUSP10
DUSP2
DUSP4
DUSP5
DUSP6
DVL2
DYRK2
E2F1
E2F2
E2F3
E2F5

E2F6
EBP
ECT2
EDNRA
EEF1A2
EFNA1
EFNA3
EGF
EGFR
EGLN1
EGLN3
EGR1
EGR2
EHD2
EIF2S1
EIF2S3
EIF4A2
EIF4E
EIF4EBP1
EIF5A2
ELAVL1
ELF2
ELN
EMSY
ENPEP
EOMES
EP300
EPAS1
EPB41L3
EPHA2
EPHA4
EPHA5
EPN2
EPO
EPOR
ERBB2
ERBB3
ERCC1
ERG
ESR1
ESR2
ESRRG
ETS1
ETV1
EYA2
EYA4
EZH2
F11R

FAF1
FAM129A
FAM3C
FAM45A
FAS
FASLG
FASN
FASTK
FBN1
FBXO11
FBXO31
FBXO8
FBXW7
FEN1
FES
FGA
FGB
FGF11
FGF2
FGF21
FGF9
FGFR1
FGFR2
FGFR3
FGFRL1
FGG
FH
FHIT
FIS1
FKBP1B
FKBP5
FLI1
FLOT2
FLT1
FMN2
FMNL3
FMOD
FMR1
FOS
FOSB
FOSL1
FOXA1
FOXA2
FOXC1
FOXD1
FOXJ3
FOXL2
FOXM1

FOXN3
FOXO1
FOXO3
FOXP1
FOXP3
FRAT1
FRAT2
FSCN1
FSTL1
FURIN
FUT4
FUT8
FXN
FYN
FZD3
FZD4
FZD5
FZD6
FZD7
GAB1
GAB2
GALNT7
GAPDH
GAS1
GAS5
GATA2
GATA3
GATA4
GATA6
GCM1
GDAP1
GDF5
GFRA3
GIT1
GJA1
GLI1
GLI2
GLS2
GLUL
GMFB
GNA13
GNAI1
GNAI2
GNAI3
GOLM1
GP1BA
GPC1
GPD1L

GPR137B
GPR78
GPR85
GRB10
GRB2
GRIN2A
GRM4
GRM7
GSK3B
GSR
GSS
H2AFX
HAND2
HAS2
HAX1
HBEGF
HBP1
HCC
HDAC1
HDAC11
HDAC2
HDAC4
HDAC6
HDGF
HECTD2
HGF
HGS
HIF1A
HIF1AN
HIF3A
HIP1R
HIPK1
HIPK2
HIPK3
HK2
HLA-G
HLTF
HMGA1
HMGA2
HMGB1
HMGB3
HMGCR
HMGXB4
HMOX1
HNF4A
HNF4G
HNRNPK
HOTAIR

HOTTIP
HOXA1
HOXA10
HOXA5
HOXA9
HOXB3
HOXB5
HOXC13
HOXD10
HPGD
HRAS
HS3ST2
HSD17B1
HSP90B1
HSPA4
HSPA5
HSPB2
HSPB6
HTR2C
ICAM1
ICAM2
ICOSLG
ID4
IDH1
IER2
IFITM1
IFNAR1
IFNB1
IFNG
IFNR
IGF1
IGF1R
IGF2
IGF2BP1
IGF2BP3
IGFBP1
IGFBP3
IKBKB
IKBKE
IKZF1
IKZF2
IKZF3
IKZF4
IL10
IL11
IL12A
IL12B
IL18

IL1A
IL1B
IL21R
IL25
IL34
IL4
IL6
IL6R
ILK
IMPA1
IMPDH1
IMPDH2
ING4
ING5
INHBB
INPP4B
INPP5A
INSIG1
IRAK1
IRAK4
IRF1
IRF2
IRF4
IRF5
IRS1
IRS2
ISCU
ITCH
ITGA11
ITGA3
ITGA5
ITGA6
ITGB1
ITGB3
ITGB8
ITIH5
JADE1
JAG1
JAK1
JAK2
JAZF1
JMY
JPH2
JPT1
KAT2B
KCMF1
KCNH1
KCNH2

KDM4A
KDM5B
KDR
KEAP1
KHSRP
KIF26B
KIT
KITLG
KLB
KLC2
KLF12
KLF13
KLF15
KLF2
KLF4
KLF5
KLF6
KLF9
KLHL11
KLK10
KMT5A
KPNA2
KRAS
KREMEN1
KREMEN2
L1CAM
LACTB
LAMB3
LAMC1
LAMC2
LAMP2
LARP1
LASP1
LATS2
LDHA
LDHB
LDLR
LEF1
LGALS1
LGALS9
LGR4
LIF
LIFR
LIMK1
LIN28A
LIN28B
LIPA
LMO2

LOX
LOXL2
LPL
LRG1
LRP6
LRRC8A
LRRFIP1
LTF
LYPLA2
LZTS1
MAD2L1
MAFB
MAGEA12
MAGEA2
MAGEA3
MAGEA6
MALAT1
MALT1
MAN1B1
MAOA
MAP2K1
MAP2K3
MAP2K4
MAP2K6
MAP3K11
MAP3K12
MAP3K2
MAP3K5
MAP3K7
MAP3K9
MAP4K4
MAP7
MAPK1
MAPK14
MAPK4
MAPK7
MAPK8
MAPK8IP1
MAPK9
MAPRE1
MARCKS
MAX
MBD2
MBNL1
MBNL2
MBNL3
MCL1
MCM2

MCM3
MDM2
MDM4
MECOM
MECP2
MEF2C
MEF2D
MEG3
MEGF9
MEN1
MEOX2
MEPE
MERTK
MEST
MET
METTL13
MFF
MFN2
MGMT
MGST2
MIEN1
MITF
MIXL1
MLEC
MLH1
MMP1
MMP12
MMP13
MMP14
MMP15
MMP16
MMP2
MMP24
MMP26
MMP9
MNT
MPL
MPRIP
MRC1
MRE11
MRPS27
MSH2
MSH3
MSH6
MSLN
MST1
MT1M
MTA1

MTA2
MTAP
MTDH
MTHFR
MTMR14
MTMR3
MTOR
MTTP
MTUS1
MUC1
MUC13
MXD1
MXI1
MYB
MYBL1
MYC
MYCBP
MYCBP2
MYCN
MYD88
MYL9
MYLIP
MYO5A
MYO6
MYOCD
MYRF
MYT1
N4BP1
NABP1
NAIP
NAMPT
NANOG
NASP
NAV3
NCAM1
NCAN
NCAPG
NCOA1
NCOA3
NCOA6
NCOR2
NCSTN
NDRG2
NDST1
NDUFA4
NECTIN4
NEDD4L
NEDD9

NES
NET1
NEU1
NEUROD1
NF1
NFAT5
NFATC1
NFATC3
NFE2L2
NFIA
NFIB
NFIX
NFKB1
NFKBIA
NFKBIB
NGFR
NIPSNAP1
NIT1
NKD1
NKD2
NKIRAS2
NLK
NLN
NLRC5
NLRP3
NMI
NOD2
NOS2
NOS3
NOTCH1
NOTCH2
NOX4
NPAS3
NPAT
NPR1
NPTX1
NR1H4
NR2C2
NR2F2
NR3C1
NR4A2
NR5A2
NRAS
NRP1
NRP2
NT5E
NTF3
NTRK2

NTRK3
NUDT1
NUMB
NUP93
OPRM1
OSBPL2
OSBPL6
OSBPL8
OTUB1
OTUD7B
OXTR
P4HB
PAK1
PAK2
PAK4
PAM
PAPPA
PARP1
PARP8
PAX3
PAX6
PAX7
PBX3
PCBP1
PCBP2
PCGF2
PCGF5
PCNA
PCTP
PDCD4
PDE4A
PDGFRA
PDGFRB
PDLIM7
PDS5B
PEA15
PEBP1
PER1
PGR
PHB
PHF10
PHF8
PHLDB2
PHLPP1
PHLPP2
PIAS3
PICSAR
PIGF

PIK3C2A
PIK3CB
PIK3CD
PIK3CG
PIK3R1
PIK3R2
PIK3R3
PIM1
PIN1
PINK1
PITX1
PKD1
PKD2
PKNOX1
PLAG1
PLAT
PLAU
PLAUR
PLCE1
PLK1
PLK2
PLOD2
PLOD3
PLXNB1
PLXNC1
PODXL
POLD1
POLR3D
POLR3G
POR
POU2F2
POU4F2
POU5F1
PPARA
PPARG
PPARGC1A
PPIF
PPM1D
PPM1F
PPM1K
PPP1CA
PPP1CC
PPP1R10
PPP1R13B
PPP2R2A
PPP2R5E
PPP3CA
PRAP1

PRDM1
PRDM4
PRDX3
PRDX6
PRKAA1
PRKAA2
PRKCD
PRKCE
PRKCH
PRKD1
PRKG1
PRKN
PRKRA
PRMT5
PROX1
PRRT2
PRRX1
PSAP
PSMD10
PSMD9
PTBP2
PTBP3
PTEN
PTENP1
PTGER2
PTGS2
PTH1R
PTMS
PTP4A2
PTPN1
PTPN11
PTPN13
PTPN14
PTPN2
PTPN22
PTPN9
PTPRF
PTPRJ
PTPRN2
PTPRO
PTX3
PURA
PXDN
PXN
QKI
RAB11A
RAB12
RAB14

RAB15
RAB1A
RAB27A
RAB38
RAB5A
RAB5B
RAC1
RAD21
RAD51
RAD52
RAF1
RALA
RAN
RAP1B
RARA
RARB
RASA1
RASAL2
RASGRP1
RASSF1
RASSF6
RASSF8
RAVER2
RB1
RB1CC1
RBL1
RBL2
RBP2
RCAN1
RCBTB1
RCC2
RCOR1
RDX
RECK
REG4
REST
RET
REV3L
RFFL
RFX6
RFX7
RGMA
RGS16
RGS2
RGS3
RGS5
RHO
RHOA

RHOB
RHOBTB1
RHOC
RICTOR
RNASEL
RND3
RNF144B
RNF2
ROBO1
ROBO2
ROCK1
ROR1
RPA1
RPS6KA1
RPS6KA3
RPS6KB1
RPS7
RREB1
RSU1
RTKN
RTN4
RUNX1
RUNX2
RUNX3
RXRA
S100A8
S100B
SAMHD1
SAPCD2
SATB1
SATB2
SAV1
SCARB1
SCD
SCNN1A
SCRIB
SDHA
SDHD
SELE
SEMA3A
SEMA4C
SEMA4D
SEMA6A
SEMA7A
SENP1
SEPT7
SERBP1
SERINC5

SERPINB5
SERPINB9
SERPINE1
SERPINF2
SERPINH1
SERPINI1
SET
SETD2
SETDB1
SFRP1
SFRP2
SFRP5
SGPL1
SGPP2
SH3BGRL
SH3GL1
SH3PXD2A
SHC1
SHMT2
SIAH2
SIGLEC1
SIRPA
SIRT1
SIRT2
SIRT6
SIRT7
SIX1
SKI
SKP2
SLAIN1
SLC16A1
SLC16A2
SLC1A2
SLC22A7
SLC2A1
SLC2A3
SLC2A4
SLC6A4
SLC6A8
SLC7A11
SLC7A6
SMAD1
SMAD2
SMAD3
SMAD4
SMAD5
SMAD7
SMARCA2

SMARCA4
SMARCA5
SMN1
SMO
SMURF1
SNAI1
SNAI2
SNAI3
SOCS1
SOCS3
SOCS5
SOCS6
SOCS7
SOD2
SOD3
SOST
SOX17
SOX2
SOX4
SOX5
SOX6
SOX9
SP1
SP3
SP4
SP7
SPARC
SPHK1
SPI1
SPRED1
SPRED2
SPRY1
SPRY2
SPRY3
SPRY4
SPTBN1
SPTLC1
SRC
SRGAP1
SRGAP2
SRR
SRSF1
SRSF10
SRSF11
SSSCA1
SSX2IP
ST14
ST3GAL6

ST7L
ST8SIA4
STAG2
STAM2
STARD13
STAT1
STAT3
STAT5A
STAT5B
STK11
STK40
STMN1
STRADB
STUB1
STX16
STX1A
SUFU
SUV39H1
SUZ12
SWAP70
SYK
SYT1
TAB1
TAB3
TAC1
TACC1
TACC3
TAL1
TAP1
TARBP1
TBC1D1
TBC1D2
TBK1
TBXA2R
TCEAL1
TCEAL9
TCF21
TCF3
TCF4
TCF7
TDG
TERT
TET1
TET2
TET3
TEX261
TFAM
TFAP2A

TFAP2C
TFEB
TFF1
TFF2
TFPI
TFRC
TGFA
TGFB1
TGFB2
TGFBI
TGFBR1
TGFBR2
TGFBR3
TGFBRAP1
TGIF1
TGIF2
THAP2
THBS1
THBS2
THRB
THSD7A
TIAM1
TICAM1
TICAM2
TIMP1
TIMP2
TIMP3
TIRAP
TJAP1
TLN2
TLR2
TLR3
TLR4
TLR7
TM9SF3
TMC7
TMED7
TMEM2
TMEM92
TMEM9B
TMOD3
TNC
TNF
TNFAIP3
TNFRSF10A
TNFRSF10B
TNFSF10
TNFSF11

TNFSF12
TNFSF13
TNK2
TNRC6A
TOB1
TOPORS
TOX
TP53
TP53BP2
TP53COR1
TP53I11
TP53INP1
TP63
TP73
TPD52
TPM1
TPM3
TPPP3
TPRG1
TRA2B
TRAF3IP2
TRAF4
TRAF5
TRAF7
TRAPPC2B
TREM2
TRIB1
TRIB2
TRIB3
TRIM11
TRIM27
TRIM29
TRIM68
TRIM8
TRPC5
TRPC6
TRPS1
TSG101
TSPAN6
TTK
TUBB4B
TUG1
TUSC3
TWF1
TWIST1
UBE2C
UBE2F
UBE2I

UBE2N
UBE3C
UBR5
UCA1
UCP1
UGT2B15
UGT2B17
UHRF1
ULBP2
ULK1
ULK2
UNG
USF2
USP14
USP18
USP9X
UVRAG
VAMP2
VDAC1
VDR
VEGFA
VEGFC
VEZT
VGLL4
VHL
VIM
VLDLR
VMP1
VOPP1
VPS4B
VPS51
WASF3
WDR4
WDR77
WEE1
WIF1
WNK1
WNK4
WNT1
WNT3A
WNT4
WT1
WWP1
XBP1
XIAP
XIST
XPA
XRCC3

XRCC5
YAP1
YBX1
YBX3
YES1
YOD1
YWHAZ
YY1
YY1AP1
ZAP70
ZBTB10
ZBTB2
ZBTB4
ZBTB7A
ZCCHC11
ZEB1
ZEB2
ZFP36
ZFYVE9
ZHX1
ZNF215
ZNF217
ZNF763
ZNFX1
ZNRF2
ZWINT
ZYX

Targets exclusive of first quartile hASC-EVs miRNAs

ADGRA2
AGO1
AGO4
ARG2
ARHGEF3
ATAT1
BAG3
BCL2L1
BLCAP
BMP1
CAB39
CASP9
CCL4
CCR7
CDC25B
CDC34
COL5A3
COPS6
CTNNBIP1
CXCR6
CYTOR
DNAJB11
DTNB
EED
EID1
ERRFI1
EWSR1
FAM83F
FANCG
FERMT2
FNDC3B
G6PC
GABARAPL1
GLS
GPS1
GRN
HES1
HMGB2
HMGN2
HNF1B
HOXB4
HSP90AA1
IL13RA1
IL32

INO80
INSR
ITGB4
KDM2A
KDM5C
KIAA0100
KIF1B
KLF3
LAMP1
LCN2
LOXL4
LPAR1
LRP5
MACC1
MAP3K4
MAP4K3
MBD1
MMP7
MT2A
MYH1
MYH2
MYH4
NCOR1
NEK6
NF2
NFKB2
NID1
NR1I2
PATZ1
PBX2
PBXIP1
PDE4B
PDGFA
PDGFB
PDGFC
PDIA3
PKM
PPIC
PTK2
RAB40C
RAX
RELN
RMND5A
ROCK2
RPS6KA5
RRM2
S100A1
S1PR1

SDC1
SFPQ
SLC45A3
SND1
STAT6
TBCCD1
TBX21
TCL1A
TERF2
TGFB3
TMEM64
TOP1
TRIM71
TSC1
TWIST2
UHRF2
USP4
VAV2
WNT10B
WNT7A

Targets exclusive of first quartile hAMSC-EVs miRNAs

ADRB1
AKAP12
ARHGAP5
ATP7A
BCLAF1
CCL2
CD22
CD40LG
CD80
CDC14A
CDKN3
CFH
CKB
CNOT6L
CPM
CTBP2
DUSP1
EPSTI1
ERBB4
FADD
FAM160B2
FANCM
GEMIN4
GOLPH3
HNRNPD
IKBKG
IRAK2
IS2
KIF22
LFNG
LRP2
MAGI2
MAPK3
MAT1A
MEIS1
MIF
MMP3
MSMO1
MTA3
NKRF
NOS1
NOTCH3
NPEPL1
P2RX7

PA2G4
PAK5
PAX8
PIK3CA
PMEPA1
PPM1B
PTGES2
PTP4A3
PTTG1
PUM2
PVT1
RAB11FIP1
RAB13
RELA
RNF11
RPS6KA4
SIKE1
SIVA1
SLC5A5
SLPI
SMR3B
SNIP1
SOS1
SREBF1
SREBF2
SRF
TAB2
TBC1D9
TF
TLE3
TNFRSF12A
TRAF6
TUSC2
WASF2
ZDHHC9
ZNRF3

Total targets of hASC-EVs and hAMSC-EVs first quartiles miRNAs

ABCA1
ABCB1
ABCB9
ABCC1
ABCG2
ABHD17C
ABL1
ABL2
ABRACL
ABTB1
ACKR3
ACLY
ACSL1
ACSL4
ACTB
ACTG1
ACTN1
ACVR1
ACVR1B
ACVR1C
ACVR2A
ADAM12
ADAM17
ADAM1A
ADAMTS6
ADAMTS9
ADAR
ADD3
ADGRA2
ADORA2A
ADORA2B
ADRB1
AGO1
AGO2
AGO4
AGPAT2
AGTR1
AHR
AHRR
AIFM3
AIP
AKAP12
AKR1B10
AKR1C2

AKT1
AKT2
AKT3
ALCAM
ALDH3A1
ALDH5A1
ALOX5
ALPK2
ALPPL2
AMACR
ANG
ANGPT2
ANGPTL4
ANK3
ANKH
ANKRD46
ANP32A
ANXA1
AP1G1
AP2M1
APAF1
APC
APH1A
API5
APLN
APOE
APP
AQP1
AQP4
AR
ARF1
ARF4
ARF6
ARG2
ARHGAP12
ARHGAP19
ARHGAP32
ARHGAP5
ARHGDIA
ARHGDIB
ARHGEF3
ARID1A
ARID3A
ARID3B
ARID4B
ARIH2
ARL2
ARL4C

ARL6IP5
ARNT
ARPC5
ARPC5L
ARPP19
ARTN
ASF1B
ASZ1
ATAT1
ATF1
ATF4
ATG12
ATG14
ATG16L1
ATG2B
ATG4A
ATG4B
ATG4C
ATG4D
ATG5
ATG7
ATG9A
ATM
ATP2A2
ATP5S
ATP7A
ATXN1
AURKB
AVEN
AXIN2
AXL
BACE1
BAG1
BAG3
BAG4
BAG5
BAK1
BAMBI
BANP
BAP1
BASP1
BAX
BBC3
BCAR1
BCL10
BCL11A
BCL2
BCL2L1

BCL2L11
BCL2L2
BCL3
BCL6
BCL7A
BCL9
BCLAF1
BDNF
BECN1
BIRC5
BIRC6
BLCAP
BMF
BMI1
BMP1
BMP2
BMP6
BMP7
BMPR1B
BMPR2
BNIP2
BNIP3
BNIP3L
BRAF
BRAP
BRCA1
BRCA2
BSG
BTG1
BTG2
BTK
BTRC
C11orf65
C1QTNF9
CAB39
CACNA1C
CACNB3
CADM1
CALCR
CAMK1D
CAMK2D
CAPNS1
CAPRIN1
CARD10
CARM1
CASC2
CASP3
CASP7

CASP8
CASP8AP2
CASP9
CASR
CAT
CAV2
CBFB
CBX3
CBX4
CCDC43
CCDC6
CCKBR
CCL1
CCL2
CCL20
CCL22
CCL26
CCL3
CCL4
CCL5
CCL8
CCNA2
CCNB1
CCND1
CCND2
CCND3
CCNE1
CCNE2
CCNG1
CCNJ
CCNT1
CCNT2
CCR1
CCR7
CD151
CD22
CD24
CD274
CD276
CD28
CD4
CD40
CD40LG
CD44
CD69
CD80
CD93
CD99

CDC14A
CDC25A
CDC25B
CDC27
CDC34
CDC42
CDC6
CDC7
CDH1
CDH11
CDH2
CDH5
CDK1
CDK2
CDK2AP1
CDK4
CDK6
CDK7
CDK8
CDK9
CDKN1A
CDKN1B
CDKN1C
CDKN2A
CDKN2C
CDKN2D
CDKN3
CDS2
CDX1
CDX2
CEACAM6
CEBPA
CEBPB
CEP19
CERS2
CFH
CFTR
CGN
CHD1
CHEK1
CHL1
CHORDC1
CHUK
CKB
CKS2
CLDN1
CLDN2
CLINT1

CLOCK
CLTC
CLU
CMPK1
CNOT6
CNOT6L
COL10A1
COL15A1
COL16A1
COL1A1
COL1A2
COL21A1
COL3A1
COL4A1
COL4A2
COL5A1
COL5A2
COL5A3
COL7A1
COPS5
COPS6
COPS8
CORO1A
COX2
CPD
CPEB1
CPEB2
CPEB3
CPEB4
CPM
CREB1
CREB5
CREBZF
CREG1
CRIM1
CRISP2
CRK
CRKL
CRNDE
Crtc1
CSF1
CSF1R
CSNK2A1
CTBP2
CTCF
CTDSP2
CTDSPL
CTGF

CTHRC1
CTNNB1
CTNNBIP1
CTNND1
CUL2
CUL5
CX3CL1
CXCL12
CXCL2
CXCL8
CXCR2
CXCR4
CXCR6
CYB5A
CYBB
CYLD
CYP11B2
CYP19A1
CYP1B1
CYP24A1
CYP2B6
CYP2C19
CYP2C9
CYP3A4
CYP7B1
CYR61
CYTOR
DAAM2
DAB2
DACT3
DAPK3
DAXX
DDAH1
DDC
DDIT4
DDX17
DDX3X
DDX6
DEDD
DERL1
DFFA
DGAT1
DGUOK
DHFR
DHFRP1
DICER1
DIMT1
DIO1

DIRAS3
DKK1
DKK2
DKK3
DLL1
DLL4
DMD
DNAJA4
DNAJB11
DNAJC27
DND1
DNM1L
DNMT1
DNMT3A
DNMT3B
DOCK1
DOCK4
DOCK5
DOCK7
DOHH
DPYD
DPYSL2
DRAM2
DRD1
DSC2
DTD1
DTL
DTNB
DUSP1
DUSP10
DUSP2
DUSP4
DUSP5
DUSP6
DVL2
DYRK2
E2F1
E2F2
E2F3
E2F5
E2F6
EBP
ECT2
EDNRA
EED
EEF1A2
EFNA1
EFNA3

EGF
EGFR
EGLN1
EGLN3
EGR1
EGR2
EHD2
EID1
EIF2S1
EIF2S3
EIF4A2
EIF4E
EIF4EBP1
EIF5A2
ELAVL1
ELF2
ELN
EMSY
ENPEP
EOMES
EP300
EPAS1
EPB41L3
EPHA2
EPHA4
EPHA5
EPN2
EPO
EPOR
EPSTI1
ERBB2
ERBB3
ERBB4
ERCC1
ERG
ERRFI1
ESR1
ESR2
ESRRG
ETS1
ETV1
EWSR1
EYA2
EYA4
EZH2
F11R
FADD
FAF1

FAM129A
FAM160B2
FAM3C
FAM45A
FAM83F
FANCG
FANCM
Fas
FASLG
FASN
FASTK
FBN1
FBXO11
FBXO31
FBXO8
FBXW7
FEN1
FERMT2
FES
FGA
FGB
FGF11
FGF2
FGF21
FGF9
FGFR1
FGFR2
FGFR3
FGFRL1
FGG
FH
FHIT
FIS1
FKBP1B
FKBP5
FLI1
FLOT2
FLT1
FMN2
FMNL3
FMOD
FMR1
FNDC3B
FOS
FOSB
FOSL1
FOXA1
FOXA2

FOXC1
FOXD1
FOXJ3
FOXL2
FOXM1
FOXN3
FOXO1
FOXO3
FOXP1
FOXP3
FRAT1
FRAT2
FSCN1
FSTL1
FURIN
FUT4
FUT8
FXN
FYN
FZD3
FZD4
FZD5
FZD6
FZD7
G6PC
GAB1
GAB2
GABARAPL1
GALNT7
GAPDH
GAS1
GAS5
GATA2
GATA3
GATA4
GATA6
GCM1
GDAP1
GDF5
GEMIN4
GFRA3
GIT1
GJA1
GLI1
GLI2
GLS
GLS2
GLUL

GMFB
GNA13
GNAI1
GNAI2
GNAI3
GOLM1
GOLPH3
GP1BA
GPC1
GPD1L
GPR137B
GPR78
GPR85
GPS1
GRB10
GRB2
GRIN2A
GRM4
GRM7
GRN
GSK3B
GSR
GSS
H2AFX
HAND2
HAS2
HAX1
HBEGF
HBP1
HCC
HDAC1
HDAC11
HDAC2
HDAC4
HDAC6
HDGF
HECTD2
HES1
HGF
HGS
HIF1A
HIF1AN
HIF3A
HIP1R
HIPK1
HIPK2
HIPK3
HK2

HLA-G
HLTF
HMGA1
HMGA2
HMGB1
HMGB2
HMGB3
HMGCR
HMGN2
HMGXB4
HMOX1
HNF1B
HNF4A
HNF4G
HNRNPD
HNRNPK
HOTAIR
HOTTIP
HOXA1
HOXA10
HOXA5
HOXA9
HOXB3
HOXB4
HOXB5
HOXC13
HOXD10
HPGD
HRAS
HS3ST2
HSD17B1
HSP90AA1
HSP90B1
HSPA4
HSPA5
HSPB2
HSPB6
HTR2C
ICAM1
ICAM2
ICOSLG
ID4
IDH1
IER2
IFITM1
IFNAR1
IFNB1
IFNG

IFNR
IGF1
IGF1R
IGF2
IGF2BP1
IGF2BP3
IGFBP1
IGFBP3
IKBKB
IKBKE
IKBKG
IKZF1
IKZF2
IKZF3
IKZF4
IL10
IL11
IL12A
IL12B
IL13RA1
IL18
IL1A
IL1B
IL21R
IL25
IL32
IL34
IL4
IL6
IL6R
ILK
IMPA1
IMPDH1
IMPDH2
ING4
ING5
INHBB
INO80
INPP4B
INPP5A
INSIG1
INSR
IRAK1
IRAK2
IRAK4
IRF1
IRF2
IRF4

IRF5
IRS1
IRS2
IS2
ISCU
ITCH
ITGA11
ITGA3
ITGA5
ITGA6
ITGB1
ITGB3
ITGB4
ITGB8
ITIH5
JADE1
JAG1
JAK1
JAK2
JAZF1
JMY
JPH2
JPT1
KAT2B
KCMF1
KCNH1
KCNH2
KDM2A
KDM4A
KDM5B
KDM5C
KDR
KEAP1
KHSRP
KIAA0100
KIF1B
KIF22
KIF26B
KIT
KITLG
KLB
KLC2
KLF12
KLF13
KLF15
KLF2
KLF3
KLF4

KLF5
KLF6
KLF9
KLHL11
KLK10
KMT5A
KPNA2
KRAS
KREMEN1
KREMEN2
L1CAM
LACTB
LAMB3
LAMC1
LAMC2
LAMP1
LAMP2
LARP1
LASP1
LATS2
LCN2
LDHA
LDHB
LDLR
LEF1
LFNG
LGALS1
LGALS9
LGR4
LIF
LIFR
LIMK1
LIN28A
LIN28B
LIPA
LMO2
LOX
LOXL2
LOXL4
LPAR1
LPL
LRG1
LRP2
LRP5
LRP6
LRRC8A
LRRFIP1
LTF

LYPLA2
LZTS1
MACC1
MAD2L1
MAFB
MAGEA12
MAGEA2
MAGEA3
MAGEA6
MAGI2
MALAT1
MALT1
MAN1B1
MAOA
MAP2K1
MAP2K3
MAP2K4
MAP2K6
MAP3K11
MAP3K12
MAP3K2
MAP3K4
MAP3K5
MAP3K7
MAP3K9
MAP4K3
MAP4K4
MAP7
MAPK1
MAPK14
MAPK3
MAPK4
MAPK7
MAPK8
MAPK8IP1
MAPK9
MAPRE1
MARCKS
MAT1A
MAX
MBD1
MBD2
MBNL1
MBNL2
MBNL3
MCL1
MCM2
MCM3

MDM2
MDM4
MECOM
MECP2
MEF2C
MEF2D
MEG3
MEGF9
MEIS1
MEN1
MEOX2
MEPE
MERTK
MEST
MET
METTL13
MFF
MFN2
MGMT
MGST2
MIEN1
MIF
MITF
MIXL1
MLEC
MLH1
MMP1
MMP12
MMP13
MMP14
MMP15
MMP16
MMP2
MMP24
MMP26
MMP3
MMP7
MMP9
MNT
MPL
MPRIP
MRC1
MRE11
MRPS27
MSH2
MSH3
MSH6
MSLN

MSMO1
MST1
MT1M
MT2A
MTA1
MTA2
MTA3
MTAP
MTDH
MTHFR
MTMR14
MTMR3
MTOR
MTTP
MTUS1
MUC1
MUC13
MXD1
MXI1
MYB
MYBL1
MYC
MYCBP
MYCBP2
MYCN
MYD88
MYH1
MYH2
MYH4
MYL9
MYLIP
MYO5A
MYO6
MYOCD
MYRF
MYT1
N4BP1
NABP1
NAIP
NAMPT
NANOG
NASP
NAV3
NCAM1
NCAN
NCAPG
NCOA1
NCOA3

NCOA6
NCOR1
NCOR2
NCSTN
NDRG2
NDST1
NDUFA4
NECTIN4
NEDD4L
NEDD9
NEK6
NES
NET1
NEU1
NEUROD1
NF1
NF2
NFAT5
NFATC1
NFATC3
NFE2L2
NFIA
NFIB
NFIX
NFKB1
NFKB2
NFKBIA
NFKBIB
NGFR
NID1
NIPSNAP1
NIT1
NKD1
NKD2
NKIRAS2
NKRF
NLK
NLN
NLRC5
NLRP3
NMI
NOD2
NOS1
NOS2
NOS3
NOTCH1
NOTCH2
NOTCH3

NOX4
NPAS3
NPAT
NPEPL1
NPR1
NPTX1
NR1H4
NR1I2
NR2C2
NR2F2
NR3C1
NR4A2
NR5A2
NRAS
NRP1
NRP2
NT5E
NTF3
NTRK2
NTRK3
NUDT1
NUMB
NUP93
OPRM1
OSBPL2
OSBPL6
OSBPL8
OTUB1
OTUD7B
OXTR
P2RX7
P4HB
PA2G4
PAK1
PAK2
PAK4
PAK5
PAM
PAPPA
PARP1
PARP8
PATZ1
PAX3
PAX6
PAX7
PAX8
PBX2
PBX3

PBXIP1
PCBP1
PCBP2
PCGF2
PCGF5
PCNA
PCTP
PDCD4
PDE4A
PDE4B
PDGFA
PDGFB
PDGFC
PDGFRA
PDGFRB
PDIA3
PDLIM7
PDS5B
PEA15
PEBP1
PER1
PGR
PHB
PHF10
PHF8
PHLDB2
PHLPP1
PHLPP2
PIAS3
PICSAR
PIGF
PIK3C2A
PIK3CA
PIK3CB
PIK3CD
PIK3CG
PIK3R1
PIK3R2
PIK3R3
PIM1
PIN1
PINK1
PITX1
PKD1
PKD2
PKM
PKNOX1
PLAG1

PLAT
PLAU
PLAUR
PLCE1
PLK1
PLK2
PLOD2
PLOD3
PLXNB1
PLXNC1
PMEPA1
PODXL
POLD1
POLR3D
POLR3G
POR
POU2F2
POU4F2
POU5F1
PPARA
PPARG
PPARGC1A
PPIC
PPIF
PPM1B
PPM1D
PPM1F
PPM1K
PPP1CA
PPP1CC
PPP1R10
PPP1R13B
PPP2R2A
PPP2R5E
PPP3CA
PRAP1
PRDM1
PRDM4
PRDX3
PRDX6
PRKAA1
PRKAA2
PRKCD
PRKCE
PRKCH
PRKD1
PRKG1
PRKN

PRKRA
PRMT5
PROX1
PRRT2
PRRX1
PSAP
PSMD10
PSMD9
PTBP2
PTBP3
PTEN
PTENP1
PTGER2
PTGES2
PTGS2
PTH1R
PTK2
PTMS
PTP4A2
PTP4A3
PTPN1
PTPN11
PTPN13
PTPN14
PTPN2
PTPN22
PTPN9
PTPRF
PTPRJ
PTPRN2
PTPRO
PTTG1
PTX3
PUM2
PURA
PVT1
PXDN
PXN
QKI
RAB11A
RAB11FIP1
RAB12
RAB13
RAB14
RAB15
RAB1A
RAB27A
RAB38

RAB40C
RAB5A
RAB5B
RAC1
RAD21
RAD51
RAD52
RAF1
RALA
RAN
RAP1B
RARA
RARB
RASA1
RASAL2
RASGRP1
RASSF1
RASSF6
RASSF8
RAVER2
RAX
RB1
RB1CC1
RBL1
RBL2
RBP2
RCAN1
RCBTB1
RCC2
RCOR1
RDX
RECK
REG4
RELA
RELN
REST
RET
REV3L
RFFL
RFX6
RFX7
RGMA
RGS16
RGS2
RGS3
RGS5
RHO
RHOA

RHOB
RHOBTB1
RHOC
RICTOR
RMND5A
RNASEL
RND3
RNF11
RNF144B
RNF2
ROBO1
ROBO2
ROCK1
ROCK2
ROR1
RPA1
RPS6KA1
RPS6KA3
RPS6KA4
RPS6KA5
RPS6KB1
RPS7
RREB1
RRM2
RSU1
RTKN
RTN4
RUNX1
RUNX2
RUNX3
RXRA
S100A1
S100A8
S100B
S1PR1
SAMHD1
SAPCD2
SATB1
SATB2
SAV1
SCARB1
SCD
SCNN1A
SCRIB
SDC1
SDHA
SDHD
SELE

SEMA3A
SEMA4C
SEMA4D
SEMA6A
SEMA7A
SENP1
SEPT7
SERBP1
SERINC5
SERPINB5
SERPINB9
SERPINE1
SERPINF2
SERPINH1
SERPINI1
SET
SETD2
SETDB1
SFPQ
SFRP1
SFRP2
SFRP5
SGPL1
SGPP2
SH3BGRL
SH3GL1
SH3PXD2A
SHC1
SHMT2
SIAH2
SIGLEC1
SIKE1
SIRPA
SIRT1
SIRT2
SIRT6
SIRT7
SIVA1
SIX1
SKI
SKP2
SLAIN1
SLC16A1
SLC16A2
SLC1A2
SLC22A7
SLC2A1
SLC2A3

SLC2A4
SLC45A3
SLC5A5
SLC6A4
SLC6A8
SLC7A11
SLC7A6
SLPI
SMAD1
SMAD2
SMAD3
SMAD4
SMAD5
SMAD7
SMARCA2
SMARCA4
SMARCA5
SMN1
SMO
SMR3B
SMURF1
SNAI1
SNAI2
SNAI3
SND1
SNIP1
SOCS1
SOCS3
SOCS5
SOCS6
SOCS7
SOD2
SOD3
SOS1
SOST
SOX17
SOX2
SOX4
SOX5
SOX6
SOX9
SP1
SP3
SP4
SP7
SPARC
SPHK1
SPI1

SPRED1
SPRED2
SPRY1
SPRY2
SPRY3
SPRY4
SPTBN1
SPTLC1
SRC
SREBF1
SREBF2
SRF
SRGAP1
SRGAP2
SRR
SRSF1
SRSF10
SRSF11
SSSCA1
SSX2IP
ST14
ST3GAL6
ST7L
ST8SIA4
STAG2
STAM2
STARD13
STAT1
STAT3
STAT5A
STAT5B
STAT6
STK11
STK40
STMN1
STRADB
STUB1
STX16
STX1A
SUFU
SUV39H1
SUZ12
SWAP70
SYK
SYT1
TAB1
TAB2
TAB3

TAC1
TACC1
TACC3
TAL1
TAP1
TARBP1
TBC1D1
TBC1D2
TBC1D9
TBCCD1
TBK1
TBX21
TBXA2R
TCEAL1
TCEAL9
TCF21
TCF3
TCF4
TCF7
TCL1A
TDG
TERF2
TERT
TET1
TET2
TET3
TEX261
TF
TFAM
TFAP2A
TFAP2C
TFEB
TFF1
TFF2
TFPI
TFRC
TGFA
TGFB1
TGFB2
TGFB3
TGFBI
TGFBR1
TGFBR2
TGFBR3
TGFBRAP1
TGIF1
TGIF2
THAP2

THBS1
THBS2
THRB
THSD7A
TIAM1
TICAM1
TICAM2
TIMP1
TIMP2
TIMP3
TIRAP
TJAP1
TLE3
TLN2
TLR2
TLR3
TLR4
TLR7
TM9SF3
TMC7
TMED7
TMEM2
TMEM64
TMEM92
TMEM9B
TMOD3
TNC
TNF
TNFAIP3
TNFRSF10A
TNFRSF10B
TNFRSF12A
TNFSF10
TNFSF11
TNFSF12
TNFSF13
TNK2
TNRC6A
TOB1
TOP1
TOPORS
TOX
TP53
TP53BP2
TP53COR1
TP53I11
TP53INP1
TP63

TP73
TPD52
TPM1
TPM3
TPPP3
TPRG1
TRA2B
TRAF3IP2
TRAF4
TRAF5
TRAF6
TRAF7
TRAPPC2B
TREM2
TRIB1
TRIB2
TRIB3
TRIM11
TRIM27
TRIM29
TRIM68
TRIM71
TRIM8
TRPC5
TRPC6
TRPS1
TSC1
TSG101
TSPAN6
TTK
TUBB4B
TUG1
TUSC2
TUSC3
TWF1
TWIST1
TWIST2
UBE2C
UBE2F
UBE2I
UBE2N
UBE3C
UBR5
UCA1
UCP1
UGT2B15
UGT2B17
UHRF1

UHRF2
ULBP2
ULK1
ULK2
UNG
USF2
USP14
USP18
USP4
USP9X
UVRAG
VAMP2
VAV2
VDAC1
VDR
VEGFA
VEGFC
VEZT
VGLL4
VHL
VIM
VLDLR
VMP1
VOPP1
VPS4B
VPS51
WASF2
WASF3
WDR4
WDR77
WEE1
WIF1
WNK1
WNK4
WNT1
WNT10B
WNT3A
WNT4
WNT7A
WT1
WWP1
XBP1
XIAP
XIST
XPA
XRCC3
XRCC5
YAP1

YBX1
YBX3
YES1
YOD1
YWHAZ
YY1
YY1AP1
ZAP70
ZBTB10
ZBTB2
ZBTB4
ZBTB7A
ZCCHC11
ZDHHC9
ZEB1
ZEB2
ZFP36
ZFYVE9
ZHX1
ZNF215
ZNF217
ZNF763
ZNFX1
ZNRF2
ZNRF3
ZWINT
ZYX

Targets of Upregulated hASC-EVs miRNAs	Targets of Upregulated hAMSC-EVs miRNAs
ABL1	ABCB1
ABTB1	ABCC1
ADAM12	ABCG2
ADGRA2	ACVR1C
ADORA2B	ADAR
AGO1	ADRB1
AHRR	AGO2
AKT2	AIFM3
AKT3	AKAP12
ANGPT2	ALDH5A1
AP2M1	ANG
APAF1	APP
API5	AQP1
APLN	AR
ARHGEF3	ARF1
ARID3A	ARHGAP5
ARID3B	ARID4B
ARL2	ARPC5L
ATAT1	ARPP19
ATF1	ATG16L1
ATG14	ATG4C
AVEN	ATG7
BAG1	ATM
BAK1	ATP7A
BCL11A	ATXN1
BCL2L1	BANP
BCL9	BASP1
BLCAP	BCL10
BMF	BCL2L11
BMP1	BCLAF1
BMPR1B	BIRC6
BTG2	BMI1
BTRC	BMP2
CAB39	BMP7
CASP3	BMPR2
CBFB	BNIP3
CBX3	BRCA1
CBX4	BRCA2
CCL4	BTK
CCND3	CARD10
CCNE1	CASP8
CCNE2	CASP8AP2
CCNG1	CCL1
CCNJ	CCL2

CCNT1	CCL5
CD274	CD22
CD44	CD40LG
CD99	CD80
CDC25A	CDC14A
CDC27	CDK2
CDC42	CDK9
CDH1	CDKN1A
CDH11	CDKN3
CDH5	CDX2
CDK4	CERS2
CDK8	CFH
CDKN2A	CLOCK
CDKN2D	CLTC
CEBPA	CLU
CGN	CNOT6L
CHEK1	COX2
CHUK	CPEB2
COL10A1	CPM
COL1A1	CRKL
COL3A1	CTBP2
COL4A1	CTDSP2
COL5A2	CTNNB1
COL5A3	CUL2
COPS6	CUL5
CREB1	CXCL2
CRISP2	CYP19A1
CTNNBIP1	CYP7B1
CX3CL1	CYR61
CYP1B1	DAAM2
CYP24A1	DAB2
CYP3A4	DAPK3
CYTOR	DDAH1
DGAT1	DIMT1
DICER1	DKK1
DIO1	DNAJC27
DKK2	DUSP1
DKK3	E2F1
DNAJB11	EEF1A2
DNMT3A	EFNA3
DNMT3B	EGR1
DPYD	EGR2
DRAM2	EHD2
DTL	EP300
DUSP2	EPAS1
DUSP6	EPN2
E2F2	EPSTI1
EDNRA	ERBB4

EED	ERCC1
EID1	ESRRG
EIF4EBP1	ETV1
EIF5A2	FADD
ELN	FAF1
ENPEP	FANCM
EPO	FASLG
EPOR	FASTK
ERBB3	FBXO31
ERG	FGF21
ESR2	FGFR1
ETS1	FGFRL1
EYA2	FH
EYA4	FIS1
EZH2	FMN2
FANCG	FMNL3
FBN1	FOXA2
FBXW7	FOXM1
FERMT2	FOXN3
FES	FOXP1
FGA	FOXP3
FGB	GCM1
FGFR2	GEMIN4
FGG	GIT1
FOS	GNAI1
FOSB	GOLPH3
FOSL1	GPC1
FOXD1	GPD1L
FOXJ3	GPR137B
FOXL2	GRM4
FSTL1	HBP1
FZD5	HIF3A
FZD6	HIPK1
FZD7	HIPK3
G6PC	HMGB1
GAB2	HNRNPD
GATA2	HOXA1
GATA3	HOXA5
GLI1	HOXA9
GLS	HSD17B1
GNAI2	HSPB2
GPS1	HSPB6
GRIN2A	ICAM1
GRN	ID4
GSS	IGFBP3
H2AFX	IKBKG
HCC	IL1A
HDAC4	INPP5A

HES1	IRAK1
HIP1R	IRAK2
HIPK2	IS2
HK2	ISCU
HMGA1	ITGB8
HMGB2	JAK1
HMGB3	KAT2B
HMGCR	KCMF1
HMGN2	KIF22
HNF1B	KIT
HNF4G	KITLG
HOTTIP	KLF15
HOXB4	L1CAM
HSP90AA1	LATS2
HSPA4	LFNG
ICAM2	LIMK1
IFNG	LRG1
IFNR	LRP2
IGF1	LRRC8A
IGF2	MAGI2
IKZF2	MALAT1
IKZF3	MAP3K12
IKZF4	MAP3K2
IL21R	MAP3K5
IL32	MAPK1
IL6R	MAPK9
INSR	MAT1A
IRF1	MCM3
IRF4	MDM2
ITGA6	MDM4
JAK2	MECP2
KDM2A	MEF2D
KDM5C	MEIS1
KDR	MEOX2
KHSRP	MEPE
KIAA0100	MERTK
KIF1B	MFN2
KLC2	MGST2
KLF13	MIF
KLF3	MMP16
KLF9	MMP3
KLK10	MNT
LACTB	MRE11
LAMC1	MSMO1
LAMP1	MTA2
LAMP2	MTA3
LASP1	MTHFR
LCN2	MTUS1

LIFR
LIN28A
LIN28B
LIPA
LOX
LOXL2
LOXL4
LPAR1
LRP5
LRP6
MAN1B1
MAP2K4
MAP3K11
MAP4K3
MAPK14
MBD1
MBD2
MBNL1
MBNL2
MBNL3
MEF2C
MEGF9
MEN1
MET
MFF
MMP13
MMP15
MMP24
MMP26
MMP9
MPL
MRC1
MT2A
MTOR
MUC1
MYH1
MYH2
MYH4
MYT1
NASP
NCOA6
NCOR2
NEDD4L
NEK6
NES
NEU1
NEUROD1
NFE2L2
MYD88
MYLIP
NABP1
NANOG
NCSTN
NDST1
NDUFA4
NFAT5
NFATC3
NFKBIA
NKRF
NOD2
NOS1
NOTCH2
NPAS3
NPAT
NPEPL1
NPR1
NPTX1
NR4A2
NRP1
OPRM1
P2RX7
P4HB
PA2G4
PAK5
PARP1
PAX8
PBX3
PDCD4
PDLIM7
PICSAR
PIK3CA
PIM1
PITX1
PKD2
PKNOX1
PLAUR
PLK1
PMEPA1
POLR3D
POU5F1
PPM1B
PPM1F
PPP2R2A
PRKAA1
PRKCE
PRMT5

NID1
NIT1
NKIRAS2
NR2F2
NRAS
NT5E
NTRK3
OSBPL6
PATZ1
PAX3
PAX7
PBX2
PCTP
PDGFA
PDGFB
PDGFC
PDGFRB
PDS5B
PEBP1
PER1
PHB
PHF8
PIAS3
PIGF
PIK3CB
PIK3CD
PIK3CG
PIK3R2
PINK1
PLAG1
PLK2
PODXL
POU4F2
PPARG
PPARGC1A
PPIC
PPP1CA
PPP1R13B
PRDM1
PRKAA2
PRKRA
PRRT2
PTH1R
PTK2
PTPN11
PTX3
RAB38
RAF1
PTBP3
PTENP1
PTGER2
PTGES2
PTGS2
PTP4A3
PTPN1
PTPN2
PTPRN2
PTPRO
PTTG1
PUM2
PURA
PVT1
RAB11A
RAB11FIP1
RAB13
RAB14
RAB27A
RAD21
RAD52
RARB
RASA1
RB1
RBL1
RBL2
RELA
RHO
RHOA
RHOB
RND3
RNF11
ROCK1
RPS6KA3
RPS6KA4
RUNX3
SATB1
SDHD
SELE
SERPINE1
SETD2
SH3BGRL
SIAH2
SIKE1
SIRPA
SIVA1
SLC16A1
SLC16A2

RARA
RASSF8
RAVER2
RAX
RELN
RET
RGS5
RICTOR
RMND5A
ROCK2
ROR1
RPA1
RPS6KA1
RPS6KB1
S100A1
S100B
SCNN1A
SEMA4C
SEMA6A
SEMA7A
SENP1
SEPT7
SERPINF2
SERPINH1
SET
SFPQ
SFRP5
SGPL1
SHC1
SIGLEC1
SIRT1
SIRT7
SKP2
SLC45A3
SLC6A8
SLC7A11
SMAD1
SMAD3
SMAD5
SMO
SNAI1
SNAI2
SNAI3
SP3
SP4
SPARC
SPHK1
SPRY2
SLC2A4
SLC5A5
SLC7A6
SLPI
SMN1
SMR3B
SMURF1
SNIP1
SOCS1
SOCS3
SOCS6
SOS1
SOX17
SOX2
SREBF1
SREBF2
SRSF11
STAT1
STAT5B
STK11
STMN1
SYK
TAB2
TAC1
TBC1D2
TBC1D9
TCEAL9
TCF3
TF
TFF2
TFRC
TGFA
TGFBR2
THSD7A
TIMP2
TIMP3
TLE3
TLR2
TLR4
TLR7
TMC7
TMEM2
TNC
TNFRSF12A
TNFSF12
TP53COR1
TP53I11
TRAF6

ST14
ST7L
STARD13
SUV39H1
TBC1D1
TBCCD1
TBX21
TCL1A
TDG
TERF2
TET1
TET2
TFPI
TGFB2
TGFB3
THBS2
THRB
TMEM64
TNFRSF10B
TNRC6A
TOP1
TPD52
TRAF3IP2
TRIB1
TRIB2
TRIM27
TRIM71
TSC1
TUBB4B
TWIST2
UBE3C
UCA1
VDR
VEGFC
VPS51
WDR77
WNK1
WNT7A
YAP1
ZBTB10
TRIM29
TRIM8
TRPC5
TTK
TUG1
TUSC2
TWIST1
UBE2C
UBE2F
UGT2B15
UGT2B17
UHRF1
ULK1
USP14
UVRAG
VDAC1
VEZT
VLDLR
VMP1
WASF2
XBP1
XIST
XPA
YBX3
YES1
ZBTB2
ZBTB4
ZDHHC9
ZEB1
ZFYVE9
ZNFX1
ZNRF3

Supplementary Table 6. hASC-EVs and hAMSC-EVs first quartile shared miRNAs stability ranking.

	Genorm M-value	Normfinder SV	BestKeeper SD	Delta CT SD	Geomean
miR-34a-5p	0.27	0.25	0.28	0.87	1.73
miR-24-3p	0.00	0.34	0.44	0.89	3.66
miR-20a-5p	0.36	0.31	0.50	0.87	4.52
miR-127-3p	0.00	0.34	0.44	0.89	4.53
miR-30c-5p	0.57	0.35	0.28	0.89	5.59
miR-99a-5p	0.46	0.37	0.28	0.90	6.09
miR-28-3p	0.41	0.35	0.44	0.90	7.65
miR-331-3p	0.44	0.45	0.33	0.94	7.90
miR-365a-3p	0.64	0.58	0.28	0.98	8.60
miR-106b-5p	0.58	0.59	0.28	1.00	10.03
miR-193a-5p	0.59	0.40	0.44	0.91	12.19
miR-22-3p	0.52	0.48	0.44	0.95	12.57
miR-21-5p	0.50	0.53	0.44	0.97	12.92
miR-130b-3p	0.65	0.56	0.33	0.98	13.63
miR-100-5p	0.55	0.54	0.44	0.97	14.17
miR-30b-5p	0.61	0.51	0.44	0.96	14.34
miR-152-3p	0.48	0.52	0.50	0.98	14.58
miR-382-5p	0.66	0.56	0.33	0.98	16.43
miR-132-3p	0.63	0.49	0.50	0.96	16.47
miR-197-3p	0.67	0.68	0.33	1.04	19.04
miR-26b-5p	0.54	0.62	0.50	1.00	19.06
miR-214-3p	0.70	0.71	0.33	1.07	20.45
miR-92a-3p	0.68	0.68	0.33	1.04	20.58
miR-99b-5p	0.62	0.67	0.44	1.02	21.07
miR-193b-3p	0.70	0.71	0.33	1.07	22.33
miR-16-5p	0.75	0.70	0.33	1.07	23.50
miR-25-3p	0.68	0.71	0.44	1.05	25.09
miR-145-5p	0.69	0.68	0.56	1.06	27.24
miR-221-3p	0.71	0.69	0.50	1.06	27.50
miR-218-5p	0.72	0.76	0.44	1.09	28.07
miR-532-5p	0.77	0.64	0.67	1.03	28.18
miR-222-3p	0.76	0.67	0.56	1.05	28.72
miR-199a-3p	0.73	0.81	0.33	1.12	28.85
miR-31-3p	0.74	0.71	0.50	1.07	29.46
miR-296-5p	0.72	0.76	0.56	1.10	33.16
miR-149-5p	0.79	0.75	0.83	1.08	36.55
miR-30e-3p	0.78	0.77	0.83	1.08	36.61
miR-29a-3p	0.86	0.80	0.67	1.14	40.15
miR-29c-3p	0.87	0.94	0.56	1.20	41.18
miR-210-3p	0.82	0.86	1.00	1.11	41.63
miR-320a-3p	0.84	0.86	1.00	1.11	41.94
miR-574-3p	0.85	0.86	1.00	1.11	42.37
miR-30a-3p	0.81	0.86	1.00	1.11	42.52
miR-17-5p	0.83	0.86	1.00	1.11	43.03
miR-26a-5p	0.88	0.94	0.67	1.21	43.45
miR-28-5p	0.91	1.01	0.67	1.26	44.74
miR-27b-3p	0.89	1.05	0.67	1.26	45.33
miR-130a-3p	0.90	1.05	0.67	1.26	46.61
miR-10a-5p	0.92	0.97	0.83	1.26	46.73
miR-328-3p	0.93	1.03	0.83	1.29	48.17
miR-376a-3p	0.94	1.03	1.00	1.26	48.72
miR-484	0.95	1.10	1.17	1.30	53.67

miR-30a-5p	1.03	1.35	0.89	1.51	54.73
miR-191-5p	0.97	1.12	1.17	1.32	54.93
miR-376c-3p	0.96	1.13	1.17	1.32	54.97
miR-409-3p	1.00	1.16	1.00	1.40	54.97
miR-106a-5p	0.99	1.13	1.17	1.32	56.18
miR-125b-5p	1.04	1.44	1.00	1.59	58.19
miR-19b-3p	1.01	1.32	1.33	1.47	58.21
miR-224-5p	1.06	1.45	1.00	1.62	58.89
miR-31-5p	1.08	1.39	1.11	1.60	59.23
miR-181a-5p	1.10	1.43	1.11	1.63	59.95
miR-27a-3p	1.12	1.80	1.33	1.90	63.00
miR-335-5p	1.15	1.96	1.83	2.06	64.00
